# Taxonomic and identification review of adventive *Fiorinia* Targioni Tozzetti (Hemiptera, Coccomorpha, Diaspididae) of the United States

**DOI:** 10.3897/zookeys.1065.69171

**Published:** 2021-10-27

**Authors:** Muhammad Z. Ahmed, Matthew R. Moore, Eric A. Rohrig, Cindy L. McKenzie, Di Liu, Jinian Feng, Benjamin B. Normark, Douglass R. Miller

**Affiliations:** 1 Subtropical Insects and Horticulture Research, Agricultural Research Service, United States Department of Agriculture, 2001 South Rock Road, Fort Pierce, Florida, USA; 2 Florida State Collection of Arthropods, Division of Plant Industry, Florida Department of Agriculture and Consumer Services, Gainesville, Florida, USA; 3 Molecular Diagnostics Laboratory, Division of Plant Industry, Florida Department of Agriculture and Consumer Services, Gainesville, Florida, USA; 4 Key Laboratory of Plant Resources and Pest Management, Ministry of Education, Entomological Museum, College of Plant Protection, Northwest A & F University, Yangling, Shaanxi Province, China; 5 Department of Biology and Graduate Program in Organismic and Evolutionary Biology, University of Massachusetts, Amherst, Massachusetts, USA; 6 Retired Research Entomologist, Systematic Entomology Lab, Agricultural Research Service, United States Department of Entomology, Beltsville, Maryland, USA

**Keywords:** Armored scale insects, DNA barcodes, Florida, palms, phantasma scale, slide mounting

## Abstract

This work provides general descriptions, illustrations, molecular diagnostic data, taxonomic keys, slide mounting recommendations, and Florida distribution records for *Fiorinia* Targioni Tozzetti species occurring in the USA. Species treated are *F.externa* Ferris, *F.fioriniae* (Targioni Tozzetti), *F.japonica* Kuwana, *F.pinicola* Maskell, *F.phantasma* Cockerell & Robinson, *F.proboscidaria* Green, and *F.theae* Green. New descriptions of second-instar males and females of all seven species in addition to first-instar nymphs and adult females of *F.phantasma* and *F.proboscidaria* are presented. Taxonomic keys to second-instar males and females are developed for the first time and previously available taxonomic keys to first-instar nymphs and adult females are improved. DNA sequences were used to further evaluate the monophyly of *Fiorinia* and provide additional diagnostic tools for *Fiorinia* species. Multigene phylogenetic analyses, COI barcoding methods, and examination of type material indicate that *F.yongxingensis* Liu, Cai & Feng, 2020, **syn. nov**. is a junior synonym of *F.phantasma*. A morphological survey of the genus demonstrates, for the first time, the utility of second-instar males for diagnostics. This study will help inform regulatory and pest management decisions by facilitating morphological and molecular identification of adventive *Fiorinia* species occurring in the USA.

## Introduction

The genus *Fiorinia* (Hemiptera, Diaspididae) comprises 70 species (García Morales et al. 2016) apparently native to Asia (Williams and Watson 1988). The genus, as presently defined, appears to represent a monophyletic group, according to a recent molecular phylogenetic analysis (Normark et al. 2019). Species in the genus are pupillarial; i.e., the adult female remains inside the exuviae of the second-instar female and does not form a scale cover. Seven species have been reported to cause economic damage, including *F.externa* Ferris, 1942 (McClure 1977), *F.fioriniae* (Targioni Tozzetti, 1867) (Beardsley and González 1975), *F.japonica* Kuwana, 1902 (Tang 1984), *F.phantasma* Cockerell & Robinson, 1915 (Ahmed 2018; Liu et al. 2020), *F.pinicola* Maskell, 1897 (Miller and Davidson 1990), *F.proboscidaria* Green, 1900 (Ahmed and Stocks 2020), and *F.theae* Green, 1900 (Gill 1997). Unfortunately, all seven species have been introduced into the USA during successive waves of invasion. *Fioriniafioriniae*, *F.phantasma*, *F.proboscidaria*, and *F.theae* are established in Florida; *F.externa* is commonly intercepted in Florida but has not become established (Suppl. material 1: Fig. S1).

*Fioriniaphantasma*, commonly known as phantasma scale, was described from the Philippine Islands in 1915. Subsequently, a major global expansion of *F.phantasma* occurred over the last decade through movement of nursery stock (Watson et al. 2015). *Fioriniaphantasma* is now documented from 19 countries (China (Hong Kong, Mainland China, Taiwan), France, French Polynesia, Grenada, Indonesia, Malaysia, Maldives, Nauru, Netherlands, New Caledonia, Papua New Guinea, Philippines, Reunion, Saint Barthelemy and Saint Martin, Singapore, Solomon Islands, Thailand, United States (American Samoa, Florida, Hawaii, Guam), and Vietnam). In some areas, *F.phantasma* may reach heavy infestations causing serious plant damage (Watson et al. 2015; García Morales et al. 2016). In one particularly impactful infestation of *F.phantasma*, approximately 6,000 palms were severely infested and declining at a resort in the Maldives (Watson et al. 2015). A polyphagous pest, *F.phantasma* has been reported on 25 families and 56 genera of hosts, including many nursery and ornamental plants, particularly palms, as well as several fruit crops (Watson et al. 2015; García Morales et al. 2016; Ahmed et al. 2021). For the nursery and greenhouse sector, palms account for sales of approximately $400 million annually in Florida and well over $1 billion annually in the USA (Khachatryan and Hodges 2012). Scale insects feed on all parts of their host plants, but *F.phantasma* is common on leaves, causing chlorosis, leaf drop, and ultimately plant death. This pest has the potential to cause economic harm in the USA to nurseries, landscape industries, and homeowners.

The first North American continental report of *F.phantasma* was in Florida and included more than twenty heavily infested Canary Island date palms (*Phoenixcanariensis* Chabaud) along both sides of a road in Miami-Dade County (Ahmed 2018). The population was likely there for some time, considering the density of the scales and the presence of specimens on many trees. It is not surprising that the Florida infestation was not detected earlier because the scale is identical in field appearance to other *Fiorinia* species that occur in Florida (Ahmed 2018). *Fiorinia* species infestations start with the arrival of crawlers (first-instar nymphs), either by wind, or via infested plant material or garden tools because crawlers constitute the only mobile stage besides adult males, which do not feed. Crawlers settle on plant parts and molt into second-instar males and females within a few days.

The main pest management challenge is detection of new *F.phantasma* infestations. *Fioriniaphantasma* occurs in two Florida counties, Miami-Dade and Palm Beach, and is usually found on palms (FDACS-DPI Entomology Database 2021). Detection is complicated by the presence of *F.fioriniae*, which is commonly found on palms throughout most of Florida (FDACS-DPI Entomology Database 2021). *Fioriniajaponica*, another morphologically and behaviorally similar species, also infests palms, but is only found in California and several east coast states in the USA. Should *F.japonica* become established in Florida, it would be difficult to detect because the species looks identical in the field to the other *Fiorinia* species infesting palms. Heavy infestations of another *Fiorinia* species, *F.proboscidaria*, were recently recorded on citrus from residential areas in Florida. Regulatory efforts aimed at preventing its introduction to and establishment in commercial citrus growing areas in Florida are being implemented (Ahmed and Stocks 2020). To date, the only way to identify these species has been to mount adult females on a microscopic slide and examine them with a compound microscope. The regulatory and pest management situation surrounding *Fiorinia* species in the USA, and especially Florida, is dynamic and subject to identification challenges. Thus, it is important to develop identification tools for *Fiorinia* adult females and other commonly collected life stages using diagnostic molecular and morphological data. Without reliable and correct identification, one cannot properly make regulatory and control decisions.

The purpose of this study is to provide taxonomic keys for immatures of seven *Fiorinia* species occurring in the USA. We also provide line drawings and diagnoses of slide-mounted second-instar males and females, DNA sequence data for multiple loci for molecular diagnostics, and extensive records of the species’ distributions in Florida. We newly describe and illustrate first-instar nymphs and adult females of two species, *F.phantasma* and *F.proboscidaria*. In addition, we provide updated taxonomic keys for first-instar nymphs (adapted from Howell 1977) and adult females (Watson et al. 2015).

## Materials and methods

### Taxon sampling

Four species of *Fiorinia* (*F.fioriniae*, *F.phantasma*, *F.proboscidaria*, *F.theae*) were collected from Florida (Suppl. material 2: Table S1). *Fioriniaexterna* samples were collected from Christmas trees imported from outside of Florida. First- and second-instar nymphs and adult females from infested plant materials were preserved in 100% ethanol for slide mounting and molecular analysis. *Fioriniajaponica* and *F.pinicola* specimens were borrowed from the United States National Museum of Natural History, scale insect collection, Beltsville, Maryland (USNM). *Fioriniapinicola* specimens were provided to us by Natalia von Ellenrieder (California Department of Food and Agriculture) (Suppl. material 2: Table S1). The details for specimens examined for description and diagnosis is provided in the figure captions of each species. Due to regulatory issues surrounding *F.yongxingensis* Liu, Cai & Feng, 2020 from Hainan, China, its DNA sequences were obtained in China by one of us (DL). All samples were initially mounted in Hoyer’s medium for visibility during illustration and were transferred to balsam medium for permanent preservation. This was done by placing the Hoyer’s slide in a petri dish filled with water, just enough that the slide is slightly submerged, for a few hours depending on the age of the Hoyer’s slide. Once the slide cover is detached and loosened, it can easily be removed. The specimen can then be removed from the slide without being damaged. Specimens were soaked in a watch glass filled with water overnight to rinse Hoyer’s media out of the specimen. After soaking, specimens were placed on a new slide with a drop of balsam and covered with a new coverslip. Illustrations were made using a Leica DMRB compound microscope and a camera lucida. Morphological terminology follows that of Miller and Davidson (2005). Numerical values were taken from a minimum of five specimens, if available, from as many Florida localities as possible. All specimens were deposited in the Florida State Collection of Arthropods, Gainesville (**FSCA**) unless otherwise indicated. Other depositories included **USNM** (United States National Museum of Natural History, scale insect collection, Beltsville, Maryland), **UMEC** (University of Massachusetts Entomology Collection, Amherst, Massachusetts), and Entomology Museum, Northwest Agricultural and Forestry University, Shaanxi, China.

In addition to the freshly collected *Fiorinia* specimens described above, additional specimens and sequences were included in analyses of DNA sequences (Suppl. material 2: Table S1): fresh specimens of the outgroups *Thysanofiorinialeei* Williams and *T.nephilii* (Maskell) collected in Florida; ethanol-preserved specimens of *Fiorinia* sp. collected in Lambir Hills National Park, Malaysia, in 2013; cytochrome oxidase I (COI) sequences of Diaspididae from the BOLD database (Ratnasingham and Hebert 2007), along with one sequence of *Pseudococcus* sp. (BOLD record AMSMB002-15; BIN BOLD:ACZ2386) as an outgroup; and cytochrome oxidase I and II (COI-II), elongation factor 1a (EF1a), and large ribosomal subunit (28S) sequences of the genus *Fiorinia* reported in Normark et al. (2019), along with exemplars of other species of Fioriniina and one sequence of *Unaspisyanonensis* (Kuwana) as an outgroup.

### Slide mounting of immature stages

Slide mounting is considered mandatory for morphological identification of armored scale insects because it is nearly impossible to identify taxonomic features without doing so. Moreover, for museum curation purposes, slide mounting is the best way to archive scale insects in a reference collection. There are studies available on methods for mounting hemipterans (Hodges and Evans 2005), but many are not specific to scale insects or armored scales (Wirth and Marston 1968). Previously published mounting methods for scale insects (McKenzie 1957; Wilkey 1990; Watson 2002) need to be reevaluated to meet the need for rapid identification as pest species are spreading swiftly through national and international trade. Recently published methods have focused on modifying slide mounting to enhance safety since the reagents can be corrosive, flammable, or carcinogenic, or can produce toxic fumes (Sirisena et al. 2013). Another recent study modified the watch glass with a sieve to process specimens in a shorter period (Barbecho and Lit 2015). Nevertheless, a reliable protocol for slide mounting of immature armored scale insects still needs to be established. Mounting methods are also biased towards adult scales, despite the importance of first and second-instar nymphs to armored scale biology. These immature life stages are commonly found in the field, but are taxonomically studied to a much lesser degree than adults. We evaluated several methods to enhance safety and reduce the time required to mount fresh and absolute ethanol-preserved specimens of first- and second-instar nymphs of *Fiorinia* species.

#### (i) Standard slide mounting method (6 steps)

Initially, a 67 mm beveled-edge watch glass (Prolab Scientific) and micro spatula were used. Fisher 10% potassium hydroxide (KOH) was used in step 1 for heating and maceration. Following this step, specimens were placed into a Humboldt mesh (Replacement Mesh Disk 5 cm dia. No. 325H-3807.325) container that was then placed inside the watch glass, eliminating the need for the micro spatula in the following steps until the final mount. Forceps (Bioquip Swiss style #4) were used to remove the mesh from the 4.8 cm watch glass (Item#742300, Carolina) while switching between steps. Glacial acetic acid (Fisher) was used in step 2 for removal of the remaining 10% KOH from step 1. Acid fuchsin stain (Bioquip) was used with a 3:7 dye to acetic acid ratio in step 3 to stain the specimens. For dehydration of the cuticle in the 4^th^ step, 75% and 95% EtOH were used. Clove oil (Spectrum Chemical) was used in step 5 to remove any remaining wax from the specimens. A disposable transfer pipette (13-711-9D, Fisher) which holds 3.2 ml, was used in steps 2–5. In the final step 6 filtered Canada balsam (CAS 8007-47-4, Fisher) was used as the medium and placed on a glass slide (22-038-103, Fisher). A glass coverslip (12-545-80P, Fisher) was placed on the specimen in balsam to complete the mount. The 6 steps required for the standard slide mounting method are as follows:

1. Heating: specimens were set in a watch glass filled with 10% KOH and heated at 85 °C for 5–10 mins. After heating, gut contents were teased out using a micro-spatula to gently tap the dorsum.
2. KOH removal: specimens were moved to a watch glass of 95% glacial acetic acid for 10 mins to remove any remaining KOH.
3. Staining: Acid fuchsin stain was added and let sit for 5 mins.
4. Stain correcting: specimens were moved to a watch glass of 75% EtOH for 10 mins. Specimens were then placed in 95% EtOH for another 10 mins to dehydrate.
5. Wax removal: specimens were soaked in clove oil for 5 to 10 mins. This helps to remove any remaining wax or lipids and makes specimen bodies flexible to be easily spread on a slide.
6. Mounting: on a labeled slide, a drop of balsam was placed in a center and spread to avoid specimen drift. The specimen was then placed in the balsam dorsoventrally (i.e. ventral side up) and legs and antennae were positioned properly. A coverslip was placed on the balsam, and the slide was placed on a hot plate at 30 °C for 10 mins to remove any bubbles.

Due to the multiple steps in this method, which require each specimen to be moved from 5 different watch glasses before mounting, many first-instar nymphs were lost or damaged. Additionally, this method was time consuming. In an attempt to reduce the loss of first-instar nymphs, minimize damage, reduce the amount of chemical usage, and save time, we subsequently developed alternative methods – see below.

#### (ii) Modified slide mounting method A (1 step)

For fresh specimens (not preserved in ethanol).

1. Mounting: on a labeled slide, a drop of Hoyer’s medium was placed in the center and spread to avoid specimen drift. Fresh specimens picked from plant material were placed in a Hoyer’s medium dorsoventrally and legs and antennae were positioned properly. In this protocol, we omitted steps 1–5 of the standard method and mounted specimens directly into Hoyer’s medium. This was effective in preventing loss of specimens and reducing the amount of chemical usage.

#### (iii) Modified slide mounting method B (4 steps)

For ethanol-preserved specimens.

1. Heating: specimens were placed in a watch glass filled with 10% KOH and heated for 5 mins at 85 °C.
2. Rehydrating: specimens were placed in water and left to soak for 5–10 mins. We found that heating the specimens prior to submerging them in water aided in the rehydration process.
3. Cleaning: specimens were moved to a watch glass filled with Hoyer’s medium. Because Hoyer’s medium is a self-cleaning fluid (Anderson 1954), specimens were placed in the dish to accelerate the cleaning.
4. Mounting: on a labeled slide, a drop of Hoyer’s medium was placed in the center and spread to avoid specimen drift. The specimen was placed in the Hoyer’s medium dorsoventrally and legs and antennae were positioned properly.

#### (iv) Modified slide mounting method C – balsam method with mesh container (7 steps)

For fresh specimens and ethanol-preserved specimens.

**Figure 1.**
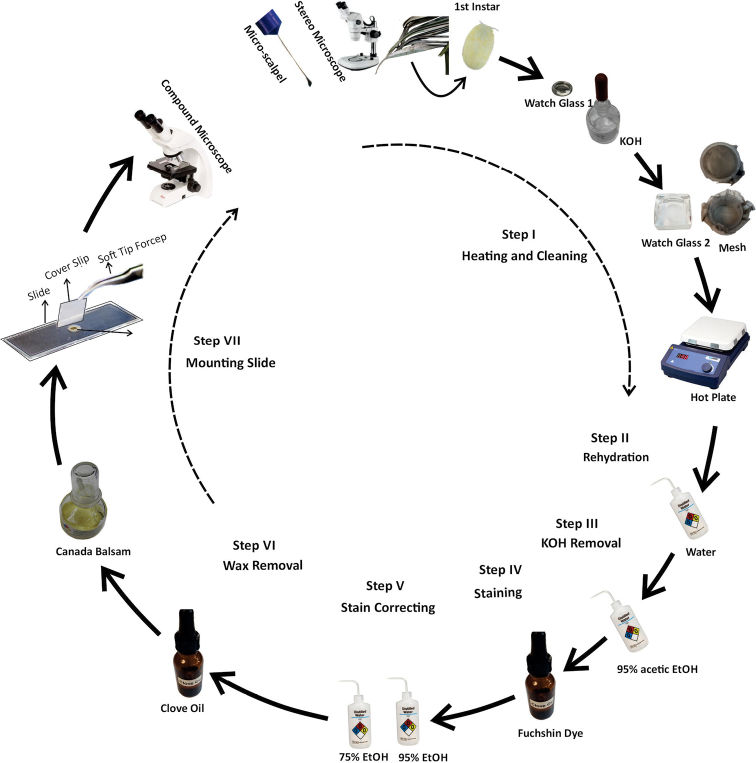
Illustration of modified slide mounting method C (balsam method with mesh container).

1. Heating and cleaning: specimens were placed in a watch glass filled with 10% KOH and heated at 85 °C for 5–10 mins. After heating, cavity contents were teased out using a micro-spatula. Once this step was completed, specimens were moved to a container modified using mesh placed in a watch glass (Fig. 1). The modified mesh container was made using a plastic 5 ml screw-top tube and fine wire mesh (Humboldt, Elgin, IL United States). The top was cut out of the screw-top tube and the mesh was put in its place, allowing liquid to move through the mesh while keeping the specimens inside.
2. Rehydration: specimens were placed in water and left to soak for 5 to 10 mins.
3. KOH removal: specimens were moved to a watch glass of 95% acetic EtOH (a few drops of glacial acetic acid with 95% ethanol) for 10 mins.
4. Staining: after removing 95% acetic EtOH, acid fuchsin stain was added and let sit for 5 mins.
5. Stain correction and dehydration: specimens were moved to a watch glass of 75% EtOH for 10 mins. Specimens were then placed in 95% EtOH for another 10 mins to dehydrate the cuticles.
6. Wax removal: specimens were soaked in clove oil for 5 to 10 mins.
7. Mounting: on a labeled slide, a drop of balsam was placed in a center and spread to avoid specimen drift. The specimen was placed in the balsam and legs and antennae were positioned properly. A coverslip was placed on the balsam and the slide was placed on a hot plate at 30 °C to remove any bubbles.

Although the mesh is effective in keeping first- and second-instar nymphs in the container without damage, a few problems were noted. The mesh does not sit flat against the glass bottom of the watch glass, so the cleaning step cannot be done in the mesh. Cleaning must be done in a watch glass and then specimens must be moved back into the mesh for the remaining steps. Due to the smaller size of the mesh container, range of motion using microtools throughout this process can be limited. Similar to processing in a watch glass without mesh, specimens can get stuck on the upper sides of the modified dish. Visibility of first-instar nymphs can be hampered by the reflective coloration of the mesh.

There are several steps involved in traditional slide-mounting protocols (method i) that require each specimen to be moved to and from at least five different watch glasses before eventually being slide mounted. Many first-instar nymphs can be lost or damaged during these steps. We recommend using the mesh container during the slide-mounting protocol (method iv). Use of this container will decrease mounting time, reduce specimen loss, decrease the quantity of chemical reagents, and generate quality slides. All steps can easily be performed using the mesh container except for the cleaning step. Unfortunately, the cleaning step must be done in a watch glass and then the specimens should be moved back into the mesh container to finish the mounting process. Although this procedure is laborious, we recommend it when the aim is to make permanent mounts for deposit in archival collections. The other mounting procedure is to place first-instar specimens directly into Hoyer’s mounting medium on a slide (method ii, iii). This protocol has fewer steps and less chance of specimen loss, and yields specimens with superior visibility. We recommend this protocol for rapid species diagnosis. Unfortunately, the mounts are only temporary unless slides are ringed to prevent deterioration.

### DNA extractions, polymerase chain reaction (PCR), and sequencing

DNA was extracted from individual *Fiorinia* and *Thysanofiorinia* specimens using the Qiagen Blood and Tissue Kit per the manufacturer’s protocol. Extractions were non-destructive, and recovery of individual scale vouchers was attempted. DNA was quantified on a Nanodrop 2000 and PCRs had a target input of at least 5 ng of genomic DNA. PCRs were performed using the Kapa HiFi HotStart PCR Kit, in a total volume of 25 uL.

The standard cytochrome oxidase I (COI) barcode region was targeted for each species using the primer pair PCOF1 (Park et al. 2010) and HCO2198/LEPR1 (Folmer et al. 1994; Hebert et al. 2004). Park et al. (2010) suggested PCOF1/LEPR1 for COI barcoding of scale insects. Some species failed to amplify with this primer combination, necessitating the alternative reverse primer HCO2198. Thermocycles were as follows: 1) initial denaturing at 95 °C for two mins, 2) 98 °C for 30 secs, 3) 50 °C for 30 secs, 4) 72 °C for 40 secs [32 cycles of steps 2–5], 5) final extension at 72 °C for seven mins, and 6) a final hold of 4 °C.

Two other loci, the large ribosomal subunit (28S D2/D3 expansion region) and elongation factor 1α (EF1α) were also targeted, for comparison with the results of Normark et al. (2019). The primer pair for EF1α was EF-1α (a) (Morse and Normark 2006) and EF2 (Palumbi 1996). The primer pair for 28S was s3660 (Dowton and Austin 1998; Morse and Normark 2006) and a335 (Whiting et al. 1997; Normark et al. 2019). Thermocycles for s3660/a335 were as follows: 1) initial denaturing at 95 °C for two mins, 2) 98 °C for 30 secs, 3) 62 °C for 30 secs, 4) 72 °C for one minute [32 cycles of steps 2–5], 5) final extension at 72 °C for seven mins, and 6) a final hold of 4 °C. Thermocycles for EF-1α(a)/EF2 were as follows: 1) initial denaturing at 95 °C for two mins, 2) 98 °C for 30 secs, 3) 64 °C for 30 secs, 4) 72 °C for 45 secs [35 cycles of steps 2–5], 5) final extension at 72 °C for seven mins, and 6) a final hold of 4 °C.

PCRs were visualized on 1.5% agarose gels. Positive PCRs were purified and prepared for sequencing using BigDye Terminator v3.1 chemistry. Amplicons were sequenced bidirectionally on the ABI SeqStudio platform at FDACS-DPI. Sequence chromatograms were trimmed and assembled in Sequencher 5.4.6. Newly generated sequences were deposited in GenBank (Suppl. material 2: Table S1) (COI: MW883907–MW883949; 28S: MW883848–MW883886; EF1α: MW893442–MW893456).

### Data analysis

Cytochrome oxidase I barcode sequences (5’-COI) were initially aligned using an online version of MAFFT 7 (Katoh and Standley 2013) with the FFT-NS-2 strategy for relatively short, similar sequences. A few sequences with excessive ambiguities or large insertions were excluded from further analysis. The resulting barcode matrix included 1177 terminal taxa and was 649 bp in length.

Sequences were aligned using the default settings of MUSCLE (Edgar 2004) and Clustal W (Larkin et al. 2007) as implemented in MEGA X (Kumar et al. 2018). The lengths of the alignments were 645bp (5’-COI), 226 bp (3’-COI), 504 bp (COII), 708 bp (EF1α, introns omitted), and 425 bp (28S, regions of uncertain homology omitted). Alignments were concatenated as a single nexus file in Mesquite 3.51 (Maddison and Maddison 2018). PCR amplifications with 3’COI/COII primers failed to produce clear bands or clean sequence data on each attempt in this study. All of 3’COI/COII sequences used in this study were from Normark et al. (2019).

Neighbor-joining and distance analyses of the 5’-COI matrix were conducted in MEGA X (Kumar et al. 2018). Neighbor-joining trees were constructed using the K2P model (Kimura 1980) with partial deletion of missing data and a site coverage cutoff of 95%. Node support was assessed using 10,000 bootstrap replicates. The resulting tree topology was adjusted in FigTree v1.4.3 (Rambaut 2012) to arrange nodes and collapse large clusters. Intra- and interspecific K2P distances among *Fiorinia* species were calculated with the same parameters as above using a separate alignment that only included *Fiorinia* barcodes.

Phylogenetic analyses were conducted using 3 sequence regions reported in Normark et al. (2019): portions of cytochrome oxidase I and II (using a 3’ portion of COI nonoverlapping with the 5’-COI barcoding matrix: 3’-COI & COII), elongation factor 1a (EF1α), and the large ribosomal subunit (28S), as well as the 5’-COI region. The aim was to assess the monophyly of *Fiorinia* and the relationship of *Fiorinia* species to other species of Fioriniina.

Maximum Likelihood (ML) phylogenetic analyses were conducted on the XSEDE computing cluster as part of the CIPRES Science Gateway (Miller et al. 2010). ML analyses were conducted using IQ-TREE version 2.0 (Minh et al. 2020). The concatenated matrix (111 terminal taxa; 2508 bp long) was partitioned by gene [27% (30/111 taxa) coverage for COI-5P: 1–645 (645 bp) + 55% (61/111) for EF1a: 646–1353 (708 bp) + 38% (42/111) for COII: 1354–1857 (504 bp) and COI-3P: 1858–2083 (226 bp) + 100% (111/111) for 28S: 2084–2508 (425 bp)] and by codon position for EF1α (2 partitions: positions 1 & 2 vs. position 3). Best fit models of sequence evolution were assessed using Bayesian Information Criteria by ModelFinder (Kalyaanamoorthy et al. 2017) in the following partition order: 5’-COI & 3’-COI (TIM+F+I+G4), EF1α positions 1 & 2 (TIM3e+I+G4), EF1α position 3 (TPM3+F+G4), COII (TN+F+I+G4) and 28S (TVM+F+I+G4). Maximum parsimony tree searches were conducted in MPBoot (Hoang et al. 2018b) with default parsimony ratchet settings.

Node support was assessed by 10,000 ultrafast ML bootstrap replicates (Hoang et al. 2018a), 10,000 SH-aLRT replicates (Guindon et al. 2010), and 1000 standard ML bootstrap replicates. Maximum parsimony (MP) support for nodes was assessed using 10,000 ultrafast bootstraps in MPBoot (Hoang et al. 2018b). Strong node support values are provided on the tree from left to right as ML standard BS (> 75), ML ultrafast BS (> 95), SH-aLRT (> 80), and MP ultrafast BS (> 95) (Guindon et al. 2010; Hoang et al. 2018a, b).

## Results

### Phylogenetic analyses

**Figure 2.**
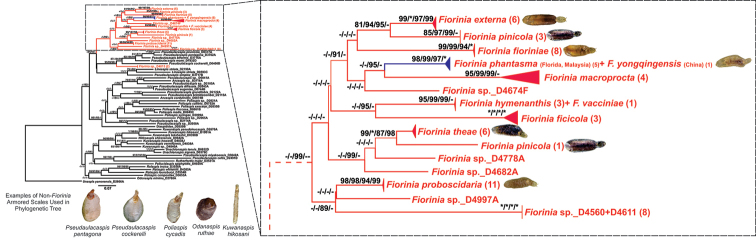
Maximum Likelihood bootstrap consensus tree of the subtribe Fioriniina based on 28S, EF1-α, 5’-COI, 3’-COI, and COII. The clade highlighted in red indicates a monophyletic *Fiorinia*. The close-up of *Fiorinia* clade is presented on right side. High-resolution figure of the main tree is in Suppl. material 1: Fig. S2.

Maximum Likelihood analyses estimated a consensus bootstrap tree with a log-likelihood of -20,889.543 for the multigene tree (Fig. 2, Suppl. material 1: Fig. S2) and -3006.382 for the 28S tree (Suppl. material 1: Fig. S3). Parsimony ratchet analyses found five equally parsimonious trees with 4123 steps. A clade of grass-feeding Fioriniina (*Unachionaspis* MacGillivray + [*Kuwanaspis* MacGillivray + *Nikkoaspis* Kuwana]) was recovered, but the node was only weakly supported (Fig. 2). As in Normark et al. (2019), the Australasian Fioriniina (*Pseudaulacaspis* MacGillivray in part; *Poliaspis* Maskell; *Anzaspis* Henderson) were recovered as a clade by likelihood and parsimony methods, but with relatively higher support in some analyses (BS 80; SH-aLRT 92). These Australasian Fioriniina were sister to a clade of *Fiorinia* + *Lineaspis* MacGillivray + *Pseudaulacaspis* in part, with weak support except for SH-aLRT (92). The clade of *Fiorinia* + *Lineaspis* + *Pseudaulacaspis* was found by likelihood and parsimony, with some strong support (ML UF BS 95; SH-aLRT 93) (Fig. 2). Relationships within this clade were not entirely resolved, resulting in a polytomy. *Fiorinia* is monophyletic in our tree, with the exception of two isolates (*Fiorinia* sp., D4815B and D4815C) which were represented only by 28S data. These two isolates belong to an undescribed *Fiorinia* species from Malaysia. The remaining *Fiorinia* isolates formed a clade in likelihood analyses (SH-aLRT 99). Relationships among *Fiorinia* species were generally weakly supported. A terminal group of *Fioriniaphantasma* + *F.yongxingensis* was present in every analysis with strong support suggesting synonymy (Fig. 2).

The slide-mounted cuticle of D4815B and other specimens in the same lot have been re-examined by BBN and they clearly belong to a pupillarial species whose morphology is completely consistent with the genus *Fiorinia*. These results might imply that the lineage leading to D4815B and D4815C represents a second origin of the pupillarial habit in Fioriniina. These two isolates were placed within a section of a *Fiorinia* + *Rolaspis* + *Pseudaulacaspis* (in part) clade in the ML phylogenetic tree using only 28S data (Suppl. material 1: Fig. S3). They were placed with five species of *Pseudaulacaspis* (including *P.biformis*, *P.cockerelli*, *P.momi*, *P.pentagona*, and *P.prunicola*) with strong support (Suppl. material 1: Fig. S3). In addition to these five species of *Pseudaulacaspis*, three species of *Rolaspis* (including *R.incisa*, *R.lounsburyi*, and *R.whitehilli*), and one species of *Pellucidaspis* (*P.epiphytidis*) were also placed within this *Fiorinia* clade.

### COI barcoding

**Figure 3.**
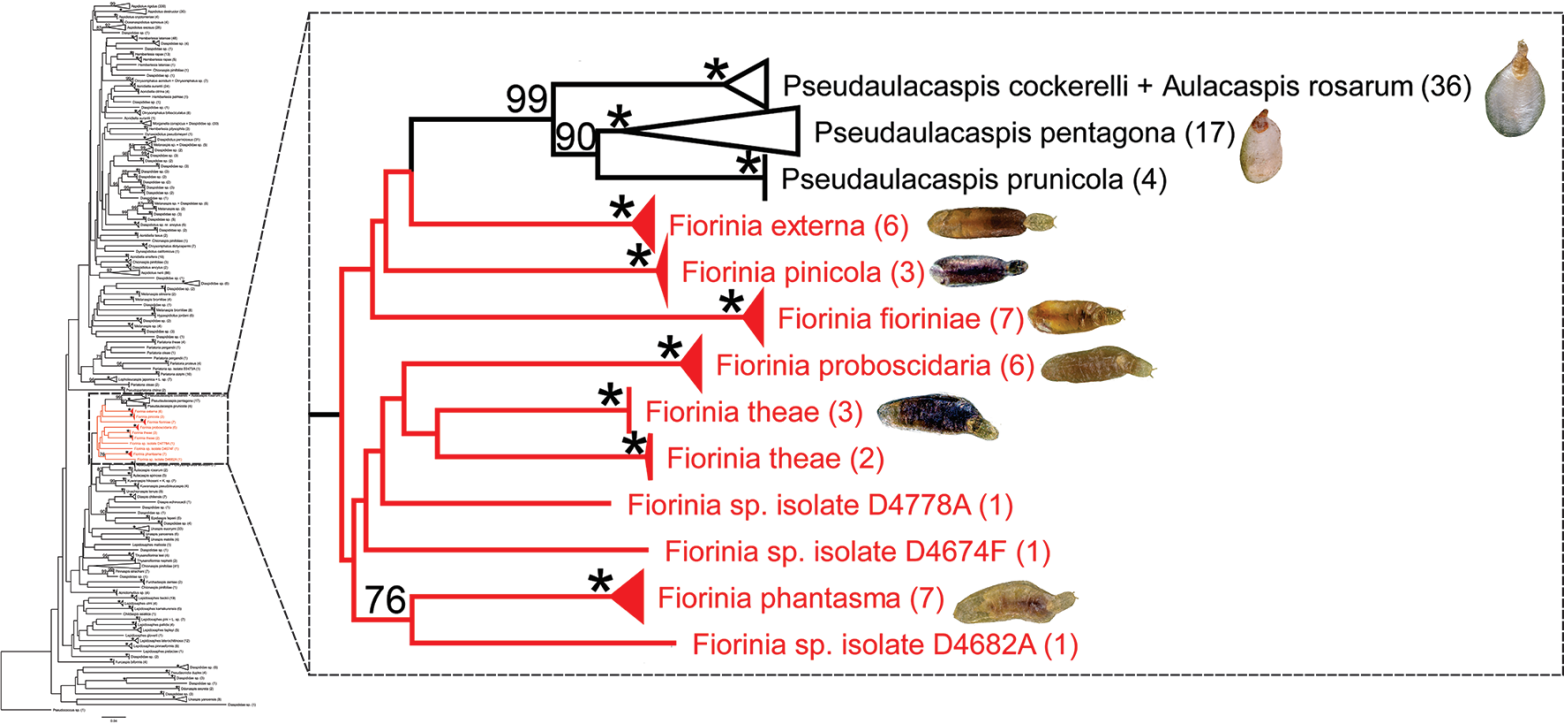
Neighbor-joining tree of Diaspididae 5’-COI barcodes. Terminal taxa are labeled to their narrowest identification-level. Numbers in parentheses after terminal taxa indicate how many sequences are represented in each cluster. The cluster of *Fiorinia* species is highlighted in red. Bootstrap support values greater than 75 are indicated on the tree. Nodes with 100 percent bootstrap support are indicated by a “*”. The close-up of the *Fiorinia* clade is presented on the right side. High-resolution figure of the main tree is in Suppl. material 1: Fig. S4.

This study produced 43 new sequences of the COI barcode region, 37 of which represent nine *Fiorinia* species (Fig. 3, Suppl. material 1: Fig. S4). The remaining 6 COI barcode sequences represent two species of *Thysanofiorinia*. These new barcode sequences range in length 562 bp–645 bp. In the neighbor-joining analyses of DiaspididaeCOI barcodes, *Fiorinia* species cluster near the species of *Kuwanaspis*, *Unachionaspis*, and *Pseudaulacaspis* (all members of Fioriniina), along with a sequence assigned to the genus *Aulacaspis* (subtribe Chionaspidina). (Fig. 3). *Fiorinia* species represented by multiple barcode sequences each formed well-supported clusters (100 BS) in the neighbor-joining tree, with one exception: *F.theae*. *Fioriniatheae* forms two well supported clusters whose relationship to each other is not resolved in this analysis (Fig. 3).

The alignment for calculating K2P distances among *Fiorinia* species included 37 terminal taxa and was 645 bp long. Based on the 95% site cutoff, calculations involved 560 total positions. Intraspecific K2P distances were low, except for specimens identified as *F.theae* (Table 1). Interspecific K2P distances between *Fiorinia* species ranged from 9.1% to 15.2% (Table 1). Sequences of *F.phantasma* from the population from Florida and Malaysia and sequences of *F.yongxingensis* were identical and were placed together in the tree with strong support (Fig. 3).

**Table 1.** Summary of *Fiorinia*COI barcode intra- and interspecific K2P distances.

Species	Intra. K2P Dist.	Inter. K2P Dist.
*Fioriniaexterna* (n = 6)	0.00%	9.1%–12.4%
*Fioriniafioriniae* (n = 7)	0.00%–0.02%	11.8%–14.8%
*Fioriniaphantasma* (n = 7)	0.00%–0.09%	9.1%–13.7%
*Fioriniapinicola* (n = 3)	0.00%	9.1%–15.2%
*Fioriniaproboscidaria* (n = 6)	0.00%–0.02%	9.9%–14.2%
*Fioriniatheae* (n = 5)	0.00%–8.00%	9.5%–14.8%
*Fiorinia* sp. isolate D4778A (n = 1)	N/A	9.5%–13.9%
*Fiorinia* sp. isolate D4674F (n = 1)	N/A	9.5%–12.7%
*Fiorinia* sp. isolate D4682A (n = 1)	N/A	9.1%–15.2%

### General descriptions of second-instar nymphs of *Fiorinia* species occurring in the USA

#### Second-instar females

With two definite pairs of lobes; third lobes and sometimes fourth lobes represented by series of points. Median lobes yoked, medial margins divergent or nearly parallel, longer than lateral margin, with series of notches. Second lobes bilobed, usually smaller than median lobes, sometimes wider, medial lobule largest, sometimes with small notches, lateral lobule sometimes with one or two small notches. Third lobes usually represented by raised sclerotized area with small series of notches, often divided into two lobules by seta marking segment VI. Fourth lobes sometimes represented by series of sclerotized points. Gland spine arrangement of two types: *F.proboscidaria* and *F.theae* with single gland spine on each side of each of segments II–VIII, gland spines on each side of segments II–IV larger than those on segments V–VIII, without gland spines on segment I; remaining species with single gland spine on each side of segments II–V, absent from segment VI, present on each side of segments VII and VIII, gland spines on each side of segments II–V larger than those on segments VI–VIII, with two or three smaller gland spines on each side of segment I; gland spines with barely perceptible sclerotization posterolaterad of each spiracle. Macroducts barrel shaped, marginal, with four or five on each side of pygidium from segments III or IV–VII. Microducts restricted to venter, three different patterns on abdomen; in *F.proboscidaria* and *F.theae* longitudinal lines on each side of abdomen from II–VI, each line composed of one or more ducts on each segment, mediolateral line on segments III or IV, V or VI, submarginal line on segments II–VI; in *F.externa*, *F.fioriniae*, *F.japonica*, and *F.pinicola* longitudinal lines on each side of abdomen from II–VI, each line composed of one or rarely two ducts on each side of each segment, mediolateral line on segments II–V or VI, submarginal line on segments II–VI; in *F.phantasma* longitudinal lines restricted to mediolateral areas of segments II–IV or V, other lines absent; microducts on head and thorax usually anterior of clypeus, laterad of labium, and posterior of each spiracle. Perispiracular pores associated with anterior spiracles only, with three loculi, one or two pores associated with each spiracle. Anal opening normally located in center of pygidium mesad of fourth marginal macroduct counting forward from posterior macroduct. Dorsal setae present near body margin on head and thorax, with one seta submarginally on each side of each abdominal segment; also present in mediolateral area on each side of body on any or all of abdominal segments I–VI; usually with one mediolateral seta on each side of head. Ventral setae in small numbers in marginal areas of head and thorax, with one seta usually present laterad of each spiracle; abdominal segments with one marginal and one submarginal seta on each side of each segment and with one mediolateral seta on each side of segments IV–VI. Antennae each normally with one long seta and two small sensillae. Cicatrices present or absent on each side of abdominal segment I. Two inconspicuous lobes present submarginally on head of *F.proboscidaria* and *F.theae*.

##### Notes

Characters most useful in distinguishing among species are: a) number of marginal macroducts; b) arrangement of gland spines; c) arrangement of microducts; d) presence or absence of cicatrices; e) presence or absence of lobes on head; f) relative size of median lobes compared to medial lobule of second lobe; g) shape of median lobes.

Second-instar females of *Fiorinia* species can be distinguished from most similar genera by having the following: median lobes yoked, usually divergent, medial margin longer than lateral, with one pair of setae between; dorsal macroducts confined to body margin, with four or five on each side of pygidium; with two pairs of definite lobes, second pair bilobular. However, we have been unable to distinguish between second-instar females of the *Fiorinia* species treated here and *Pseudaulacaspiscockerelli* (Comstock) and *P.pentagona* (Targioni Tozzetti). There are consistent differences in the distribution of the gland spines in most species of *Fiorinia*, but *F.proboscidaria* and *F.theae* are identical to *P.cockerelli* and *P.pentagona*. It is remarkable that the second-instars are so similar, but the adult females are quite different.

#### Second-instar males

With two definite pairs of lobes; remaining body margin often with numerous projections, not organized into clear lobes. Median lobes spaced apart, without zygosis, usually with small medial lobule and large, conspicuous lateral lobule, medial lobule with one or two projections, lateral margin with several notches and projections. Second lobes usually associated with a dense cluster of marginal ducts, with series of projections, rarely bilobed, smaller than median lobes. Gland spines of three sizes: largest in clusters posterolaterad of each anterior spiracle, posterolaterad of posterior spiracle, and submarginal on abdominal segment I and sometimes II; medium-sized gland spines on body margin of anterior abdominal segments; small gland spines laterad of anterior spiracle on *F.externa* and *F.theae*. Macroducts barrel shaped, of two sizes: larger ducts grouped into communal ducts (= glanduliferous craters; Takagi 1999) that exit through single orifice with numerous fine filaments or series of short projections on margin; communal ducts either separate or associated with clusters of smaller macroducts; smaller macroducts ca. half as large as larger ducts, arranged singly or in clusters on prepygidial and pygidial margin. Microducts present on dorsum and venter, arranged in longitudinal lines, of two sizes: smaller size relatively slender, longer than wide, present on venter of most abdominal segments, on venter of head, in ventromedial areas of thorax, and on dorsum of posterior two or three segments; larger ducts ca. as long as wide present on venter in submarginal areas of thorax, on dorsum in submarginal areas of prothorax to anterior abdominal segments and submedially on anterior abdominal segments. Perispiracular pores associated with anterior spiracles only, with three loculi, 1–3 pores associated with each spiracle. Anal opening normally located in center of pygidium mesad of anterior edge of posterior cluster of macroducts. Dorsal setae present near body margin on head and thorax, setae associated with duct clusters long and conspicuous; also present in mediolateral area on each side of body on any or all of abdominal segments I–VI, usually with several mediolateral seta on each side of head. Ventral setae in small numbers in marginal areas of head and thorax, with one seta usually present laterad of each spiracle; abdominal segments with one marginal and one submarginal seta on each side of each segment and with one mediolateral seta on each side of segments IV–VII. Antennae each normally with one long seta and two small sensillae. Cicatrices absent.

##### Notes

Characters most useful in distinguishing among species are: a) arrangement and number of communal ducts b) organization of duct clusters c) arrangement of microducts; d) arrangement of gland spines. Second-instar males of *Fiorinia* are remarkably similar to the same instar of *Pseudaulacaspis* species by each having unusual lobes, duct clusters, and communal ducts (Takagi and Kawai 1967). *Pseudaulacaspis* species differ primarily by the presence of many ventral microducts on the head and barrel-shaped microducts in the medial and submedial areas of the abdominal venter, whereas *Fiornia* species possess no more than two ventral microducts on the head, and slender microducts on the submedial areas of the abdominal venter. In Normark et al. (2019), the subtribe Fioriniina comprises many genera and species with second-instar males that are similar in appearance to the species treated here.

#### First-instar nymphs

Howell (1977) gave a general description of the first-instar nymphs of the species that he examined. We will not repeat that here. Below, we present diagnoses of the two species that were not included in the Howell (1977), i.e., *F.phantasma* and *F.proboscidaria*.

First-instar nymphs of *Fiorinia* species can be recognized by having the following combination of characters: antennae five segmented; apical segment annulate; large duct on each side of dorsum of head; submedial longitudinal line of microducts on each side of thorax; second lobes bilobulate. First-instar nymphs of *Fiornia* species are similar to some species of *Pseudaulacaspis* (*P.cockerelli* and *P.pentagona*) but differ by normally having a submedial longitudinal line of microducts on each side of thorax, whereas these ducts are absent from *P.cockerelli* and *P.pentagona* (Tippins and Howell 1983).

### Keys to *Fiorinia* species occurring in the USA using immature instars

#### First-instar nymphs (adapted from Howell 1977).

**Table:** 

1	Lobules of pygidial lobe 2 rounded	**2**
–	Lobules of pygidial lobe 2 truncate	***F.externa***
2	Gland spines on segment VI more than ½ length of gland spine on segment VII	**3**
–	Gland spines on segment VI less than ½ length of gland spine on segment VII	**5**
3	Sclerotized pattern outside of oval surrounding large duct on dorsum of head mostly thin and serpentine like; large duct short, broad, with inner apex nearly flat	**4**
–	Sclerotized pattern outside of oval surrounding large duct on dorsum of head mostly in clumps; large duct elongate, narrow, with inner apex mushroom shaped	***F.phantasma* (Fig. 10)**
4	Gland spine on segment VI ca. ½ length of gland spine on segment VII	***F.fioriniae***
–	Gland spine on segment VI nearly equal to length of gland spine of segment VII	***F.proboscidaria* (Fig. 16)**
5	Dorsal submedian thoracic ducts present; inner apex of large duct on dorsum of head flat	**6**
–	Dorsal submedian thoracic ducts absent (occasionally orifices present); inner apex of large duct on dorsum of head mushroom shaped	***F.japonica***
6	Gland spine on segment VI noticeably longer than those on segments I–V; pattern of dorsal derm on abdomen fine	***F.pinicola***
–	Gland spine on segment VI equal to those on segments I–V; pattern of dorsal derm on abdomen coarse	***F.theae***

#### Second-instar females

**Table:** 

1	With 5 pairs of marginal macroducts	**2**
–	With 4 pairs of marginal macroducts	***F.theae* (Fig. 20), *F.proboscidaria* (Fig. 18)**
2	With submarginal longitudinal line of microducts on venter; with 4 large-sized gland spines on each side of body; without deep incision anterior of 5^th^ macroduct (segment III) on older specimens	**3**
–	Without submarginal longitudinal line of microducts on venter; with 3 large-sized gland spines on each side of body; with deep incision anterior of 5^th^ macroduct (segment III) on older specimens	***F.phantasma* (Fig. 12)**
3	Median lobes broad, as wide as or wider than medial lobule of second lobe	4
–	Median lobes narrow, narrower than medial lobule of second lobe	***F.externa* (Fig. 4)**
4	With 3 pairs of microducts on head; space between bases of median lobes wider than medial lobule of second lobes	***F.fioriniae* (Fig. 6)**
–	With 1 pair of microducts on head; space between bases of median lobes equal to or narrower than medial lobule of second lobes	***F.japonica* (Fig. 8), *F.pinicola* (Fig. 14)**

#### Second-instar males

**Table:** 

1	One or 2 duct clusters on each side of body, or definitive clusters absent	**2**
–	Three duct clusters on each side of body	***F.externa* (Fig. 5)**
2	Communal ducts present; small macroducts on pygidial margin in at least 1 cluster	**3**
–	Communal ducts absent; small macroducts on pygidial margin not in tight cluster	***F.fioriniae* (Fig. 7)**
3	Communal ducts incorporated in cluster of small macroducts	**5**
–	Communal ducts separate, not in cluster with small macroducts	**4**
4	With 1 communal duct on each side of pygidium	***F.proboscidaria* (Fig. 19)**
–	With 2 communal ducts on each side of pygidium	***F.theae* (Fig. 21)**
5	With 1 communal duct on each side of body	***F.phantasma* (Fig. 13)**
–	With 2 communal ducts on each side of body	**6**
6	With 5 or more gland spines on each side of body between anterior and posterior spiracles	***F.pinicola* (Fig. 15)**
–	With fewer than 5 gland spines on each side of body between anterior and posterior spiracles	***F.japonica* (Fig. 9)**

#### Adult Females (adapted from Watson et al. (2015)

**Table:** 

1	Interantennal process absent	**2**
–	Interantennal process present	**5**
2	Antennae each with a long spur making them longer than wide	3
–	Antennae each with a short spur making them more or less as long as wide	***F.externa***
3	Fewer than 7 marginal macroducts on each side of pygidium	**4**
–	Seven or 8 marginal macroducts on each side of pygidium	***F.pinicola***
4	Four to 6 (normally 5) marginal macroducts on each side of pygidium; clusters of ventral microducts near body margins of abdominal segments III and IV	***F.japonica***
–	Three or 4 (normally 3) marginal macroducts on each side of pygidium; clusters of ventral microducts absent near body margins of abdominal segments III and IV	***F.fioriniae***
5	Interantennal process without spicules; body narrow, with almost parallel sides	**6**
–	Interantennal process with spicules; body wide, narrowing abruptly to triangular pygidium	***F.phantasma* (Fig. 11)**
6	Seven or 8 marginal macroducts on each side of body; head rounded	***F.theae***
–	Three to 5 marginal macroducts on each side of body; head conical	***F.proboscidaria* (Fig. 17)**

### Species accounts

**Figure 4.**
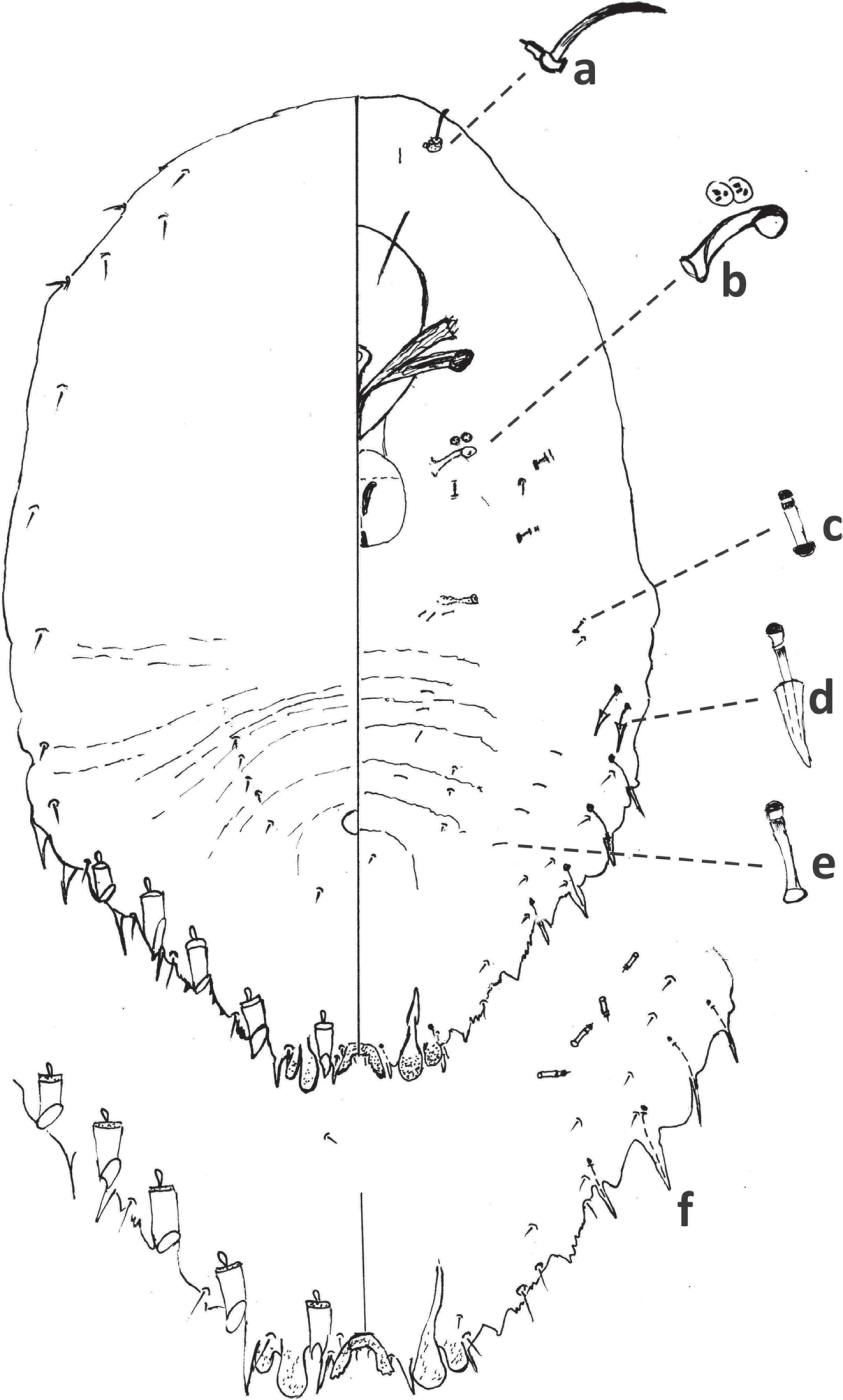
*Fioriniaexterna*, second-instar female, Alleghany Co., Glade Creek, North Carolina, November 22, 2019, on *Abiesfraseri*, A. Bartlett, (2019-6449). Abbreviations: a) antenna; b) anterior spiracle; c) microduct with sclerotized orifice; d) large gland spine; e) small microduct; f) enlargement of pygidium.

#### Fiorinia externa

Taxon classificationAnimaliaHemipteraDiaspididae

Ferris, 1942

4744B773-C87C-52D2-9755-F401DBFD1E7D

##### Field characteristics.

First-instar exuviae barely touching second-instar exuviae. Distinct indentation formed between attachment of first- and second-instar exuviae. Second-instar exuviae narrow, parallel sided, and elongate; longitudinal ridge absent or weakly developed. Second-instar exuviae reddish brown anteriorly and light brown to yellow posteriorly. Posterior end of adult female within second-instar exuviae rounded (Suppl. material 1: Fig. S1).

##### First-instar nymph.

Described in Howell (1977).

##### Second-instar female.

Median lobes slender, narrower than medial lobule of second lobe, not projecting beyond medial lobule of second lobes. With five pairs of marginal macroducts. Swelling of body margin adjacent to macroduct usually pointed. With four large gland spines on margin of each side of body from abdominal segments II–V; usually without small gland spine on each side of abdominal segment VI; with small gland spines on margin or submargin of abdominal segment I. With one microduct on each side of head. Longitudinal line of microducts present submarginally on venter of II–V, normally with one microduct on each side of each segment. Cicatrices absent.

##### Second-instar male.

Three duct clusters on each side of body; posterior cluster composed of several small ducts and two communal ducts. Five longitudinal lines of microducts on venter of abdomen (one medial, two mediolateral, two submarginal). Cluster of small microducts with sclerotized orifice laterad of anterior spiracle. Fewer than five large-sized gland spines on each side of body between anterior and posterior spiracles. Antennae each with one enlarged seta.

##### Florida collection records.

All records are on Christmas trees imported from states outside of Florida. This species is not established in Florida, and its common host, *Abiesfraseri*, also does not occur naturally in Florida. It has been found on imported Christmas trees in the following localities in Florida: Broward Co., Miramar, November 20, 2013, on *Abiesfraseri*, S. Alspach (2013-8494); Broward Co., Davie, December 10, 2013, on *Abiesfraseri*, S. Beidler (2013-8906); Citrus Co., Inverness, December 4, 2013, on *Abiesfraseri*, S. Jenner (2013-9766); Hamilton Co., White Springs, December 11, 2012, on *Abiesfraseri*, H. Randolphs (2012-9239); Hillsborough Co., Tampa, November 20, 2012, on *Abiesfraseri*, T. Streeter (2012-8844); Marion Co., Ocala, December 2, 2013, on *Abiesfraseri*, S. Wayte (2013-8755); Monroe Co., Tavernier, November 28, 2012, on *Abiesfraseri*, J. Farnum (2012-8924); Volusia Co., Port Orange, November 27, 2017, on *Abiesfraseri*, K. Coffey (2017-4496).

**Figure 5.**
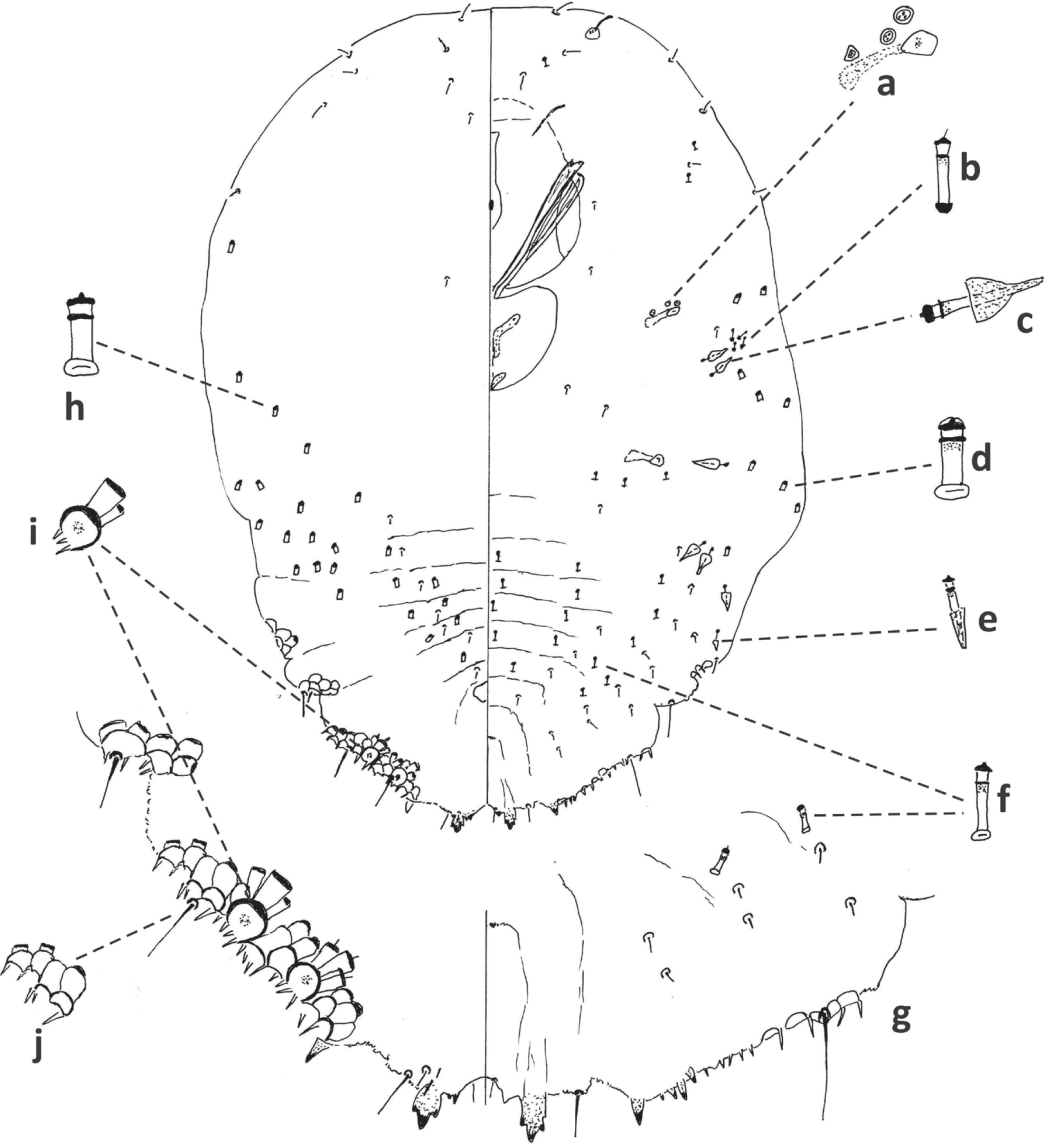
*Fioriniaexterna*, second-instar male, Alleghany Co., Glade Creek, North Carolina, November 22, 2019 on *Abiesfraseri*, A. Bartlett, (2019-6449). Abbreviations: a) anterior spiracle; b) microduct with sclerotized orifice; c) large microduct; d) large gland spine; e) small gland spine; f) small microduct; g) enlargement of pygidium; h) large microduct; i) enlargement of communal duct; j) enlargement of portion of duct cluster.

**Figure 6.**
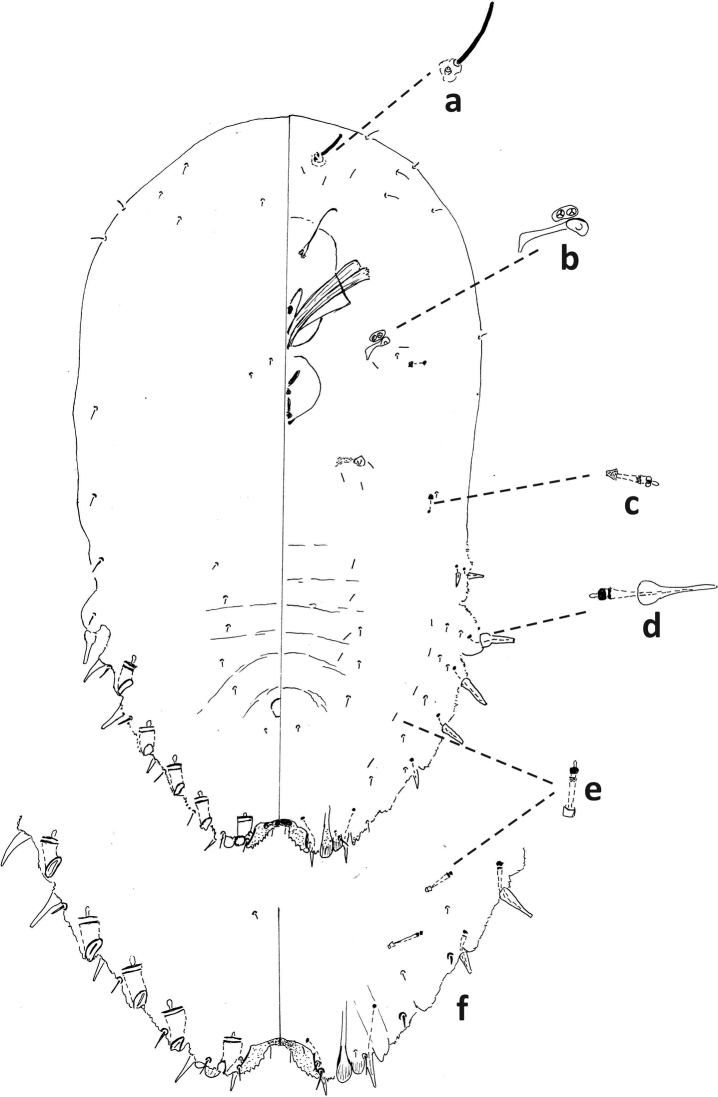
*Fioriniafioriniae*, second-instar female, Marion Co., Ocala, August 13, 2019 on *Chamaeropshumilis*, T. Gordon, (2019-4546). Abbreviations: a) antenna; b) anterior spiracle; c) microduct with sclerotized orifice; d) large gland spine; e) large microduct; f) enlargement of pygidium.

##### Specimens examined for description and diagnosis.

Alleghany Co., Glade Creek, North Carolina, November 22, 2019 on *Abiesfraseri*, A. Bartlett, 5 2^nd^ ♀, 5 2^nd^ ♂, 10 ad ♀ (2019-6449), Alleghany Co., Laurel Springs, North Carolina, December 8, 2020 on *Abiesfraseri*, L. Milton, 10 ad ♀ (2020-4778).

##### Other specimens examined from USNM.

Japan, Kobe, Arboretum, May 8, 2006, on Tsuga?sieboldii, S. Lyon 7 2^nd^ ♀ (0606537). United States, Connecticut, Danbury, September 7, 1944, on hemlock, S.W. Bromley 3 1^st^ ♀ (JOH 07-77); Connecticut, Fairfield Co., New Canaan, November 3, 1950, on Nordman fir, S.W. Bromley 1 1^st^ ♀, 18 2^nd^ ♀, 20 ad ♀. New York, Nassau Co., Oyster Bay, May 17, 1947, on hemlock, B.F. Maker 2 1^st^ ♂ (JOH 10-77); New York, Suffolk Co., Brookhaven, November 25, 1985, on leaves of hemlock, T. Kowalsick (ek-01-86); Pennsylvania, Radnor, July 26, 1946, on hemlock, S.W. Bromley 1 1^st^ ♀ (JOH 08-77).

#### Fiorinia fioriniae

Taxon classificationAnimaliaHemipteraDiaspididae

Targioni Tozzetti, 1867

31442326-FF12-5C51-95C6-EDF04CAD90C5

##### Field characteristics.

First-instar exuviae overlapping second-instar exuviae. Without indentation formed between attachment of first- and second-instar exuviae. Second-instar exuviae oval, convex marginally; yellow to light brown; longitudinal ridge conspicuous. Posterior end of adult female within second-instar exuviae rounded. Heavily infested leaves with slight white secretion.

##### First-instar nymph.

Described in Howell (1977).

##### Second-instar female.

Median lobes broad, equal to or wider than medial lobule of second lobe, projecting ca. same or slightly less than medial lobule of second lobes. With five pairs of marginal macroducts. Swelling of body margin adjacent to macroduct usually rounded. With four large gland spines on margin of each side of body from abdominal segments II–V; usually without small gland spine on each side of abdominal segment VI; with small gland spines on margin or submargin of abdominal segment I. With three microducts on each side of head. Longitudinal line of microducts present submarginally on venter of abdominal segments II–VI, normally with one microduct on each side of each segment. Cicatrices absent.

##### Second-instar male.

Submargin of abdominal segments II–VI with scattered small-sized macroducts, not in clusters; communal ducts absent. Medial longitudinal line of microducts absent. Cluster of small microducts with sclerotized orifice laterad of anterior spiracle absent. Fewer than five gland spines on each side of body between anterior and posterior spiracles. Antennae each with several enlarged setae.

##### Notes.

The single specimen collected with identified adult females of this species is unusual and may not be the second-instar male of this species. U.S. populations of *Fioriniafioriniae* have been reported to be parthenogenetic (Tippins 1970), so it is surprising to find a male, although many scale insect species with parthenogenetic populations also have sexual populations (Nur 1990). The specimen is unusual among second-instar males of *Fiorinia* in lacking tight clusters of marginal ducts. There exist a few other species of *Fiorinia* with males that similarly lack these ducts, for instance *F.nachiensis* Takahashi of Japan; thus it is plausible that this really is the male of *F.fioriniae*.

##### Florida collection records.

Brevard Co., Melbourne, February 22, 1984, on *Phoradendron* sp., F.A. Smith (1984-2933, 3014) (2 slides); Brevard Co., Sharpes, January 19, 1972, on *Callistemon* sp., H.C. Levan (1972-005–008) (4 slides); Broward Co., Dania, January 4, 1966, on *Howea* sp., J.W. Shirah (1966-7369); Broward Co., Dania, June 26, 1981, on *Mangiferaindica*, M. McDonald (1981-1606) (2 slides); Broward Co., Dania, August 24, 2011, on *Persea* sp., G. Azone (2011-5990); Broward Co., Davie, April 3, 1962, on *Ilex* sp., D.P. McLean (1962-2896) (2 slides); Broward Co., Davie, October 12, 1978, on *Camellia* sp., R. Gaskalla (1978-2879) (2 slides); Broward Co., Coral Springs, October 5, 2011, on *Perseaamericana*, L. Charlton (2011-7789) (2 slides); Broward Co., Fort Lauderdale, February 5, 1968, on *Callistemon* sp., D.C. Clinton (1968-2883) (2 slides); Broward Co., Fort Lauderdale, February 6, 1970, on *Callistemon* sp., D.C. Clinton (1970-2878) (2 slides); Broward Co., Fort Lauderdale, May 2, 1974, on *Sabal* sp., J.A. Reinert (1974-2915) (2 slides); Broward Co., Fort Lauderdale, November 13, 1979, on *Callistemonciternus*, K. Tyson (1979-7500) (2 slides); Broward Co., Fort Lauderdale, February 25, 1988, on *Howeaforsteriana*, J. McCluskie (1985-2925) (2 slides); Broward Co., Fort Lauderdale, February 25, 1988, on *Manilkararoxburghiana*, J. Hickey (1988-005); Broward Co., Fort Lauderdale, January 8, 1984, on *Manilkararoxburghiana*, J. Hickey (1984-3287) (3 slides); Broward Co., Fort Lauderdale, December 26, 2003, on *Leucospermum* sp., G. Farina (2003-6693); Broward Co., Fort Lauderdale, June 4, 2004, on *Laurusnobilis*, F.W. Howard (2004-4142) (3 slides); Broward Co., Hollywood, February 19, 1979, on *Perseaamericana*, R. Gaskalla (1978-3264) (2 slides); Broward Co., Hollywood, June 1986, on *Howeaforsteriana*, D. Fenster (1986-008) (3 slides); Broward Co., Hollywood, December 5, 1997, on *Ravenearivularis*, M.S. Quintanilla (1997-2912) (3 slides); Broward Co., North Lauderdale, May 28, 1981, on *Dicytospermaalbum*, D. Clinton and J. Aubry (1981-2895, 2904) (2 slides); Broward Co., Tamarac, March 21, 2012, on *Perseaamericana*, C. Millan (2012-1986); Broward Co., on unknown host, June 4, 2004, on *Laurusnobilis*, F.W. Howard (2004-4142-301); Charlotte Co., Punta Gorda, August 9, 2007, on *Camelliajaponica*, D. Renz (2007-5759); Collier Co., Naples, August 28, 2013, on Palmae, R. Nanneman (2013-6323); Duval Co., Nocatee, April 4, 1978, on *Perseaamericana*, L.J. Chambliss (1978-3288) (2 slides); Escambia Co., Pensacola, November 3, 1988, on *Prunusangustifolia*, G. Corbitt and R. Burns (1988-2899); Glades Co., Moore Haven, October 4, 2006, on *Celtislaevigata*, L. Richards (2006-7213); Hendry Co., Devils Garden, November 20, 2014, on *Perseapalustris*, M. Terrell (2014-788) (2 slides); Highlands Co., April 28, 1975, on *Camellia* sp., R.F. Denno, J.A. Davidson, D.R. Miller (1975-2886); Highlands Co., on unknown host, July 24, 1987 on *Perseaborbonia*, R. Payne (1987-2988) (2 slides); Highlands Co., Lake Placid, November 1970, on *Camellia* sp. J.A. Weidhaas (1970-3265, 3854) (2 slides); Hillsborough Co., Sun City, November 14, 1994, on *Phoradendronleucarpum*, M. Runnals (1994-2918, 3924) (2 slides); Hillsborough Co., Tampa, April 10, 1964, on *Sabal* sp., S.A. Fuller (1964-2901); Hillsborough Co., Tampa, March 25, 1983, on *Hedera* sp., E.R. Simmons (1983-2998, 3943) (2 slides); Miami-Dade Co., Homestead, June 9, 1979, on *Manilkararoxburghiana*, P. Chobrda (1979-011); Indian River Co., Vero Beach, December 16, 1970, on *Callistemon* sp., R.H. Kendrick (1970-012–016) (5 slides); Lake Co., Tavares, September 9, 2012, on *Hedera* sp., M. Sellers (2012-6901); Lee Co., Sanibel Island, April 4, 1978, on *Zamia* sp., R. Driggers (1978-2936); Leon Co., Tallahassee, February 3, 1916, on *Camellia* sp. A.C.M. (1916-017); Leon Co., Tallahassee, February 3, 1916, on *Camellia* sp., A.C.M. (1916-2850) (4 slides); Leon Co., Tallahassee, October 31, 1919, on *Camellia* sp., P.F. Robertson (1919-021); Levy Co., Bronson, January 4, 2011, on *Camellia* sp., W. Bailey (2011-29); Lucie Co., Fort Pierce, January 16, 1980, on *Dracaena* sp., E.W. Campbell (1980-2944); Lucie Co., Lakewood Park, July 16, 1980, on *Acoelorrhaphewrightii*, E.W. Campbell (1980-483, 2937) (2 slides); Madison Co., Greenville, March 12, 1993, *Ilex* sp., F. Bennett (1993-2903); Manatee Co., Oneco, December 17, 1987, on *Callistemon* sp., A. Waters (1987-014); Manatee Co., Snead Island, April 3, 1991, on *Schinus* sp., Runnals M. (1991-2922); Manatee Co., Snead Island, April 3, 1991, on *Schinus* sp. Runnals M. (1991-006–007) (2 slides); Marion Co., Weirsdale, December 22, 1985, on *Hederacanarensis*, F.J. McHenry (1985-2832, 2894) (2 slides); Martin Co., Hobe Sound, April 21, 1980, on *Perseaamericana*, E.W. Campbell (1980-2959); Martin Co., Hobe Sound, June 9, 1981, on *Callistemonviminalis*, S. Hakala (1981-418); Martin Co., Jensen Beach, September 27, 1978, on *Dictyosperma* sp., E.W. Campbell (1978-7502) (2 slides); Martin Co., Palm City, February 8, 2012, on *Magnoliavirginiana*, L. West (2012-833) (2 slides); Martin Co., Palm City, October 17, 2012, on *Perseapalustris*, L. West (2012-7964) (2 slides); Martin Co., Palm City, September 1, 1977, on *Magnolia* sp., E.W. Campbell (1977-2890, 2884) (2 slides); Martin Co., Stuart, January 31, 1978, on *Eugenia* sp., E.W. Campbell (1978-0955, 3283) (2 slides); Martin Co., Stuart, November 17, 1978, on *Myrica* sp., E.W. Campbell (1978-021); Miami-Dade Co., Big Cypress National Preserve, February 16, 1978, on *Magnolia* sp., A. Harmon and D. Martinelli (1978-2910); Miami-Dade Co., Big Cypress National Preserve, February 16, 1978, on *Magnoliavirginiana*, A. Hamon and D. Martinelli (1978-006–007) (2 slides); Miami-Dade Co., Coral Gables, August 13, 2010, on *Gymnantheslucida*, K. Griffiths (2010-4926); Miami-Dade Co., Florida City, November 19, 1986, on *Mimusopsroxburghiana*, L.D. Howerton (1986-962); Miami-Dade Co., Florida City, November 19, 1986, on *Mimusopsroxburghiana*, L.D. Howerton (1986-2914, 2980) (2 slides); Miami-Dade Co., Hialeah, March 28, 1979, on *Callistemonviminalis*, D. Stocks (1979-3382, 3869) (2 slides); Miami-Dade Co., Hialeah, January 1, 1980, on *Callistemon* sp., D. Stocks and W. James (1980-2952); Miami-Dade Co., Homestead, January 24, 1962, on *Macadamia* sp., R.J. McMillan (1962-3276) (2 slides); Miami-Dade Co., Homestead, October 16, 1962, on *Melaleuca* sp., J.H. Knowles (1962-2897) (2 slides); Miami-Dade Co., Homestead, February 2, 1969, on *Persea* sp., D.O. Wolfenbarger (1969-491) (2 slides); Miami-Dade Co., Homestead, March 28, 1969, on *Persea* sp. D.O. Wolfenbarger (1969-026–031) (4 slides); Miami-Dade Co., Homestead, February 2, 1978, on *Hedera* sp., W.E. Wyles (1978-488); Miami-Dade Co., Homestead, February 27, 1978, on *Hedera* sp., W.E. Wyles (1978-7522) (5 slides); Miami-Dade Co., Homestead, June 8, 1979, on *Manilkararoxburghiana*, P. Chobrda (1979-0146) (4 slides); Miami-Dade Co., Homestead October 3, 1979, on *Perseaamericana*, W.E. Wyles (1979-2928); Miami-Dade Co., Homestead, September 11, 2007, on *Perseaamericana*, B. Saunders (2007-6958); Miami-Dade Co., Homestead, July 31, 2018, on *Gymnantheslucida*, W. Mazuk (2018-4092) (2 slides); Miami-Dade Co., Kendall, February 24, 1989, on *Camellia* sp., W. Francillon (1989-2855) (2 slides); Miami-Dade Co., Miami, April 22, 1966, on *Chamaedora* sp., C.F. Dowling (1966-2876) (2 slides); Miami-Dade Co., Miami, June 6, 1967, on *Chamaerops* sp., J.S. Sloan (1967-2907) (2 slides); Miami-Dade Co., Miami, October 26, 1967 on *Callistemonviminalis*, J.F. Dillon (1967-3305) (2 slides); Miami-Dade Co., Miami, October 26, 1967, on *Callistemonviminalis*, J.F. Dillon (1967-038); Miami-Dade Co., Miami, March 3, 1969, on *Howea* sp., J.F. Dillon (1969-493, 3060) (2 slides); Miami-Dade Co., Miami, March 7, 1969, on *Howea* sp., J.F. Dillon (1969-3267) (4 slides); Miami-Dade Co., Miami, September 5, 1969, on *Macadamiaternifolia*, J.F. Dillon (1969-045–047) (3 slides); Miami-Dade Co., Miami, January 22, 1975, on *Callistemonviminalis*, D. Sager (1975-2885) (3 slides); Miami-Dade Co., Miami, July 27, 1978, on *Mangifera* sp., M. Corman (1978-3308) (2 slides); Miami-Dade Co., Miami, April 5, 1979, on *Kigeliapinnata*, P. Chobrda (1979-2913); Miami-Dade Co., Miami, November 5, 1979, on *Callistemon* sp., H. VonWald (1979-3003); Miami-Dade Co., Miami, April 1, 1980, on *Callistemonviminalis*, G. Webster and E. Pena (1980-014); Miami-Dade Co., Miami, April 1, 1980, on *Callistemonviminalis*, G. Webster and E. Pena (1980-2881); Miami-Dade Co., Miami, April 2, 1980, on *Santalumalbum*, H. Von Wald and C. Dowling (1980-2935) (2 slides); Miami-Dade Co., Miami, April 3, 1981, on *Perseaamericana*, K. Martin (1981-016 –020) (5 slides); Miami-Dade Co., Miami, March 3, 1981, on *Macadamiaternifolia*, W. James (1981-2934) (2 slides); Miami-Dade Co., Miami, March 25, 1981, on *Macadamiaternifolia*, W. James (1981-2984); Miami-Dade Co., Miami, February 10, 1982, on *Diospyroslotus*, H. VonWald (1982-017–019) (3 slides); Miami-Dade Co., Miami, April 8, 1982, on *Eucalyptus* sp., P. Perun (1982-223, 2853) (2 slides); Miami-Dade Co., Miami, April 8, 1982, on *Eucalyptus* sp., P. Perun (1982-2853); Miami-Dade Co., Miami, November 15, 1985, on *Howeaforsteriana*, D. Chalot (1985-3009) (2 slides); Miami-Dade Co., Miami, September 19, 29, 1986, on *Perseaamericana*, D. Storch (1986-021–023, 3091) (3 slides); Miami-Dade Co., Miami, January 17, 2001, *Manilkararoxburghiana*, E. Putland (2001-189) (2 slides); Miami-Dade Co., Miami, March 14, 2002, on *Perseaamericana*, L. Davis (2002-870); Miami-Dade Co., Miami, August 14, 2007, on *Manilkararoxburghiana*, O. Garcia (2002-5912); Miami-Dade Co., Miami, May 11, 2012, on *Laurusnobilis*, M. Figueroa (2012-3720) (2 slides); Miami-Dade Co., North Beach, January 20, 1981, on *Amyriselemfera*, E.W. Campbell and R. Kendrick (1981-2947) (2 slides); Miami-Dade Co., Opa Locka, October 5, 1977, on *Callistemon* sp., M. Corman (1977-3268, 3278) (2 slides); Miami-Dade Co., Opa Locka, May 22, 1978, on *Perseaamericana*, J. Hilderbrandt (1978-3313) (2 slides); Miami-Dade Co., West Miami, November 22, 1977, on *Camellia* sp., D. Martinelli (1977-2888, 2898) (2 slides); Monroe Co., Little Torch Key, April 10, 2018, on *Bidensalba*, P. Corogin, J. Hayden, E. Talamas, B. Danner, J. Farnum (2018-1780); Orange Co., Apopka, Jan 10, 2001, on *Ravenearivularis*, K. Gonzalez (2001-116) (2 slides); Orange Co., Belle Isle, January 20, 2006, on *Garcinialivingstonei*, T. Williams (2006-268); Orange Co., Orlando, February 2013, on Theaceae, A. Puppelo (2013-1127); Orange Co., Orlando, May 27, 2008, on *Magnoliavirginiana*, A. Puppelo (2008-3337); Orange Co., Winter Garden, October 31, 2008, on *Machilusthunbergii*, G. Warden (2008-7478); Orange Co., Zellwood, March 7, 2019, *Laurusnobilis*, K. Gonzalez (2019-1006) (2 slides); Palm Beach Co., Boca Raton, May 19, 1982, on *Chamaeropshumilis*, D.C. Clinton (1982-487) (3 slides); Palm Beach Co., Boynton Beach, October 10, 1973, on *Ficus* sp., K. Geyer (1973-3284) (3 slides); Palm Beach Co., Delray Beach, May 12, 1978, on *Diospyros* sp., K.C. Stolley (1978-2852) (2 slides); Palm Beach Co., Boynton Beach, June 7, 1978, on *Mimusops* sp., K. Stolley (1978-3307); Palm Beach Co., Boynton Beach, September 27, 1991, on *Camelliajaponica*, E. Tannehill (1991-2906); Palm Beach Co., Boynton Beach, January 13, 1988, on *Mimusopsroxburghiana*, D. Leone (1988-2880) (2 slides); Palm Beach Co., Boynton Beach, November 2, 1989, on *Sisyrinchiumsolstitiale*, E. Tannehill (1989-485, 3303) (2 slides); Palm Beach Co., Delray Beach, February 23, 1988, on *Melaleuca* sp., E. Tannehill and A. Hamon (1988-2851) Palm Beach Co., Jupiter, May 8, 2013, on *Magnolia* sp., L. West (2013-3217) Palm Beach Co., Lake Park, June 14, 1978, on *Chrysalidocarpuslutescens*, J. Bennet (1978-3567) (2 slides); Palm Beach Co., Lake Park, April 24, 1979, on *Chrysalidocarpuslutescens*, J.E. Bennet (1979-2920) (2 slides); Palm Beach Co., Lake Worth, March 7, 1978, on *Kentia* sp., J, Bennett (1978-3285); Palm Beach Co., Lake Worth, October 8, 1981, on *Magnoliavirginiana*, J. Fellers and R. Buchholz (1981-026–027) (2 slides); Palm Beach Co., Lake Worth, July 13, 1995, on *Chrysalidocarpuslutescens*, Cook S.H., Clinton D.C. (1995-3024); Palm Beach Co., Lake Worth, July 13, 1995, on *Chrysalidocarpuslutescens*, S.H. Cook, D.C. Clinton (1995-3024); Palm Beach Co., Lake Worth, March 25, 2004, on *Calophylluminophyllum*, L. Smith (2004-2103); Palm Beach Co., Pahokee, February 22, 1980, on *Chrysalidocarpuslutescens*, N. Miles and B. Walsh (1980-2955) (2 slides); Palm Beach Co., South Bay, November 14, 2018, on *Magnoliavirginiana*, J. Farnum (2018-5955); Palm Beach Co., West Palm Beach, January 14, 1991, on *Mangiferaindica*, R.T. Doll (1991-3566) (2 slides); Pinellas Co., Clearwater, January 11, 2013, on *Perseapalustris*, W. Salway (2013-575) (2 slides); Pinellas Co., Indian Rocks, October 3, 1972, on *Perseaamericana*, K.C. Lowery (1972-2860); Pinellas Co., Largo, November 8, 1978, on *Persea* sp., P. Pullara (1978-3306); Pinellas Co., St. Petersburg, August 15, 1967, on *Persea* sp., C.K. Hickman (1967-2854) (3 slides); Pinellas Co., St. Petersburg, February 2, 2008, on *Perseaborbonia*, M. Spearman (2004-724); Pinellas Co., St. Petersburg, May 28, 2009, on *Perseaborbonia*, M. Spearman (2009-3632); Polk Co., Cypress Gardens, January 16, 1962, on *Tetrapanax* sp., J.N. Pott (1962-3266) (2 slides); Polk Co., Cypress Gardens, August 13, 1964, on *Magnolia* sp. W.P. Henderson (1964-2905) (2 slides); Polk Co., Lake Wales, October 25, 1962, on *Ficus* sp., Ralph E. Brown (1962-2857); Polk Co., Winter Haven, April 8, 1980, on *PerseaAmericana*, H.G. Schmidt (1980-2938) (2 slides); Polk Co., Winter Haven, July 26, 2018, on *Laurusnobilis*, J. Bryan (2018-4054); St. Lucie Co., Fort Pierce, March 23, 1978, on *Paurotis* sp., E.W. Campbell (1978-032–033) (2 slides); St. Lucie Co., Fort Pierce, January 17, 1979, on *Persea* sp., E.W. Campbell (1979-2893); St. Lucie Co., Fort Pierce, March 23, 1979, on *Paurotis* sp., E.W. Campbell (1979-2900); St. Lucie Co., Fort Pierce, February 24, 1984, on *Bumeliatenax*, K. Hibbard and E.W. Campbell (1984-492, 2948) (2 slides); St. Lucie Co., Fort Pierce, November 6, 1985, on *Paurotis* sp., K Hibbard and E.W. Campbell (1985-484, 2950) (2 slides); St. Lucie Co., Fort Pierce, March 13, 2003, on *Phoradendronleucarpum*, K. Hibbard (2003-927); St. Lucie Co., Fort Pierce, February 21, 2005, on *Ilexcornuta*, D. Vazquez (2005-4069); St. Lucie Co., Hutchinson, Isle, April 18, 1980, on *Eugeniasimpsonii*, E.W. Campbell (1980-2990); St. Lucie Co., Port St. Lucie, May 17, 1978, on *Perseaborbonia*, E.W. Campbell (1978-2858) (2 slides); St. Lucie Co., Port St. Lucie, February 20, 1980, on *Perseaborbonia*, E.W. Campbell and R.H. Kendrick (1980-2989, 3008) (2 slides); St. Lucie Co., White City, May 30, 1980, on *Perseaborbonia*, E.W. Campbell (1980-2961) (2 slides); Volusia Co., Allandale, March 16, 1983, on *Hederahelix*, J.N. Pott (1983-486) (2 slides); Volusia Co., Daytona Beach, August 16, 1984, on *Howeaforsteriana*, J.N. Pott (1984-2994); Volusia Co., Holly Hill, March 15, 1956, on *Chamaedorea* sp., C.R. Roberts (1956-2877) (3 slides); Volusia Co., New Smyrna Beach, April 8, 1985, on Palm, J.N. Pott (19852942); Volusia Co., New Smyrna Beach, September 27, 1971, on *Camellia* sp., J.N. Pott (1971-2887, 2889) (8 slides); Walton Co., Walton, February 12, 1980, on *Mangiferaindica*, E.W. Campbell (1980-2946) (2 slides).

**Figure 7.**
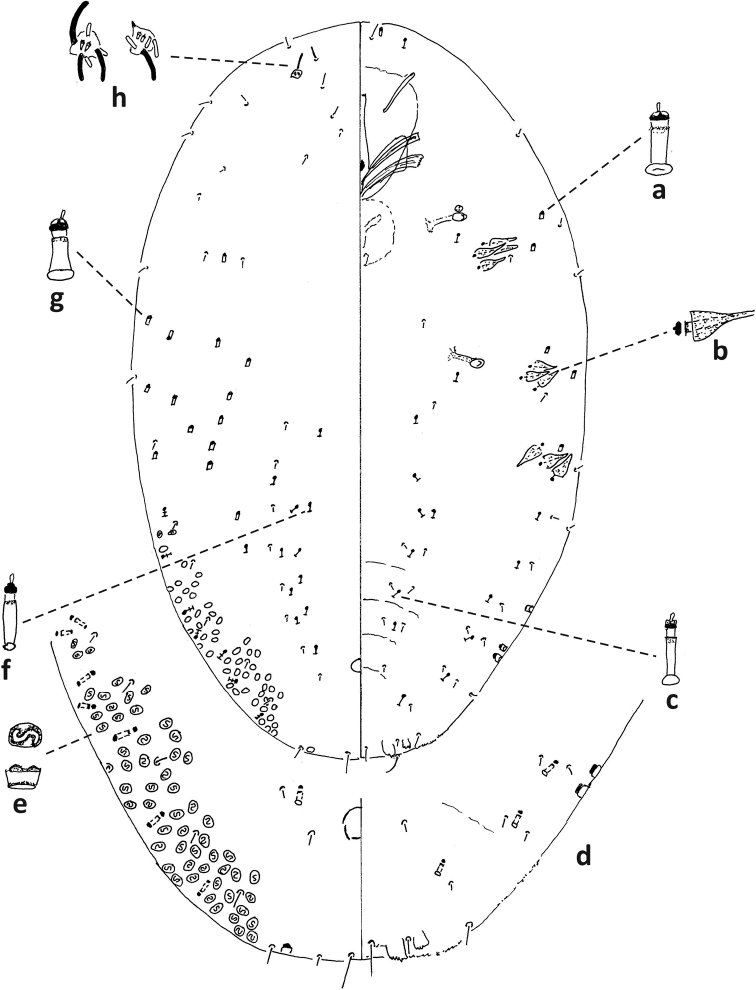
*Fioriniafioriniae*, second-instar male, Marion Co., Ocala, August 13, 2019 on *Chamaeropshumilis*, T. Gordon, (2019-4546). Abbreviations: a) large microduct; b) large gland spine; c) large gland spine; d) large microduct; e) small gland c) small microduct; d) enlargement of pygidium; e) pores with S-shaped opening f) dorsal large microduct; g) dorsal large microducts; h) antennae each with several enlarged seta.

##### Specimens examined for description and diagnosis.

Marion Co., Ocala, August 13, 2019 on *Chamaeropshumilis*, T. Gordon, 5 2^nd^ ♀, 5 2^nd^ ♂, 10 ad ♀ (2019-4546).

##### Other material examined from USNM.

Mexico: July 11, 1988, on *Mangiferaindica*, S. Sanner 6 2^nd^ ♀ (El Paso 032924). Peru: May 7, 1977, on *Mangiferaindica*, R. Narkaus 5 2^nd^ ♀ (Los Angeles 19190); August 21, 1972, on *Camellia* sp., E.B. Lee 1 1^st^ ♀, 4 2^nd^ ♀, 3 ad ♀. Portugal: Azores, August 20, 1928, on *Camellia* sp., C.A. Davis 1 1^st^ ♀ (at Providence, Rhode Island). United States: California, San Diego, San Diego Zoo, August 19, 2002, D. Kellum, J.F. Miller, D.R. Miller, on *Camellia* sp. 3 1^st^ ♀, 18 2^nd^ ♀, 7 ad ♀; Georgia, Camden Co., June 14, 1969, on *Ruscus* sp., R.J. Beashear 1 1^st^ ♀.

#### Fiorinia japonica

Taxon classificationAnimaliaHemipteraDiaspididae

Kuwana, 1902

11964313-1155-590A-8A4B-27CDEE550E64

##### Field characteristics.

First-instar exuviae overlapping second-instar exuviae. Without indentation or with slight indentation formed between attachment of first- and second-instar exuviae. Second-instar exuviae oval, convex marginally; medium to dark brown; longitudinal ridge inconspicuous. Posterior end of adult female within second-instar exuviae rounded. Heavily infested leaves with white secretion (Suppl. material 1: Fig. S1).

##### First-instar nymph.

described in Howell (1977).

##### Second-instar female.

Median lobes broad, as wide as or wider than medial lobule of second lobe, projecting ca. same distance as or further than medial lobule of second lobes. With five pairs of marginal macroducts. Swelling of body margin adjacent to macroduct usually rounded. With four large gland spines on margin of each side of body from abdominal segments II–V; usually without small gland spine on each side of abdominal segment VI; with small gland spines on margin or submargin of abdominal segment I. With one microduct on each side of head. Longitudinal line of microducts present submarginally on venter of abdominal segment II or III–VI, normally with one microduct on each side of each segment. Cicatrices absent.

##### Second-instar male.

One duct cluster on each side of body, composed of several small ducts and two communal ducts. Three longitudinal lines of microducts on venter of abdomen (one medial and two submarginal). Cluster of small microducts with sclerotized orifice laterad of anterior spiracle absent. Fewer than five gland spines on each side of body between anterior and posterior spiracles. Antennae each with one enlarged seta.

**Figure 8.**
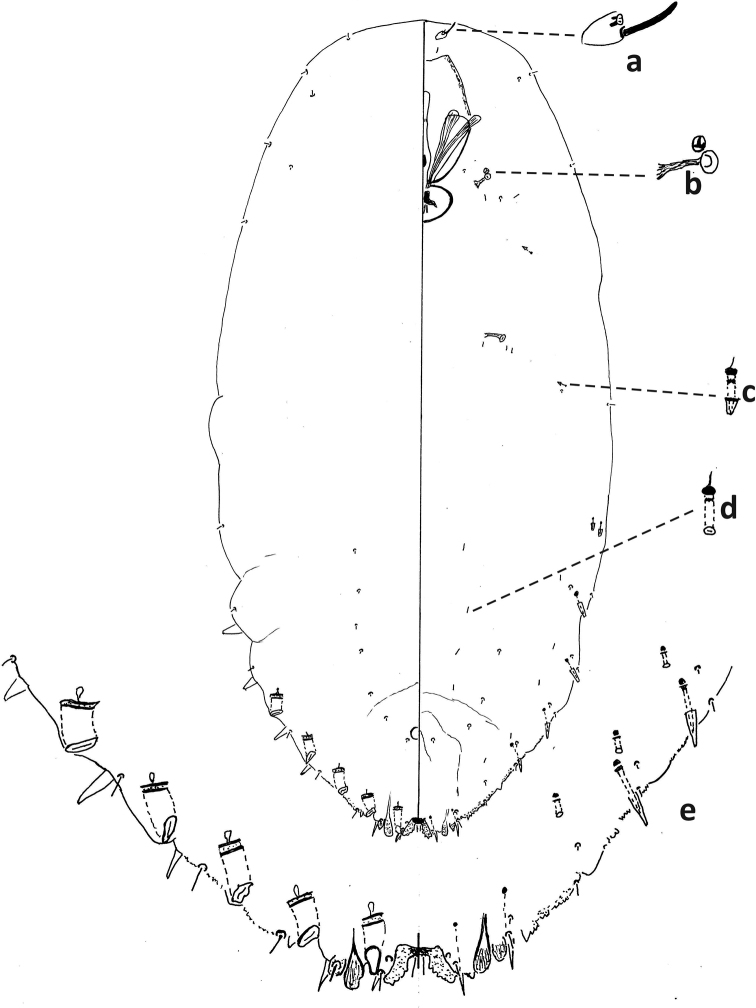
*Fioriniajaponica* second-instar female, Virginia, Chesterfield Co., Southside Nursey, July 27, 1974, on blue spruce, R. Sears. Abbreviations: a) antenna; b) anterior spiracle; c) microduct with sclerotized orifice; d) small microduct; e) enlargement of pygidium.

**Figure 9.**
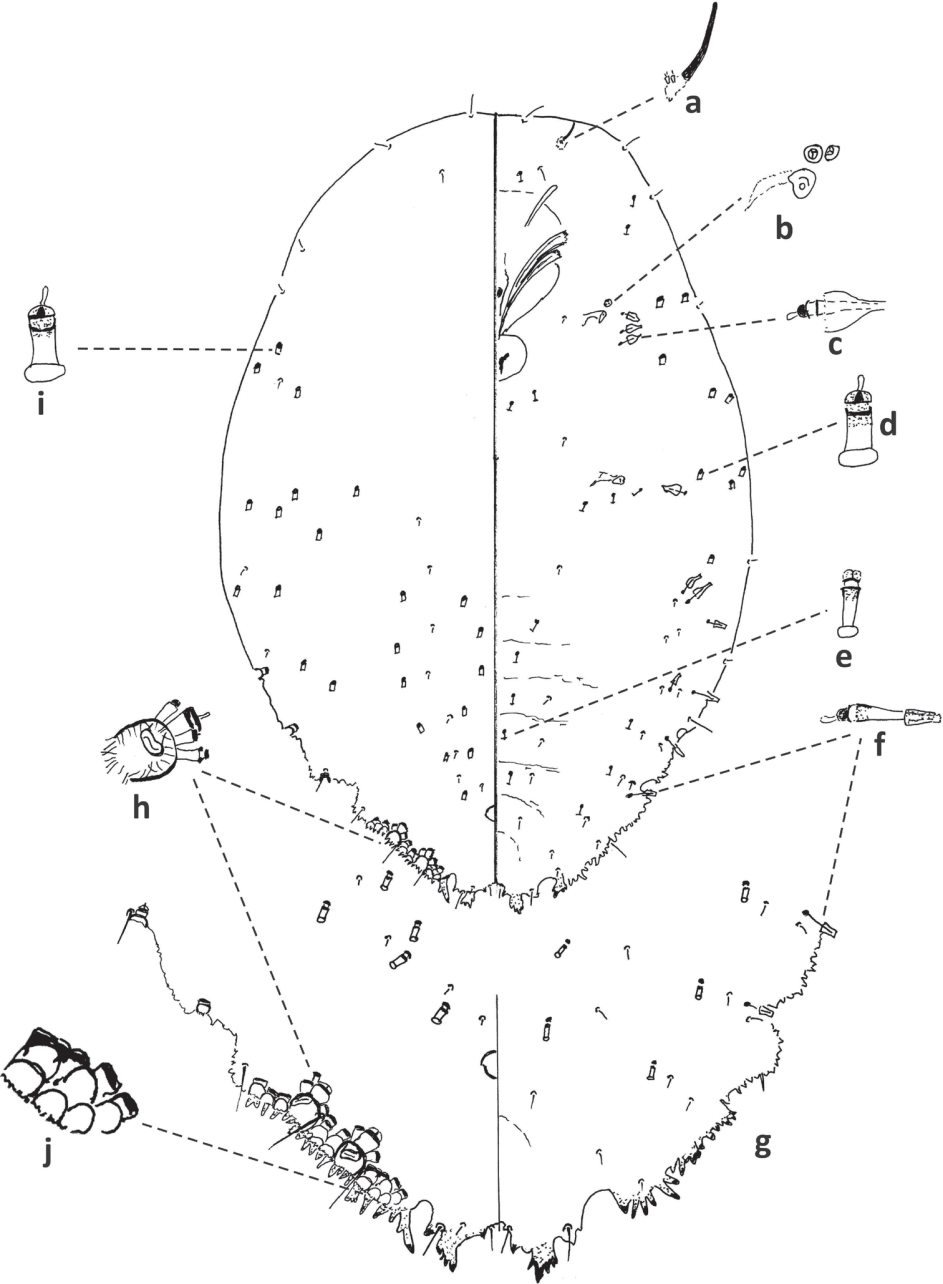
*Fioriniajaponica*, second-instar male Virginia, Chesterfield Co., Southside Nursey, July 27, 1974, on blue spruce, R. Sears. Abbreviations: a) antenna; b) anterior spiracle; c) large gland spine; d) large microduct; e) small microduct; f) small gland spine; g) enlargement of pygidium; h) enlargement of portion of duct cluster; i) large microduct; j) enlargement of communal duct.

##### Florida collection records.

*Fioriniajaponica* has not been collected in Florida.

##### Specimens examined for description and diagnosis.

Virginia, Chesterfield Co., Southside Nursey, July 27, 1974, on blue spruce, R. Sears, 5 2^nd^ ♀, 5 2^nd^ ♂.

##### Other material examined from USNM.

China; “Hsifeushang” January 23, 1933, on *Pinus* sp., W.B. Wood 2 1^st^ ♀ (JOH 55-76 F). Taiwan: Maruyama, near Taihoku, June 3, 1928, on *Pinusthunbergi*, R. Takahashi 1 1^st^ ♂ (JOH 58-76); Taihoku, June 7, 1929, on *Pinus* sp., R. Takahashi 1 1^st^ ♀ (JOH 54-76). United States: Virginia, Chesterfield Co., Southside Nursey, July 27, 1974, on blue spruce, R. Sears 2 1^st^ ♀, 1 1^st^ ♂, 5 2^nd^ ♀, 5 2^nd^ ♂, 4 ad ♀; Washington, D.C., August 27, 1991, on national Christmas tree, Horton 3 1^st^ ♀, 4 2^nd^ ♀, 3 ad ♀ (93-09742).

#### Fiorinia phantasma

Taxon classificationAnimaliaHemipteraDiaspididae

Cockerell & Robinson, 1915

E6A47FC2-0C6B-5E69-B4E5-3AD16F1AFCAC

##### Field characteristics.

First-instar exuviae overlapping second-instar exuviae. Without indentation formed between attachment of first- and second-instar exuviae. Second-instar exuviae oval, convex marginally; light to dark brown, longitudinal ridge weakly developed. Posterior end of adult female within second-instar exuviae constricted and pointed (Suppl. material 1: Fig. S1).

##### First instar.

Similar to *F.fioriniae* and *F.proboscidaria* in having gland spines on abdominal segment VI at least half as long as gland spine on segment VII. *Fioriniafioriniae* and *F.proboscidaria* differ by having (characters in parentheses are those of *P.phantasma*): pattern of derm surrounding large duct on head serpentine (globular); inner apex of large duct on head flat (rounded or mushroom like).

##### Second-instar female.

Median lobes broad, as wide as or slightly narrower than medial lobule of second lobe, projecting ca. same amount or slightly less than medial lobule of second lobes. With five pairs of marginal macroducts. Swelling of body margin adjacent to macroduct usually pointed. With three large gland spines on margin of each side of body from abdominal segments II–IV, without gland spine on abdominal segment VI; with small gland spines on margin or submargin of abdominal segment I. With three microducts on each side of head. Longitudinal line of microducts absent submarginally on venter of abdomen. Cicatrices absent.

##### Second-instar male.

One duct cluster on each side of body, composed of several small ducts and one communal duct. Five longitudinal lines of microducts on venter of abdomen (one medial, two mediolateral, and two submarginal), medial line sometimes incomplete. Cluster of small microducts with sclerotized orifice laterad of anterior spiracle absent. Fewer than five gland spines on each side of body between anterior and posterior spiracles. Antennae each with one enlarged seta.

##### Adult female.

Body tapering at segment III to narrow pygidium. With three or four pairs of dorsal macroducts on each side of body, ducts similar in shape and size to microducts. Projection between antennae with many spicules. Antennae close together, with distinct projection.

##### Florida collection records.

Miami-Dade Co., Miami, March 1, 2018, on *Phoenixcanariensis*, Olga Garcia (2018-789) (3 slides); Miami-Dade Co., Coral Gables, April 2, 2018, on *Phoenix* sp., J. Farnum (2018-1499) (2 slides); Miami-Dade Co., Pinecrest, April 2, 2018, on *Phoenix* sp., J. Farnum (2018-1487, 1489, 1491, 1492, 1496, 1500, 1503, 1504, 1524, 1525) (20 slides); Miami-Dade Co., Palmetto Bay, April 2, 2018, on *Phoenix* sp., J. Farnum (2018-1488, 1493, 1498, 1501) (8 slides); Miami-Dade Co., Pinecrest, May 22, 2018, on *Phoenix* sp., J. Farnum and J. Vergel (2018-2780, 2785, 2796) (6 slides); Palm Beach Co., Boynton Beach, January 23, on *Cocosnucifera*, L. Smith (2018-304) (2 slides); Miami-Dade Co., Coral Gables, Miami-Dade Co., Palmetto Bay, May 22, 2018, on *Phoenix* sp., J. Farnum and J. Vergel (2018-2783, 2788, 2790) (6 slides); May 22, 2018, on *Dypsislutescens*, J. Miller, H. Mayer, M.Z. Ahmed (2018-2761) (2 slides); Miami-Dade Co., Coral Gables, May 22, 2018, on *Phoenixreclinata*, H. Mayer, J. Miller, M.Z. Ahmed (2018-2767); Miami-Dade Co., Kendall, May 22, 2018, on *Phoenix* sp., J. Farnum and J. Vergel (2018-2787, 2771) (6 slides); Miami-Dade Co., Miami, May 22, 2018, on *Phoenixroebelenii*, E. Talamas, V. de Campover, C. Mannion (2018-2779); Miami-Dade Co., Miami, May 22, 2018, on *Phoenix* sp., L. Osborne, Y. Hernandez, P. Perez (2018-2794); Miami-Dade Co., Miami, May 22, 2018, on *Cycasrevoluta*, L. Osborne, Y. Hernandez, P. Perez (2018-2854) (2 slides); Miami-Dade Co., Miami, May 22, 2018, on *Phoenix* sp., J. Farnum and J. Vergel (2018-2784); Miami-Dade Co., Miami, May 22, 2018, on *Phoenix* sp., L. Osborne and Y. Hernandez (2018-2785); Miami-Dade Co., Miami, May 23, 2018, on *Phoenix* sp., J. Farnum and J. Vergel (2018-2782, 2797, 2799) (6 slides); Miami-Dade Co., Miami, May 23, 2018, on *Phoenix* sp., J. Farnum, C.M. Twyford (2018-2766) (2 slides); Miami-Dade Co., Miami, May 24, 2018, on *Phoenixroebelenii*, J. Farnum and C.C. Twyford (2018-2795); Miami-Dade Co., Miami, May 24, 2018, on *Phoenix* sp., C.M. Twyford (2018-2759, 2765, 2776, 2777, 2786, 2798) (12 slides); Miami-Dade Co., Coral Gables, May 24, 2018, on *Strelitzia* sp., O. Garcia and M.Z. Ahmed (2018-2774) (2 slides); Miami-Dade Co., Coral Gables, May 24, 2018 on *Cocosnucifera*, O. Garcia and M.Z. Ahmed (2018-2773); Miami-Dade Co., Coral Gables, October 15, 2018, on *Tahinaspectabilis*, C.T. Allen, J. Farnum, S. Durand, A. Roda (2018-5459) (2 slides); Miami-Dade Co., Coral Gables, October 26, 2018, on *Pittosporumtobira*, J. Farnum, C.T. Allen, A. Roda (2018-5679) (2 slides); Miami-Dade Co., Coral Gables, October 26, 2018, on *Livistonachinensis*, J. Farnum, C.T. Allen, A. Roda (2018-5680) (2 slides); Miami-Dade Co., Coral Gables, December 3, 2018, on *Sabalmexicana*, J. Farnum, L. Noblick (2018-6221) (2 slides); Miami-Dade Co., Coral Gables, December 3, 2018, on *Nypafruticans*, J. Farnum and L. Noblick (2018-6222) (2 slides); Miami-Dade Co., Coral Gables, December 3, 2018, on *Tahinaspectabilis*, J. Farnum, L. Noblick (2018-6218); Miami-Dade Co., Coral Gables, December 3, 2018, on *Howeaforsteriana*, J. Farnum and L. Noblick (2018-6219) (3 slides); Palm Beach Co., Boynton Beach, February 8, 2019, on *Pandanus* sp., L. Smith (2018-481) (2 slides); Palm Beach Co., Delray Beach, March 4, 2019, on unknown host, J. Farnum and L. Smith (2018-903) (2 slides).

##### Specimens examined for description and diagnosis.

Palm Beach Co., Boynton Beach, October 30, 2019, on *Wodyetiabifurcata*, L. Smith, 5 1^st^ (2019-5998); Palm Beach Co., Boynton Beach, April 6, 2020, on *Ligustrumjaponicum*, L. Smith, 10 ad ♀ (2020-1365); Palm Beach Co., Boynton Beach, November 6, 2019, on *Wodyetiabifurcata*, 5 2^nd^ ♀ (2019-6182); Philippines, June 28, 1996 on *Plumeria* sp., 2^nd^ ♀ (SF023635); Miami-Dade Co., Miami, November 9, 2019, on Palmae, O. Garcia, 5 2^nd^ ♂ (2019-6149); Palm Beach Co., Boca Raton, December 29, 2020, on *Phoenixcanariensis*, L. Smith, 10 ad ♀ (2020-4958).

**Figure 10.**
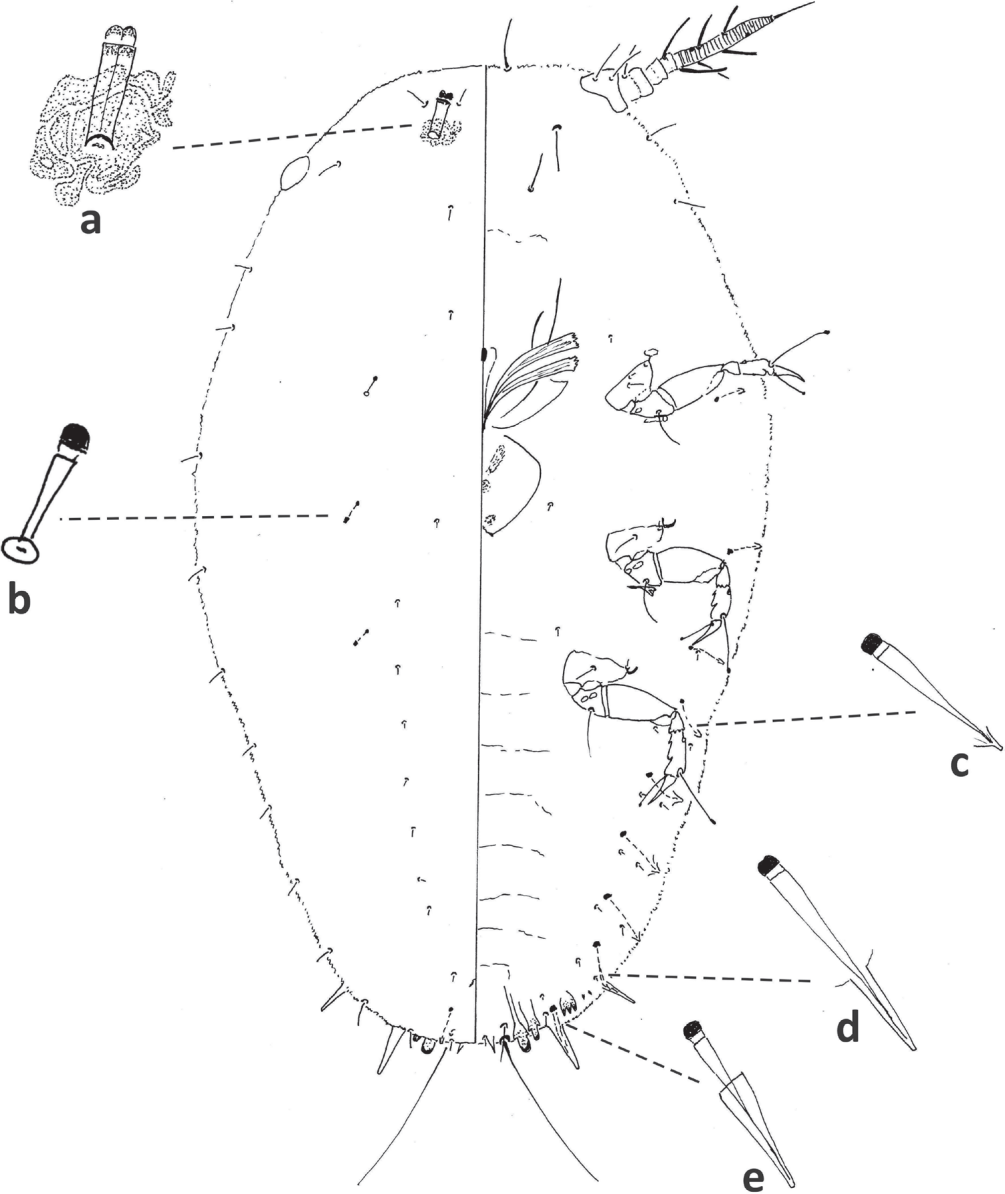
*Fioriniaphantasma*, First-instar nymph, Palm Beach Co., Boynton Beach, October 30, 2019, on *Wodyetiabifurcata*, L. Smith, (2019-5998). Abbreviations: a) large dorsal duct on head; b) small microduct; c) gland spine abdominal segment II with small projection; d) gland spine at abdominal segment VI with long projection; e) gland spine at abdominal segment VII with long projection.

**Figure 11.**
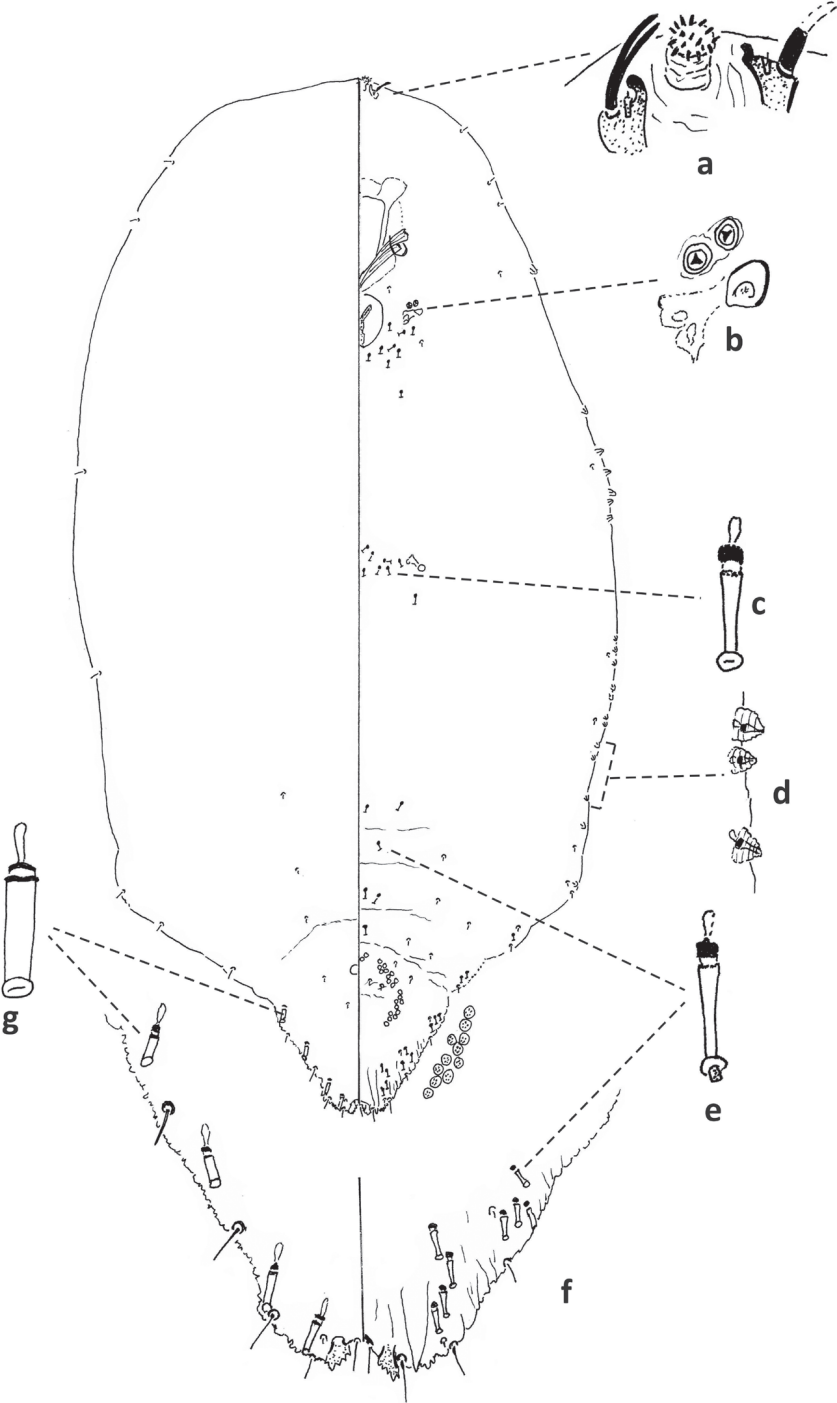
*Fioriniaphantasma* (Cockerell & Robinson); adult female, Palm Beach Co., Boynton Beach, April 6, 2020, on *Ligustrumjaponicum*, L. Smith (2020-1365). Abbreviations: a) detail of antennae and inter-antennal process; b) detail of anterior spiracle; c) microducts; d) marginal duct tubercles; e) marginal microduct; f) detail of pygidium; g) marginal macroduct on pygidium.

**Figure 12.**
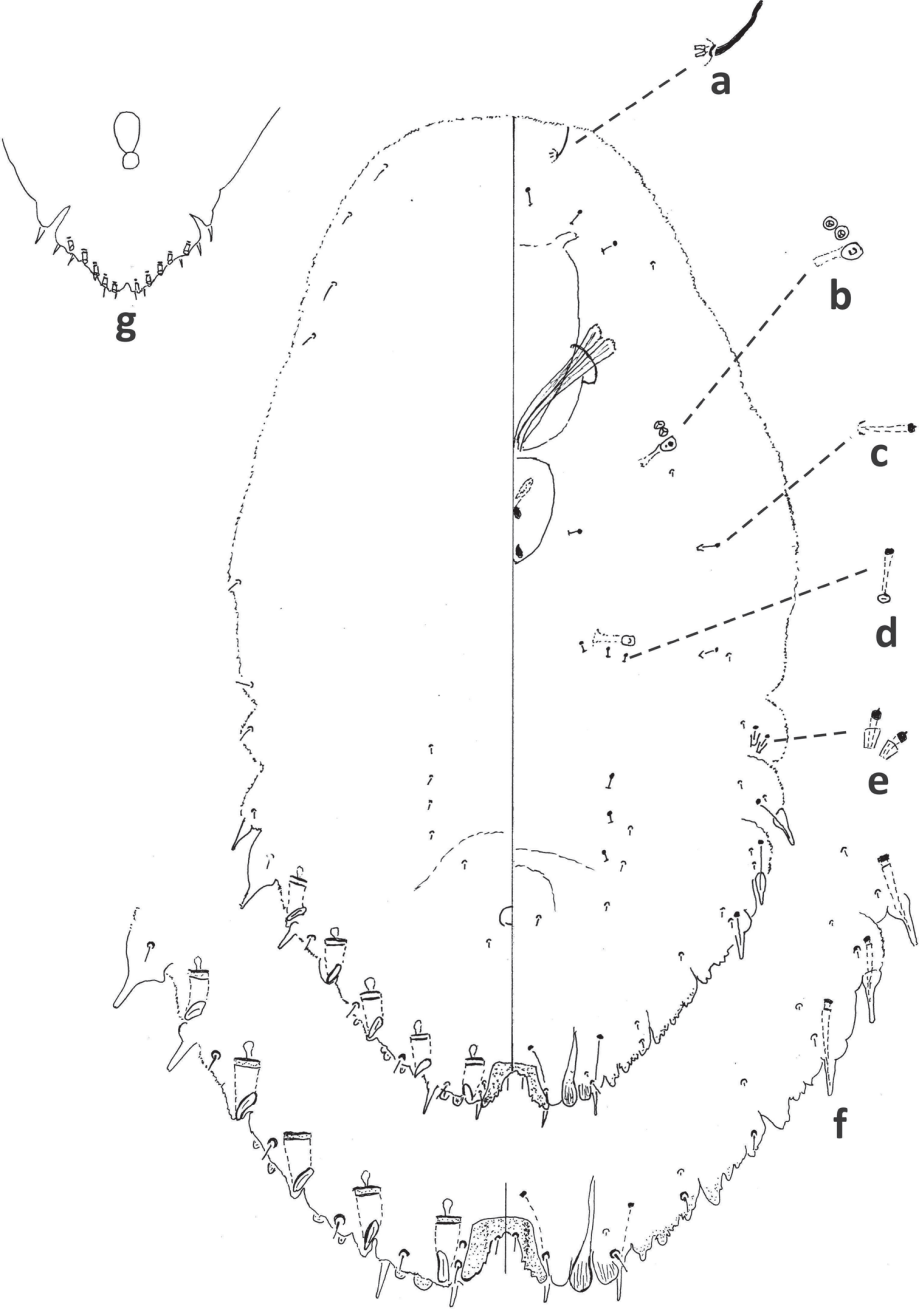
*Fioriniaphantasma*, second-instar female and second-instar female shed shin, Palm Beach Co., Boynton Beach, November 6, 2019, on *Wodyetiabifurcata*, (2019-6182). Abbreviations: a) antenna; b) anterior spiracle; c) microduct with sclerotized orifice; g) old second-instar female, Philippines, June 28, 1996 on *Plumeria* sp., 2^nd^ ♀ (SF023635).

**Figure 13.**
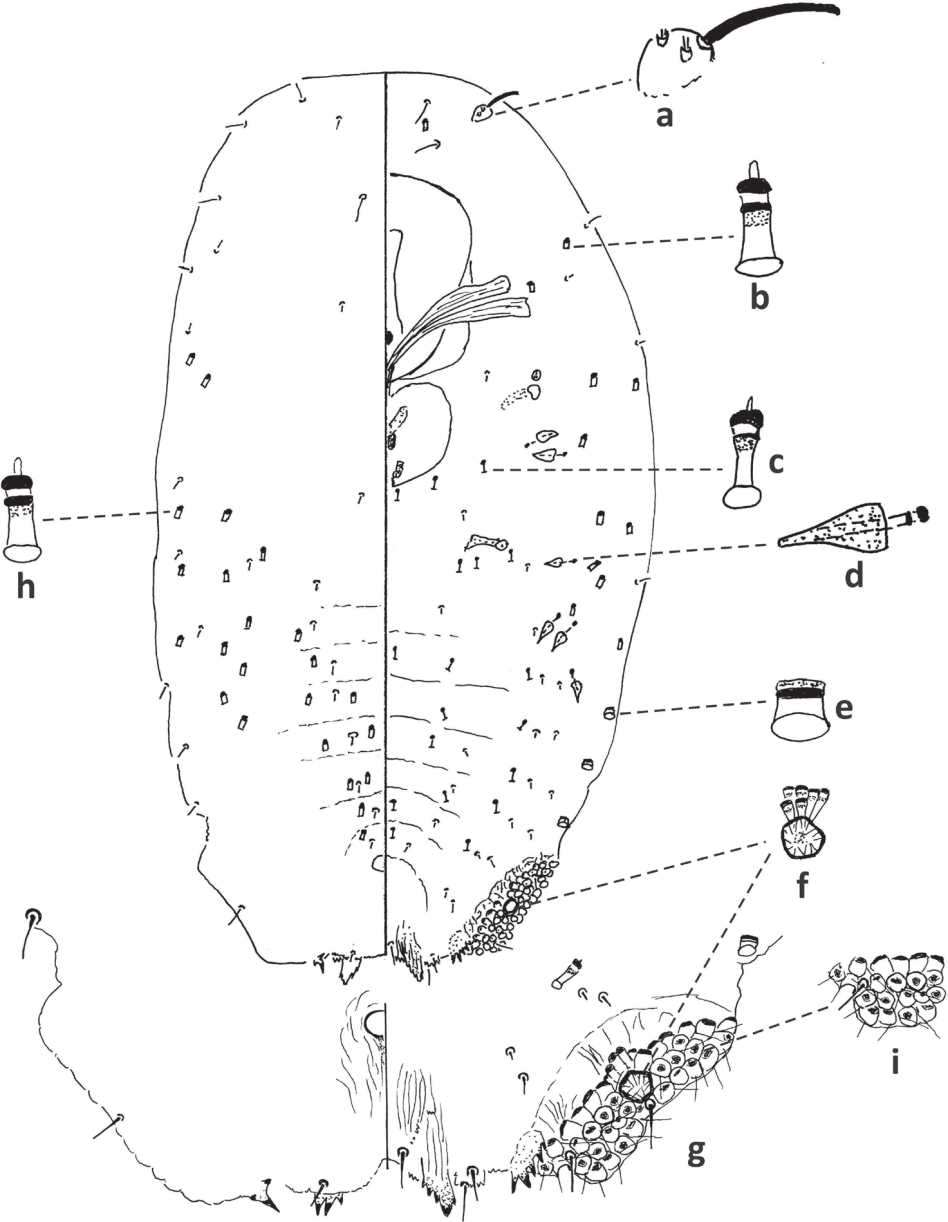
*Fioriniaphantasma*, second-instar male, Miami-Dade Co., Miami, November 9, 2019, on Palmae, O. Garcia, (2019-6149). Abbreviations: a) antenna; b) large microduct; c) small microduct; d) large gland spine; e) small macroduct; f) enlargement of communal duct ; g) enlargement of pygidium; h) large microduct; j) enlargement of part of duct cluster.

##### Other specimens examined from USNM.

Grenada, Calivingy Island, March 2012, on *Phoenixdactylifera*, S.W. Evans (E-2012-2099); Guam, Tamuning, June 4, 1984, on *Cocosnucifera*, R. Muniappan; Hawaii: Oahu, Kapahulu area, March 27, 2009, on *Ligustrum* sp., M. Ramadan (0904651); Hawaii, Hilo ?, date ?, on *Pittosporum* sp., B. Kumashiro 5 2^nd^ ♀,5 2^nd^ ♀; Philippines, April 7, 1965, on *Cocosnucifera*, J.I. Mason; Philippines, November 15, 1971, on palm leaf, R.F. Goodall (Seattle 8910); Philippines, March 13, 1975, on *Mangiferaindica*, M. Yoshinaga (Hawaii 28847); Philippines, August 26, 1975, on *Cocosnucifera*, A. Buchanan (LA 015303); Philippines, November 30, 1975, on palm leaf, Ozuka & Richardson (Hawaii 32858); Philippines, June 8, 1977, on palm leaf, Tamiya (Hawaii 39130); Philippines, August 17, 1977, on *Areca* sp., J. Sato (Honolulu 42721); Philippines, October 19, 1978, on *Areca* sp., Takeda, (Honolulu 43108); Philippines, November 4, 1978, on palm leaf, Jodoi, (Honolulu 42704); Philippines, May 26, 1981, on *Tamarindusindica*, D.O. Wienhee & F.G. Walisen (Seattle 17304); Philippines, May 12, 1985, on Philippines, *Clauseniaanisum*, C. Dollopf (Chicago 009312); Philippines, June 28, 1996, on *Plumeria* sp. (SF 023635); Philippines, October 19, 1978, on *Areca* sp., Takeda (Honolulu 43108); Taiwan, May 10, 1981, on leaf, J.L. Levitt (Seattle 17328); Taiwan, April 8, 1988, on *Ficus* sp., V. McDonald (JFK 100609); Thailand, August 31, 1982, on leaf, G. Hinsdale (Anchorage 017131); Thailand, July 19, 1985, on *Murrayakoenigii*, J. Alabu (LA 052785); Thailand, November 15,1987, on *Areca* sp., J. Elridge (Atlanta 003471); Thailand, March 20, 2003, on Arecaceae, F. Hadded; Thailand, March 27, 2006, on palm, M. Hanzlik (ANC 060060); Vietnam, April 26, 2007, on *Cocos* sp., A. Coronel (LA 207977 CA); Vietnam, October 26, 2012, on unknown host, D. Gregory (SF 1301449).

##### Notes.

We examined four paratype slides of *F.coronata* Williams & Watson from Guadalcanal, Solomon Islands deposited in the USNM collection at Beltsville, Maryland. Most of the specimens were punctured in the middle of the body between the posterior spiracles during the mounting process. However, we could still see that all had microducts between the posterior spiracles which were small and less numerous than specimens from elsewhere, but they definitely are there.

We also examined a paratype slide of *F.phantasma* in the same collection, but it is in such poor condition that only half of the pygidium is useful for diagnosis. It is impossible to even find the posterior spiracles, let alone microducts between them. In addition, the holotype of *F.phantasma* deposited in The Natural History Museum, London (NHMUK) was loaned to and examined by one of us (DL). It also was in poor condition; microducts close to anterior and posterior spiracles and in prepygidial abdominal segments were not visible. All of the examined specimens of *F.phantasma* that were in good condition had easily discernable microducts between the posterior spiracles.

#### Fiorinia pinicola

Taxon classificationAnimaliaHemipteraDiaspididae

Maskell, 1897

AD947CA6-2C15-5487-B08E-06069C806CDD

##### Field characteristics.

First-instar exuviae overlapping second-instar exuviae. With indentation formed between attachment of first- and second-instar exuviae. Second-instar exuviae oval, convex marginally; medium to dark brown; longitudinal ridge conspicuous. Posterior end of adult female within second-instar exuviae rounded. Heavily infested leaves with white secretion (Suppl. material 1: Fig. S1).

##### First-instar nymph.

Described in Howell (1977).

##### Second-instar female.

Median lobes broad, as wide as or wider than medial lobule of second lobe, projecting ca. same amount as medial lobule of second lobes. With five pairs of marginal macroducts. Swelling of body margin adjacent to macroduct usually pointed. With four large gland spines on margin of each side of body from abdominal segments II–V; usually without small gland spine on each side of abdominal segment VI; with small gland spines on margin or submargin of abdominal segment I. With one microduct on each side of head. Longitudinal line of microducts present submarginally on venter of abdominal segments II–VI, normally with one microduct on each side of each segment. Cicatrices absent.

**Figure 14.**
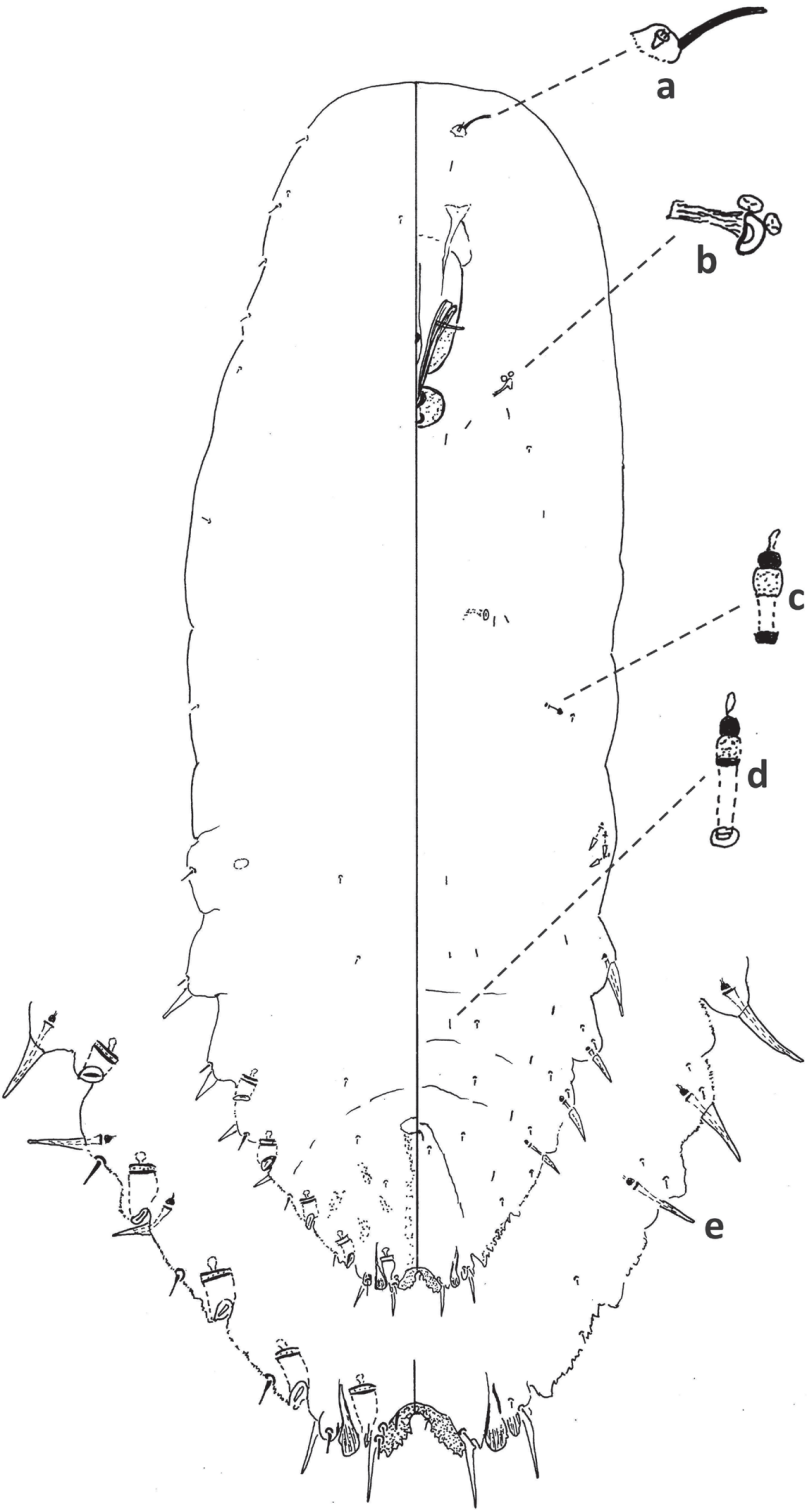
*Fioriniapinicola*, second-instar female, Hong Kong, December 1895, on *Pinussinensis*, A. Koebele (1529) mounted from type material. Abbreviations: a) antenna; b) anterior spiracle; c) small microduct with sclerotized orifice d) small microduct e) enlargement of pygidium. Note the blank blotches on the body margin of the pygidium enlargement.

**Figure 15.**
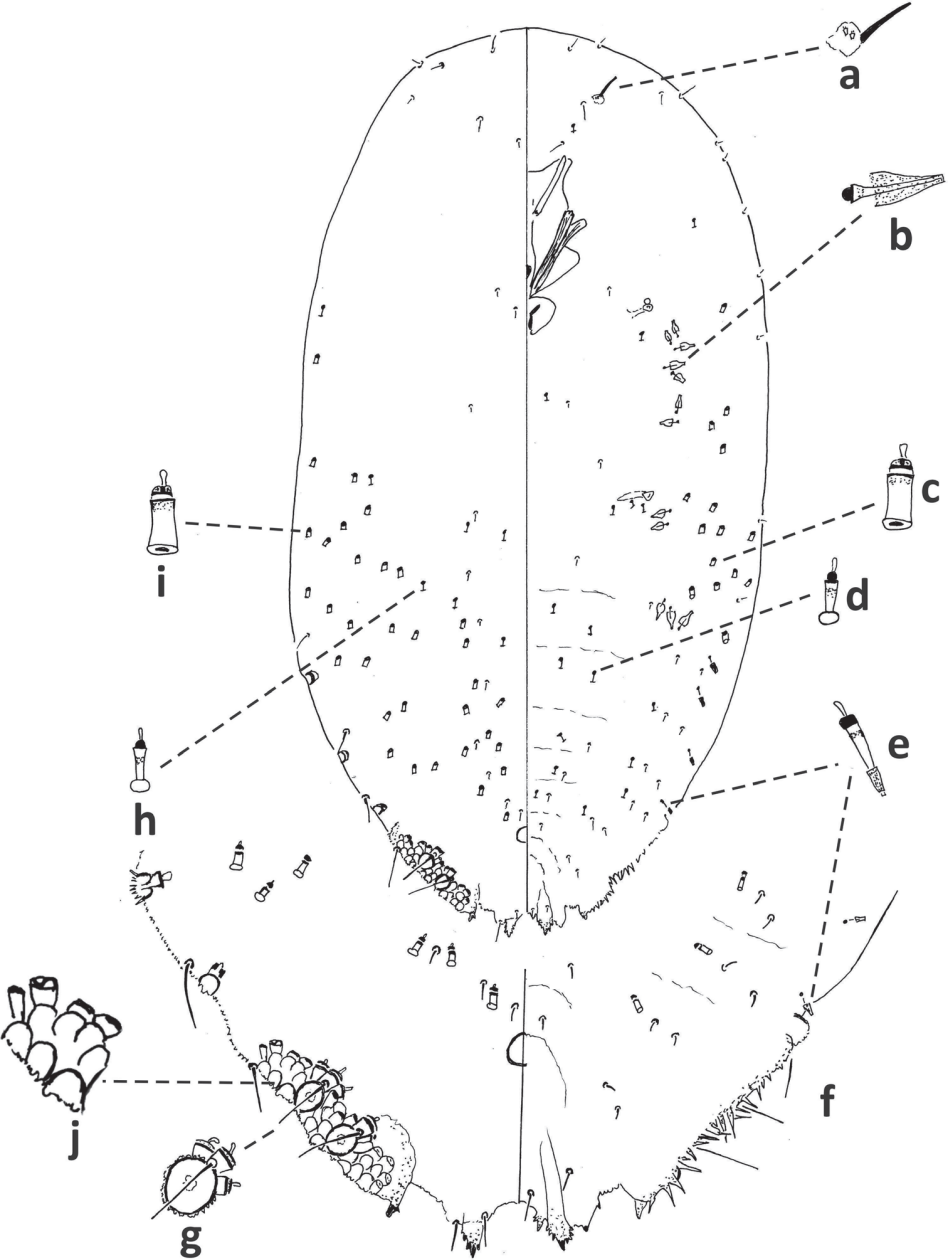
*Fioriniapinicola*, second-instar male, Hong Kong, China, December 1895, on *Pinussinensis*, A. Koebele (1529) mounted from type material. Abbreviations: a) antenna; b) large gland spine; c) large microduct; d) small microduct; e) small gland spine; f) enlargement of pygidium; g) enlargement of communal duct ; h) small microduct; i) large microduct; j) enlargement of part of duct cluster.

##### Notes.

We have been unable to find characters that consistently separate second-instar females of *F.japonica* and *F.pinicola*. The swelling of the body adjacent to the abdominal macroducts is usually pointed in *F.pinicola* and is usually rounded in *F.japonica*, but we have too few specimens to understand the possible variation in this character.

##### Second-instar male.

One duct cluster on each side of body, composed of several small ducts and two communal ducts. Three longitudinal lines of microducts on venter of abdomen (one medial and two submarginal). Cluster of small microducts with sclerotized orifice laterad of anterior spiracle absent. Five or more gland spines on each side of body between anterior and posterior spiracles. Antennae each with one enlarged seta.

##### Specimens examined for description and diagnosis.

China, Hong Kong, December 1895, on *Pinussinensis*, A. Koebele 2^nd^ ♀, ♂ (1529); United States, California, Los Angeles Co., Los Angeles, August 11, 2020, on *Podocarpusmacrophyllus*, N. Ellenrieder, 3 2^nd^ ♀, 2 ad ♀ (2020-3174).

##### Other specimens examined from USNM.

China, Hong Kong, December 1895, on *Pinussinensis*, A. Koebele (1529) mounted from type material, 2 2^nd^ ♀, 2 2^nd^ ♂, 2 ad ♀; Japan, Yokohama, Yamashita-cho, October 15, 1941, on *Pittosporumtobira*, K. Soto 1 1^st^ ♀, 2 2^nd^ ♀ (Yokohama 199); Japan, November 2, 1977, on *Podocarpus* sp., 1 1^st^ ♀, 7 ad ♀; United States, California, Orange Co., October 2002, on *Pittosporum* sp., H. Mitchell 1 1^st^ ♂ embryo, 1 1^st^ ♀ embryo, 7 2^nd^ ♀, 6 ad ♀.

#### Fiorinia proboscidaria

Taxon classificationAnimaliaHemipteraDiaspididae

Green, 1900

8F18EAE1-24E1-582B-853E-E317E0408028

##### Field characteristics.

First-instar exuviae overlapping second-instar exuviae. Without indentation formed between attachment of first- and second-instar exuviae. Second-instar exuviae oval, convex marginally or parallel sided; light to medium dark brown; longitudinal ridge conspicuous and thick. Posterior end of adult female within second-instar exuviae rounded. Heavily infested leaves with white secretion.

##### First-instar nymph.

Similar to *F.fioriniae* and *F.phantasma* in having gland spines on abdominal segment VI at least half as long as gland spine on segment VII. *Fioriniafioriniae* differs by having (characters in parentheses are those of *P.proboscidaria*): inner apex of large duct on head flat (rounded or mushroom like). *Fioriniaphantasma* differs by having (characters in parentheses are those of *F.proboscidaria*): pattern of derm surrounding large duct on head globular (serpentine); inner apex of large duct on head rounded or mushroom like (flat).

##### Adult female.

Process between antennae without spicules, often clubbed. Head conical. Antennae close together. Macroducts usually 3–5 on each side of pygidium, thin, longer than wide, resembling microducts. Gland spines barely projecting from body margin. Gland tubercles nearly continuous along body margin from head to abdominal segment III. Microducts in medial areas of prepygidial segments dorsally and ventrally. Lateral margin of head with cluster of circular tubercles possibly representing eye.

##### Notes.

There are a number of species with processes between the antennae; Wei et al. (2013) included 16 species in their key to the *Fiorinia* species from China. Only a few have an unusually elongate interantennal process and a conical head. *Fioriniaproboscidaria* resembles *F.biakana* Williams and Watson but differs by (characters in parentheses are those of *F.biakana*): space between median lobes less than width of median lobe (greater than width of lobe); macroducts ca. same width as gland spine ducts (wider than gland spine ducts); gland spines slightly protruding from derm surface (protruding at least half length of gland spine duct); gland tubercles continuous along body margin (grouped in clusters). This species also resembles *F.turpiniae* Takahashi but differs by (characters in parentheses are those of *F.turpiniae*): trilocular pores present near the anterior spiracle (absent); gland spines short, shorter than gland spine duct (long, longer than gland spine duct). *Fioriniaproboscidaria* differs from *F.randiae* Takahashi by (characters in parentheses are those of *F.randiae*): gland spines short, shorter than gland spine duct (long, longer than gland spine duct); median lobes nearly parallel (divergent). Florida specimens of *F.proboscidaria* are consistent with the description of Williams and Watson (1988) and Takagi (1970) except that both illustrated a lobe on each side of the head (Florida specimens lack these lobes), and that neither described the cluster of circular tubercles near the margin of the head or the ventromedial microducts anteriad of the pygidium. The illustration of Takagi has small lines at the end of the lobe on the side of the head which may be the same as the circular tubercles mentioned above, but they were not discussed in the description.

##### Second-instar female.

Median lobes broad, as wide as or wider than medial lobule of second lobe, projecting ca. same amount or slightly less than medial lobule of second lobes. With four pairs of marginal macroducts. Swelling of body margin adjacent to macroduct usually rounded. With three large gland spines on margin of each side of body from abdominal segments II–IV; usually with small gland spine on each side of abdominal segments V and VI; without small gland spines on abdominal segment I. With three microducts on each side of head. Longitudinal line of microducts present submarginally on venter of abdominal segments II–VI, normally with 1–5 microducts on each side of each segment. Small lobular projections on anterior of head sometimes present. Cicatrix present on dorsal submargin of abdominal segment I.

##### Second-instar male.

Two duct clusters on each side of body, anterior cluster without communal duct, posterior cluster composed of communal duct without associated smaller ducts. Normally, three longitudinal lines of microducts on venter of abdomen (one medial and two submarginal) occasionally with two medially forming four longitudinal lines. Without cluster of small microducts with sclerotized orifice laterad of anterior spiracle. Fewer than five gland spines on each side of body between anterior and posterior spiracles. Antennae each with one enlarged seta.

**Figure 16.**
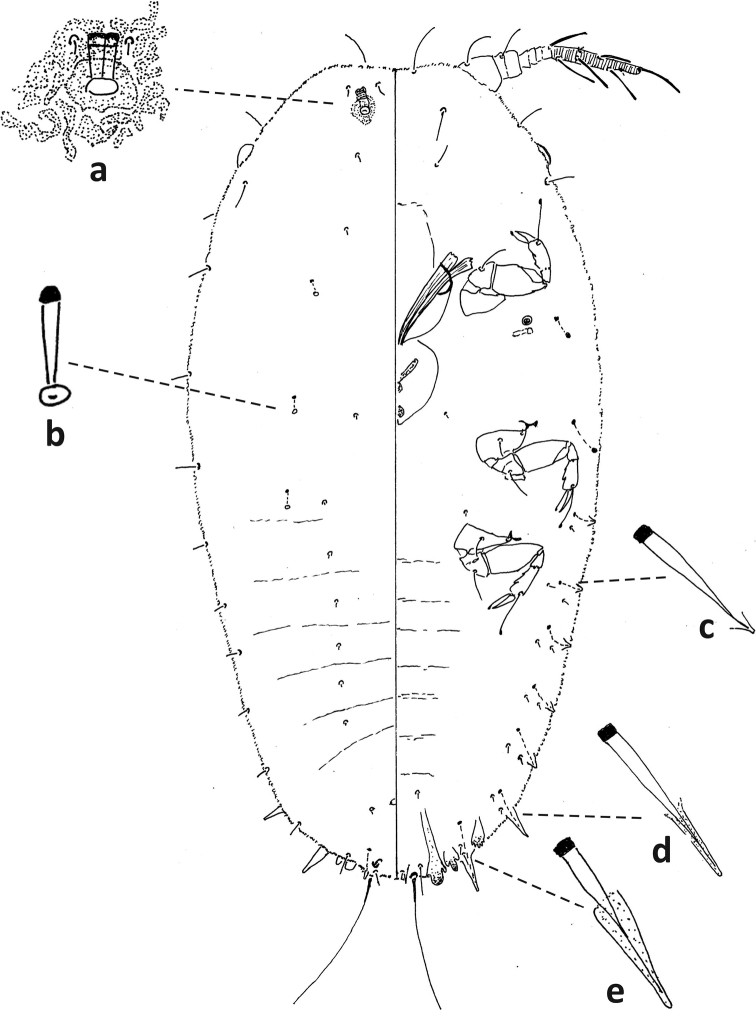
*Fioriniaproboscidaria*, First-instar nymph, Putnam Co., Crescent City, October 2, 2019, on *Citrus* sp., D. Rigby, M. Cain, (2018-5548). Abbreviations: a) large duct on head; b) dorsal microduct; c) short gland spine on abdominal segment II; d) long gland spine on abdominal segment VI; e) long gland spine on abdominal segment VII.

**Figure 17.**
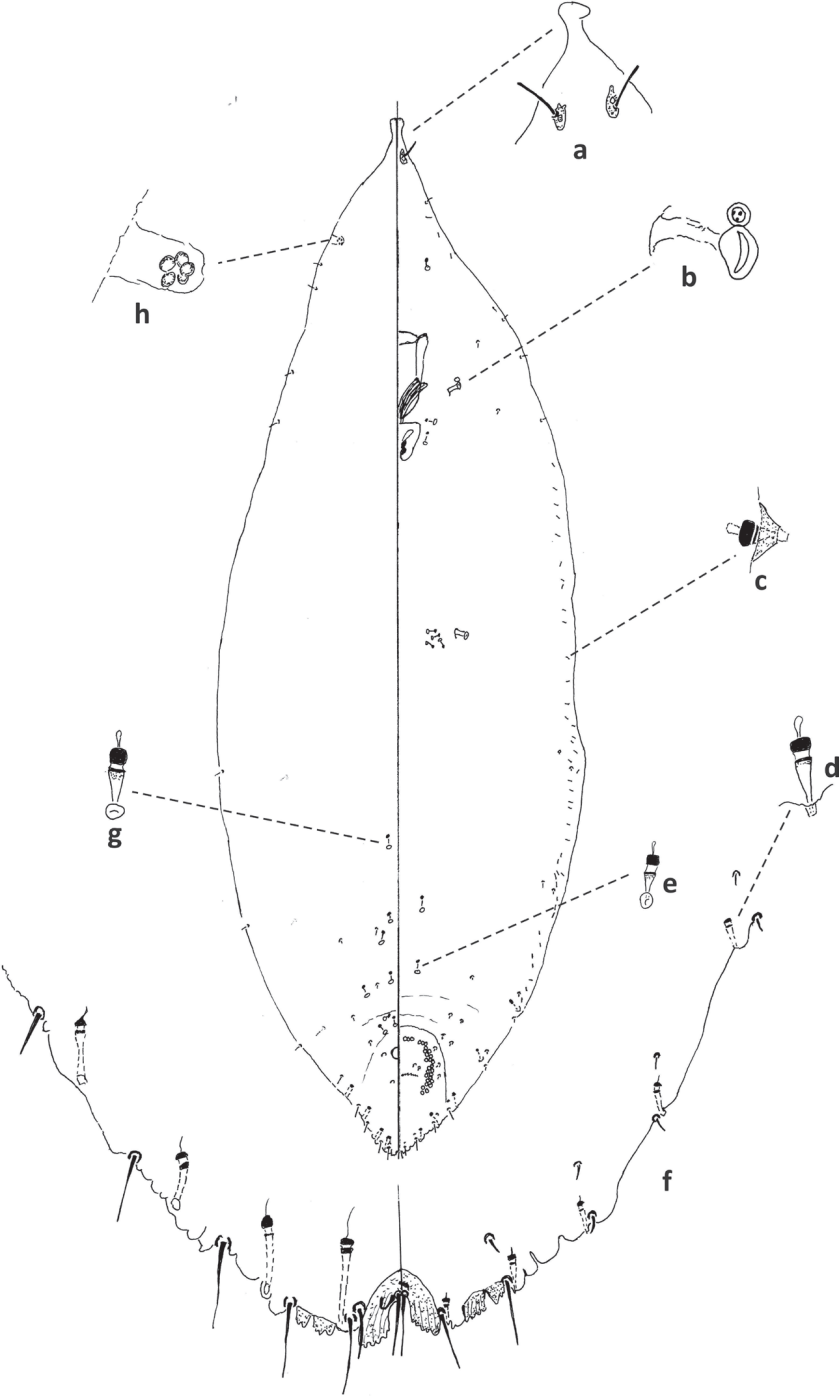
*Fioriniaproboscidaria*, adult female, Hillsborough Co., Tampa, December 16, 2013, on *Citrus* sp., J. Hoffman (2013-9087). Abbreviations: a) conical head with protrusion, antennae: b) anterior spiracle; c) gland tubercle; d) gland spine with small dermal protrusion; e) small microduct; f) enlargement of pygidium; g) microduct; h) circular tubercles in invagination.

**Figure 18.**
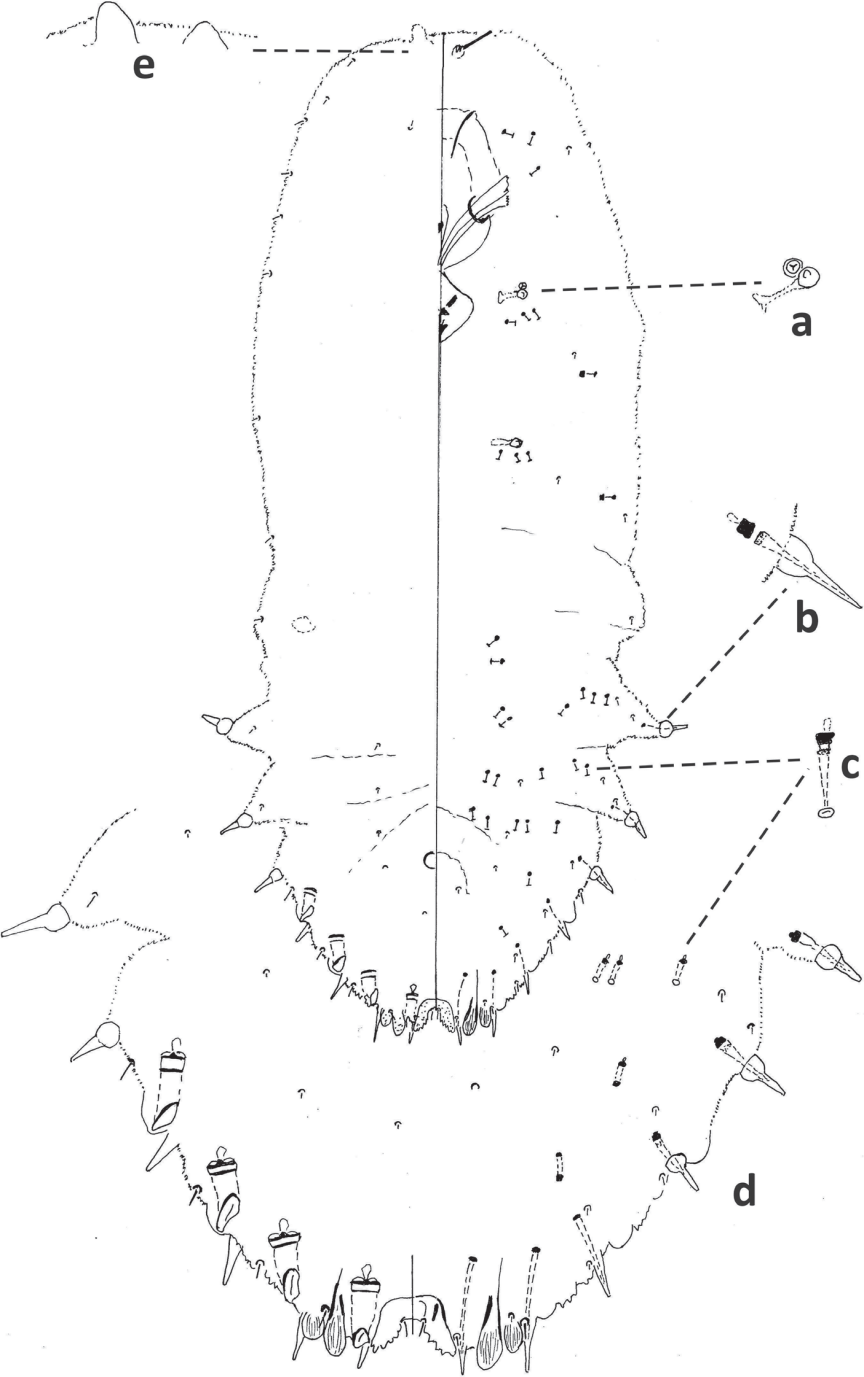
*Fioriniaproboscidaria*, second-instar female, Flagler Co., Palm Coast, June 18, 2020, on *Citrus* sp., M. Cain, (2020-2353). Abbreviations: a) anterior spiracle; b) large gland spine, c) small microduct; d) enlargement of pygidium; e) lobular projections on head.

**Figure 19.**
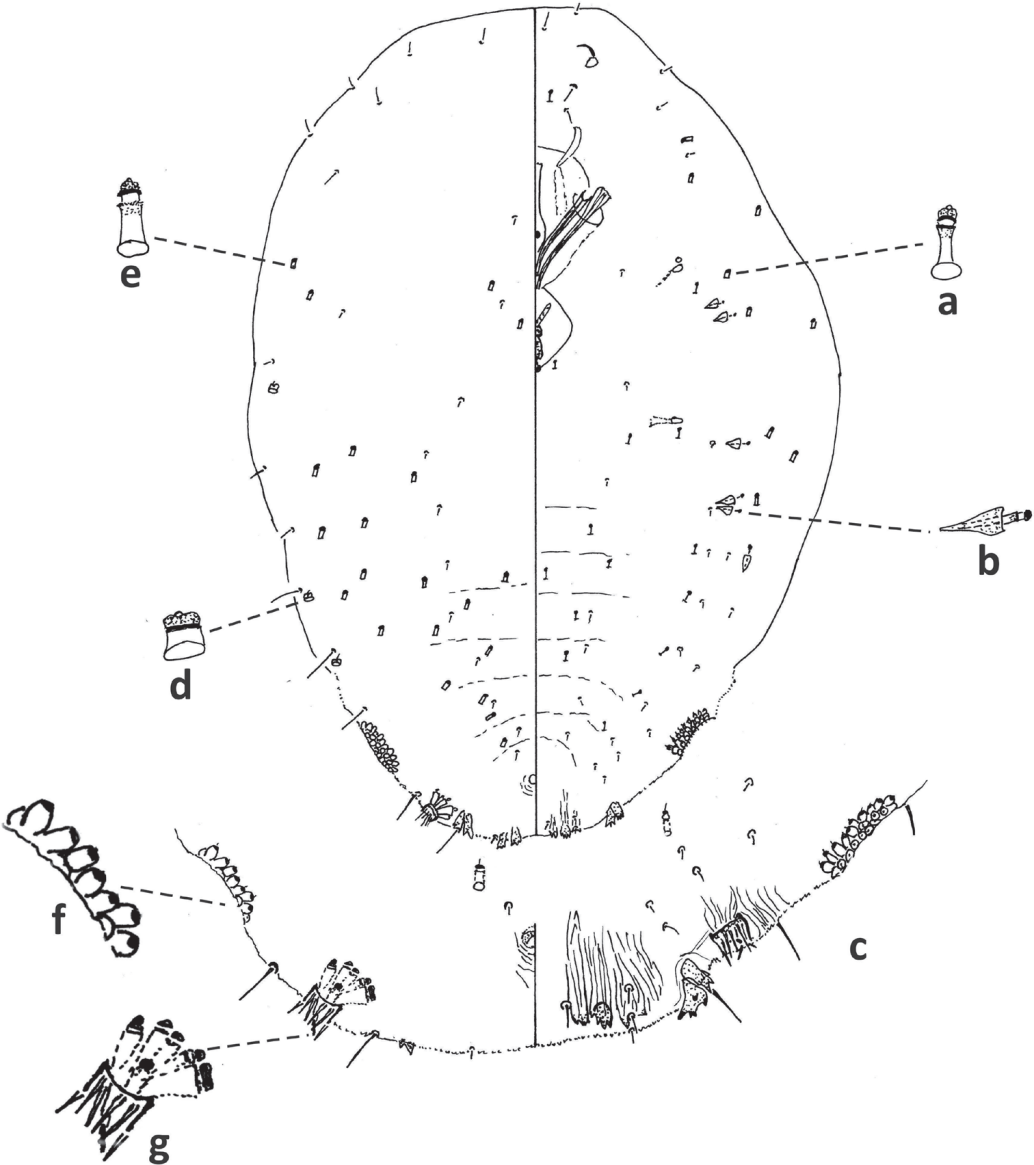
*Fioriniaproboscidaria*, second-instar male, Putnam Co., Crescent City, October 2, 2019, on *Citrus* sp., D. Rigby, M. Cain, (2018-5548). Abbreviations: a) small microduct; b) large gland spine; c) enlargement of pygidium; d) small macroduct; e) large microduct; f) enlargement of part of duct cluster; g) enlargement of communal duct.

##### Florida collection records.

Hillsborough Co., Tampa, December 16, 2013, on *Citrus* sp., J. Hoffman (2013-9087) (7 slides); Hillsborough Co., Tampa, November 19, 2014, on *Citrus* sp., M. Briceno (2014-859) (6 slides); Hillsborough Co., Tampa, October 23, 2014, on *Citrus* sp., M. Briceno (2014-7431) (2 slides); Hillsborough Co., Tampa, October 30, 2014, on *Citrus* sp., M. Briceno (2014-7574) (7 Slides); Hillsborough Co., Valrico, September 17, 2018, on Citrus×paradisi, P. Barker (2018-4907) (2 slides); Putnam Co., Palatka, October 25, 2018, on *Ilexcornuta*, M. Cain, T. Wright, and C. Hall (2018-5664) (2 slides); Santa Rosa Co., Gulf Breeze, January 3, 2014, on *Citrus* sp., M. Anderson (2014-46) (3 slides).

##### Specimens examined for description and diagnosis.

Flagler Co., Palm Coast, June 18, 2020, on *Citrus* sp., M. Cain, 5 2^nd^ ♀, (2020-2353); Hillsborough Co., Tampa, December 16, 2013, on *Citrus* sp., J. Hoffman 5 ad ♀ (2013-9087); Manatee Co., Bradenton, January 6, 2021, on *Citrus* sp., P. Kumar, 10 ad ♀ (2021-67); Pinellas Co., Palm Harbor, September 16, 2019, on *Citrus* sp. B. Rose, 2 ad ♀ (2019-5124); Putnam Co., Crescent City, October 2, 2019, on *Citrus* sp., D. Rigby, M. Cain, 5 1^st^ (2018-5548); Putnam Co., Crescent City, October 2, 2019, on *Citrus* sp., D. Rigby, M. Cain, 5 2^nd^ ♂ (2018-5548).

##### Material examined from USNM.

China, Hong Kong, September 2, 1980, on *Podocarpus* sp., J. Dooley 1 2^nd^ ♂, 1 prepupa, 4 ad ♀ (Los Angeles 25002). Martinique, February 8, 2000, on *Citrusaurantifolia*, K. Stewart 1 2^nd^ ♀, 1 ad ♀ (St. Thomas 010770).

#### Fiorinia theae

Taxon classificationAnimaliaHemipteraDiaspididae

Green, 1900

06798ADC-EED1-54A6-AEF2-4DB1C23C42BC

##### Field characteristics.

First-instar exuviae overlapping second-instar exuviae. Without indentation between attachment of first- and second-instar exuviae. Second-instar exuviae oval, convex marginally; light gray to nearly black; longitudinal ridge conspicuous. Posterior end of adult female within second-instar exuviae rounded. Heavily infested leaves with extensive white secretion (Suppl. material 1: Fig. S1).

##### First-instar nymph.

Described in Howell (1977).

##### Second-instar female.

Median lobes broad, equal to or wider than width of medial lobule of second lobe, projecting ca. same amount or slightly less than medial lobule of second lobes. With four pairs of marginal macroducts. Swelling of body margin adjacent to macroduct usually rounded. With three large gland spines on margin of each side of body from abdominal segments II–IV; usually with small gland spine on each side of abdominal segments V and VI; without small gland spines on abdominal segment I. With three microducts on each side of head. Longitudinal line of microducts present submarginally on venter of abdominal segments II–VI, normally with 1–5 microducts on each side of each segment. Small lobular projections anteriorly on head sometimes present. Cicatrix present on dorsal submargin of abdominal segment I.

##### Notes.

We have been unable to find characters that consistently separate second-instar females of *F.proboscidaria* and *F.theae*.

##### Second-instar male.

Two duct clusters on each side of body, anterior cluster without communal duct, posterior cluster composed of two communal ducts without associated smaller ducts. Five longitudinal lines of microducts on venter of abdomen (one medial, two mediolateral and two submarginal). Without cluster of small microducts with sclerotized orifice laterad of anterior spiracle (sometimes with one duct present). Fewer than five gland spines on each side of body between anterior and posterior spiracles. Antennae each with one enlarged seta. Some specimens with small protrusions that are remnants of legs.

##### Florida collection records.

Alachua Co., Alachua, October 1, 2012, *Camellia* sp., C. Jones, (2012-7479) (2 slides); Alachua Co., Gainesville, February 10, 1965, on *Camelliajaponica*, A.E. Graham (1965-0345); Alachua Co., Gainesville, October 16, 1973, on *Citrusmitis*, F. Collins (1973-3065); Alachua Co., Gainesville, October 25, 1979, on *Citrus* sp., R.I. Sailer (1979-1513); Alachua Co., Gainesville, March 20, 1991, on *Ilex* sp., F. Bennet (1991-2953); Alachua Co., Gainesville, March 20, 1991, on *Ilex* sp., F. Bennett (1991-001–002) (2 slides); Alachua Co., Gainesville, July 19, 1991, on *Ilex* sp., F. Bennett (1991-0383) (3 Slides); Alachua Co., Gainesville, December 3, 1991, on *Ilex* sp., F. Bennet (1991-002–003) (2 slides); Alachua Co., Gainesville, October 16, 1992, *Ilex* sp., F. Bennett (1992-001); Alachua Co., Gainesville, April 15, 1999, on *Ilex* sp., D. Strosnider (1999-383) (2 slides); Alachua Co., Gainesville, July 2011, on *Ilexcornuta*, Shirley Vogel (2011-4847); Alachua Co., Gainesville, February 2012, on *Camelliasasanqua*, D. Feiber (2012-1006) (3 slides); Alachua Co., Gainesville, January 8, 2013, on *Illiciumfloridanum*, M. Frank (2013-102) (2 slides); Alachua Co., Gainesville, December 1, 2013, on *Camellia* sp., T. Harris (2013-8692); Alachua Co., Hawthorne, February 26, 1971, on *Aucubajaponica*, E.W. Holder (1971-2976) (5 slides); Baker Co., Macclenny, March 14, 1968, on *Ilexlatifolia*, H.W. Collins (1968-351) (2 slides); Baker Co., Macclenny, October 21, 1975, on *Ilexcornuta*, C. Webb (1975-411); Bay Co., Panama City, March 8, 1978, on *Euonymus*sp., A.E. Graham (1978-003); Brevard Co., Grant, June 15, 1962, on *Camellia* sp., H.C. Levan (1962-0388); Citrus Co., Hernando, August 15, 1979, on *Ilexcornuta*, R.H. Phillips (1979-1604) (2 slides); Collier Co., Naples, November 13, 2012, on *Ilex* sp., S. Krueger (2012-8615) (2 slides); Collier Co., Naples, November 13, 2012, on *Ilex* sp., S. Krueger (2012-8618) (2 slides); Dixie Co., Old Town, November 19, 1979, on *Euonymus* sp., F. McHenry (1979-1664) (3 slides); Dixie Co., Suwannee, May 22, 1978, on *Citrusnobilis*, A.E. Graham and A. Hamon (1978-1601) (3 slides); Duval Co., Jacksonville, February 17, 1981, on *Ilexcassine*, H. Collins (1981-0352) (2 slides); Duval Co., Jacksonville, October 13, 1981, on *Citrussinensis*, G. Virgona (1981-0460, 3056) (3 slides); Duval Co., Jacksonville, January 25, 2005, on *Camelliajaponica*, J. Smith (2005-464); Duval Co., Jacksonville, October 26, 2010, on *Ilexvomitoria*, J. Brambila (2010-6593); Duval Co., Jacksonville, October 15, 2012, on *Camellia* sp., K. Theriault (2012-7841–7842) (4 slides); Duval Co., Jacksonville, October 25, 2012, on *Citrus* sp., K.Coffey, Lisa Hassell (2012-8102) (2 slides); Duval Co., Jacksonville, April 15, 2013, on *Ilex* sp., K. Theriault (2013-2501) (2 slides); Duval Co., Jacksonville, March 26, 2014, on *Camelliasasanqua*, L. Hassel (2014-2025) (2 slides); Escambia Co., Pensacola, March 10, 1991, on *Camellia* sp., F.D. Bennett (1991-0425, 3010) (2 slides); Flagler Co., Bunnell, January 11, 2012, on *Ilexcornuta* (2012-369) (4 slides); Gadsden Co., Chattahoochee, December 13, 1990, on *Camellia* sp., F. Bennett (1990-011–013) (3 slides); Gadsden Co., Quincy, January 28, 2005, on *Ilex* sp., B. Cecil (2005-901); Gadsden Co., Quincy, March 20, 2012, on *Poncirus* sp., M. Bentley (2012-1944) (2 slides); Hillsborough Co., Brandon, May 29, 1986, on *Ilex* sp., J. Felty (1986-0453); Hillsborough Co., Tampa, March 13, 2018, on Citrus×paradisi, M. Briceno (2018-1035) (3 slides); Indian River Co., Vero Beach, August 26, 2013, on *Ilex* sp., J. Kennedy (2013-6273); Jefferson Co., Monticello, November 29, 1973, on *Citrus* sp., W.H. Pierce (19730348) (2 slides); Lake Co., Clermont, February 7, 2012, on *Camellia* sp., H. Alred (2012-793) (3 slides); Lake Co., Eustis, January 22, 1965, on *Camellia* sp., A.L. Bentley (1965-1655) (4 slides); Lake Co., Eustis, April 29, 2010, on *Ilex* sp., M. Sellers (2010-2438) (2 slides); Lake Co., Eustis, May 3, 2018, on *Camelliajaponica*, M. Sellers (2018-2334) (2 slides); Leon Co., Tallahassee October 13, 1919, on *Camelliajaponica*, P.F. Robertson (1919-1619) (2 Slides); Leon Co., Tallahassee, August 8, 1976, on Citrus×paradisi, S. Beidler (1976-0423) (3 slides); Leon Co., Tallahassee, October 2, 1978, on *Euonymus* sp., Q. Anglin (1978-3073) (3 slides); Leon Co., Tallahassee, December 13, 1991, on *Camelliajaponica*, F. Bennett (1991-0445) (3 slides); Leon Co., Tallahassee, April 6, 2015, on *Camellia* sp., M. Bentley (2015-1687) (3 slides); Madison Co., Pinetta, October 22, 1985, on *Poncirustrifoliata*, J. Thomas (1985-1596); Manatee Co., Duette, March 2, 2005, on *Ilexcornuta*, K. Pippenger (2005-1077); Manatee Co., Oneco, October 19, 1923, on *Camelliajaponica*, D.F. Schwarts (1923-1594) (2 slides); Marion Co., Citra, March 2, 2007, on *Ilex* sp., F. McHenry (2007-1335); Marion Co., Citra, March 2, 2007, on *Ilex* sp., F. McHenry (2007-1335) (2 slides); Marion Co., November 15, 2010, on *Camellia* sp. (2010-7091); Marion Co., Reddick, November 15, 2010, on *Camellia* sp., S. Wayte (2010-7091); Martin Co., Jensen Beach, April 23, 2014, on *Ilexopaca*, L. West (2014-2807) (2 slides); Martin Co., Stuart, December 13, 1979, on *Raphiolepisumbellata*, R. Gaskalla (1979-2966) (3 slides); Miami-Dade Co., Miami, December 6, 2012, *Ilex* sp., O. Garcia (2012-9157) (2 slides); Miami-Dade Co., Surfside, March 1, 2018, on *Citrus* sp., O. Garcia (2018-788) (2 slides); Nassau Co., Fernandina Beach, November 7, 2017, on *Citrus* sp., R. Leahy (2017-4261) (2 slides); Nassau Co., Yulee, September 21, 2012, on *Ilex* sp., R. Traya (2012-7169) (2 slides); Nassau Co., Yulee, March 14, 2013, on *Ilexvomitoria*, R. Traya (2013-1586) (2 slides); Nassau Co., Yulee, July 25, 2016, on *Ilexcornuta*, R. Traya (2016-3604); Orange Co., April 30, 2002, on *Citrusreticulata*, L. Brown (2002-1644); Orange Co., Apopka, January 30, 1990, on *Ilexvomitoria*, C. Murphy (1990-0353) (4 slides); Orange Co., Apopka, November 17, 1998, on *Camelliajaponica*, L. Wilber (1998-3011) (5 slides); Orange Co., Apopka, April 20, 2002, on *Citrusreticulata*, L. Brown (2002-1644) (2 slides); Orange Co., Apopka, March 8, 2012, on *Ilex* sp., K. Gonzalez (2012-1575) (2 slides); Orange Co., Orlando, February 3, 1977, on *Camellia*sp., D.A. Graddy (1977-1514) (6 slides); Orange Co., Orlando, April 19, 2010, on *Citrusreticulata*, L. Russe (2010-2055); Orange Co., Orlando, February 16, 2012, on *Ilex* sp., R. Lopez (2012-1048) (3 slides); Orange Co., Orlando, February 2013, on Theaceae, A. Puppelo (2013-1127); Orange Co., Orlando, August 20, 2014, on *Camelliajaponica*, T. Lyons (2014-5856) (2 slides); Orange Co., Pine Castle, February 5, 1962, on *Camellia*sp., A.C. Crews (1962-0333) (2 slides); Orange Co., Maitland, July 30, 1970, on *Camelliajaponica*, E.R. Simmons (1970-1516) (2 slides); Palm Beach Co., West Palm Beach, October 9, 1979, on *Perseaamericana*, N. Miles (1979-029–032) (4 slides); Palm Beach Co., West Palm Beach, October 17, 2012, on *Citrus* sp., M. Clark (2012-7966) (2 slides); Pasco Co., Lutz, November 3, 2011, on *Citrussinensis*, L. Osbeck (2011-8420) (2 slides); Pasco Co., Odessa, December 21, 2011, on *Ilexcornuta* (2011-9392); Pinellas Co., Clearwater, November 15, 1962, on *Senecioconfusus*, C.E. Bingaman (1962-1623); Pinellas Co., Gulfport, December 14, 1977, on *Citrusaurantifolia*, K. Hickman (1977-0370) (2 slides); Pinellas Co., Oldsmar, May 5, 2005, on *Citrus* sp., D. Albritton (2005-2337); Pinellas Co., Palm Harbor, February 2012, on *Citrusmaxima*, G. Campani and J. Hawk (2012-1112) (3 slides); Pinellas Co., Palm Harbor, November 16, 2010, on *Citruslimon*, J. Brownstein (2010-7134); Pinellas Co., Safety Harbor, April 11, 2012, on *Syzygiumjambos*, L. Alston (2012-2672) (2 slides); Pinellas Co., St. Petersburg, May 4, 1979, on *Citrusreticulata*, K. Hickman (1979-0469) (2 slides); Pinellas Co., St. Petersburg, March 25, 2010, on *Illiciumfloridanum*, G. Bernard (2010-1506); Pinellas Co., Tarpon Springs, February 18, 2012, on *Citrus* sp., K. Edgerton (2012-1039) (2 slides); Pinellas Co., Tarpon Springs, March 3, 2015, on *Cinnamomumcamphora*, B. Rose (2015-928) (2 slides); Polk Co., Haines City, January 22, 2013, on *Ilex* sp., S, Distelberg (2013-0328) (3 slides); Polk Co., Winter Haven, December 12, 1960, on *Camelliajaponica*, A.C. McAulay and V.K. Norton (1960-0374); Polk Co., Winter Haven, September 9, 1963, on *Gardenia* sp., L.H. Heeb (1963-1653); Polk Co., Winter Haven, December 2, 2013, *Camellia* sp., C. Gibbard (2013-8729); Putnam Co., East Palaka, August 1, 2018, on *Citrus* sp., M. Cain (2018-4101) (2 slides); Putnam Co., Pomona Park, December 12, 1968, on *Euonymusamericanus*, A.E. Graham (1968-0454) (6 slides); Santa Rosa Co., Bagdad, January 29, 1970, on *Euryajaponica*, R.W. Albritton (1970-1654) (7 slides); Santa Rosa Co., March 5, 2012, on *Camelliajaponica*, M. Anderson (2012-1534); Seminole Co., Longwood, July 30, 1970, *Camelliajaponica*, E.R. Simmons (1970-3068); Seminole Co., Oviedo, January 31, 2013, on *Cleyerajaponica*, J. Krok (2013-640) (2 slides); Seminole Co., Oviedo, March 13, 2013, on *Ilexopaca*, J. Krok (2013-1691); Seminole Co., Sanford, July 16, 2012, on Myrtaceae, J. Krok, (2012-5308) (2 slides); St. Johns Co., St. Augustine, May 24, 2013, on *Ilex* sp., K. Theriault (2013-3667) (2 slides); St. Lucie Co., Ft. Pierce, February 21, 2005, on *Ilexcornuta*, D. Vazquez (2005-4069) (3 slides); Sumter Co., Bushnell, January 12, 2012, on *Camellia* sp., H. Alred (2012-377) (3 slides); Sumter Co., Bushnell, May 7, 2010, on *Camelliasasanqua*, H. Alred (2010-2501); Sumter Co., Center Hill, April 17, 2012, on *Camelliajaponica*, H. Alred (2012-2752) (4 slides); Suwannee Co., Brandford, November 22, 2010, on *Camelliajaponica*, W.W. Bailey (2010-7236); Suwannee Co., Live Oak, December 22, 2004, on *Camelliajaponica*, Wayne Bailey (2004-8133); Suwannee Co., Live Oak, February 19, 2008, on *Camelliajaponica*, W. Wayne Bailey (2008-209); Suwannee Co., Live Oak, February 28, 2012, on *Ilexcornuta*, D. Ruseell-Hughes and K. Collins (2012-1341) (3 slides); Suwannee Co., Live Oak, February 28, 2012, on *Ilexcornuta*, K. Collins (2012-1329) (2 slides); Suwannee Co., Live Oak, July 24, 2012, on Aquifoliaceae, D. Russell-Hughes (2012-5483) (2 slides); Suwannee Co., Live Oak, February 19, 2013, *Camellia* sp., W. Wayne Bailey (2013-1082); Taylor Co., November 18, 2010, on *Camelliajaponica*, W. Wayne Bailey (2010-7155); Taylor Co., Perry, March 6, 1978, on *Citruslimon*, Q. Anglin (1978-0397); Taylor Co., Perry, March 6, 1978, on *Citruslimon*, Q. Anglin (1978-034); Taylor Co., Perry, March 6, 1978, on *Citruslimon*, Q. Anglin (1978-035); Taylor Co., Perry, March 8, 1979, on *Euonymus*sp., Q. Anglin (1979-037); Taylor Co., Perry, March 8, 1979, on *Euonymus*sp., Q. Anglin (1979-1599); Taylor Co., Perry, February 7, 2007, on *Camelliajaponica*, Wayne Bailey (2007-748); Taylor Co., Perry, November 18, 2010, on *Ilexcornuta*, W.W. Bailey (2010-7155); Taylor Co., Steinhatchee, May 24, 2010, on *Ilexcornuta*, W. Wayne Bailey (2010-2993); Volusia Co., Daytona Beach, FL, March 14, 1963, on *Malpighia*sp., J.N. Pott (1963-2963) (2 slides); Volusia Co., Orange Mills, October 6, 1964, on *Fortunella*sp., A.E. Graham (1964-0470) (16 slides); Volusia Co., Holly Hill, November 16, 1971, on *Fortunella*sp., J.N. Pott (1971-0366) (2 slides); Volusia Co., Daytona Beach, November 23, 1976, on *Ilexcornuta*, J.N. Pott (1976-0378) (4 slides); Volusia Co., Ormond Beach, April 18, 2008, on *Ilexcornuta*, K. Coffey (2008-2272) (3 slides); Volusia Co., Edgewater January 13, 2012, on *Ilexcornuta* (2012-263) (3 slides).

**Figure 20.**
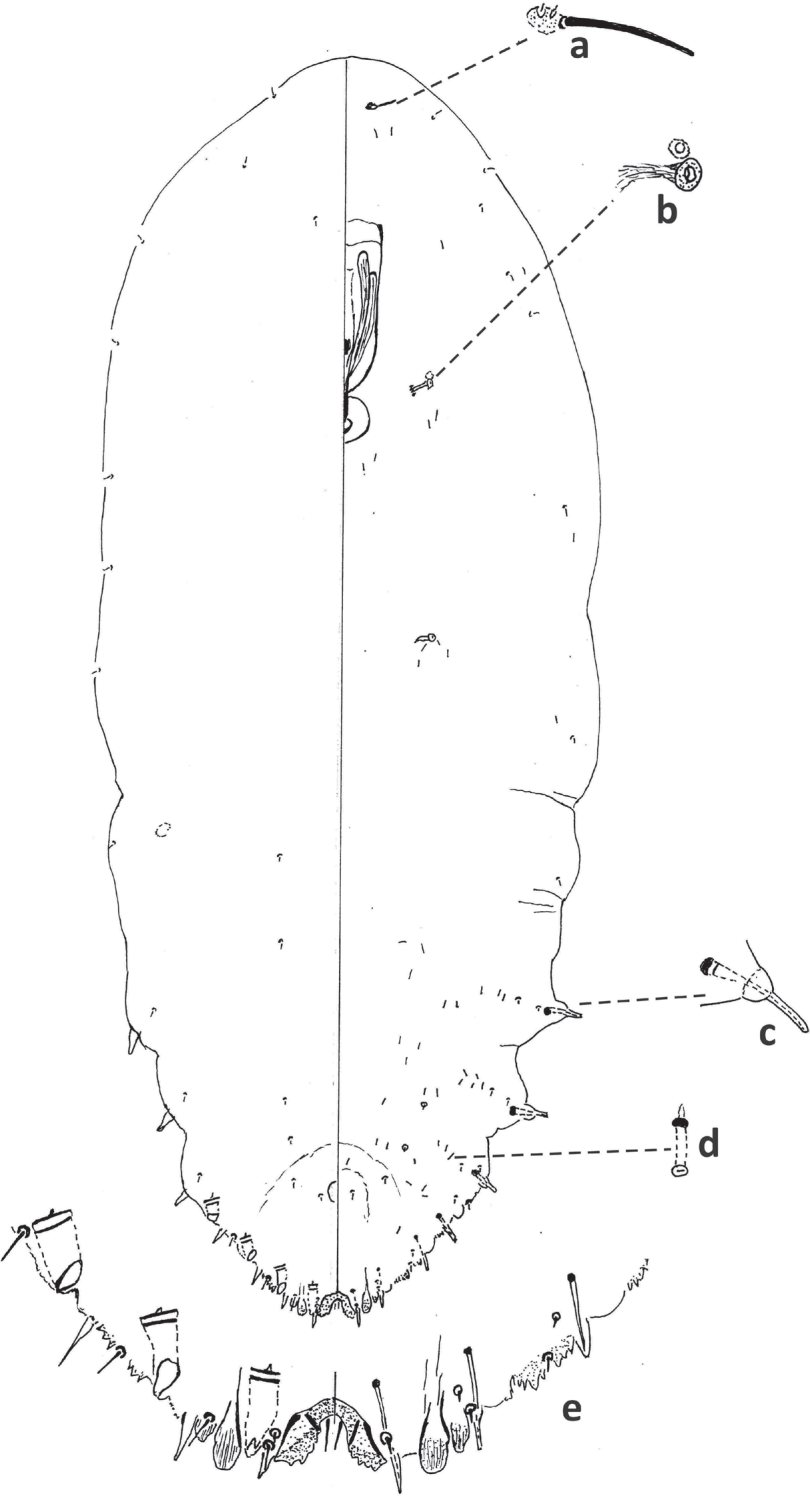
*Fioriniatheae*, second-instar female, Pulaski Co., Little Rock, Arkansas, February 15, 1972, on Bradford Holly. Abbreviations: a) antenna; b) anterior spiracle c) large gland spine; d) small microduct; e) enlargement of pygidium.

**Figure 21.**
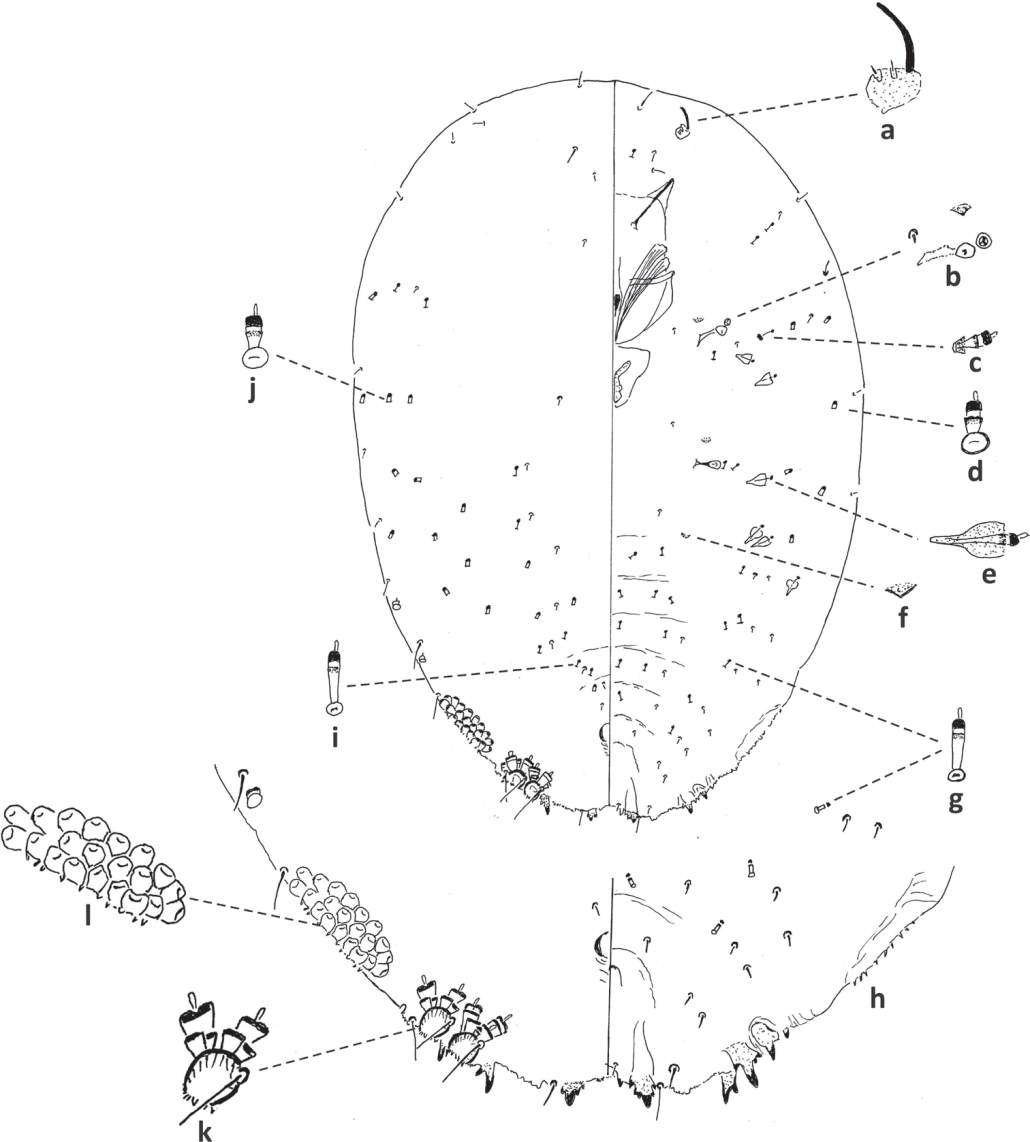
*Fioriniatheae*, second-instar male, Alachua Co., Gainesville, October 9, 2019, January 24, 2020 on *Ilex* sp., M. Borden, D. Miller (2019-5696, 2020-287). Abbreviations: a) antenna; b) anterior spiracle; c) small gland spine; d) large microduct; e) large gland spine; f) remnants of legs; g) small microduct; h) enlargement of pygidium; i) small microduct; j) large microduct; j) enlargement of part of duct cluster; k) enlargement of communal duct.

##### Specimens examined for description and diagnosis.

Little Rock, Arkansas, February 15, 1972, on Bradford Holly, 2^nd^ ♀; Alachua Co., Gainesville, October 9, 2019, January 24, 2020 on *Ilex* sp., M. Borden, D. Miller 5 2^nd^ ♂ (E2019-5696, E2020-287).

## Discussion

The most recent study providing taxonomic keys of first-instar nymphs of *Fiorinia* species was published more than four decades ago (Howell 1977). Our expanded version of this key includes the recently introduced species *F.phantasma* and *F.proboscidaria* and improves capabilities for the early detection of *Fiorinia* species in the USA. Our study, for the first time, generated COI barcodes of six *Fiorinia* species including *F.externa*, *F.fioriniae*, *F.phantasma*, *F.pinicola*, *F.proboscidaria*, and *F.theae*. One of the key taxonomic characters in the first-instar nymph key requires careful examination of gland spine morphology. However, gland spines can easily be damaged and are inconspicuous. Molecular identification of first-instar nymphs is recommended for *Fiorinia* species. First-instar nymphs soon molt to become second-instar nymphs (Beardsley and González 1975), and second-instar nymphs are easier to find in the field. We constructed, for the first time, a taxonomic key for second-instar females of the *Fiorinia* species occurring in the USA. Our second-instar female key successfully distinguishes three *Fiorinia* species: *F.fioriniae*, *F.externa*, and *F.phantasma*. The number of pairs of marginal macroducts were the same between *F.proboscidaria* and *F.theae*. Similarly, the ratio of the spaces between the bases of the median lobes versus the size of the medial lobule of second lobes was the same in *F.japonica* and *F.pinicola*. We suggest molecular sequencing of second-instar females for species-level identification. Contrary to second-instar females, our taxonomic key based on second-instar males distinguishes all seven *Fiorinia* species. The last taxonomic key for second-instar males of *Fiorinia* species was published ca. five decades ago by Tippins (1970) and included three *Fiorinia* species, *F.externa*, *F.pinicola*, and *F.theae*. We expanded upon it by including four more *Fiorinia* species, *F.fioriniae*, *F.japonica*, *F.phantasma*, and *F.proboscidaria*. Once a population of *Fiorinia* becomes established, adult females are usually available. They are easily observed and easier to slide-mount compared with immature stages. There are 16 *Fiorinia* species reported from the Australasian, Nearctic and Neotropical regions (Watson et al. 2015). Watson et al. (2015) provided a taxonomic key to the adult females of 12 of these species including the seven *Fiorinia* species used in this study. We developed a key to adult females that occurs in the USA modifying the key from Watson et al. (2015). Overall, the morpho-molecular diagnostic framework developed in this study will help identify first-instar nymphs, second-instar males and females, and adult females of *Fiorinia* species and will expediate regulatory and control decisions.

Use of immature armored scales for identification is hampered by the fact that slide mounting protocols are tedious and laborious. Immature stages, especially first-instar nymphs, are very small, ca. 0.1–0.2 mm in length, and can easily be lost during the mounting process. We reexamined previously published mounting protocols (McKenzie 1957; Wilkey 1990; Watson 2002) and addressed three issues: 1) avoiding specimen loss during mounting, 2) enhancing safety by reducing the amount of chemicals needed, since the reagents can be corrosive, flammable, carcinogenic, or produce toxic fumes, and 3) saving time if possible. Our comparative analysis of different slide-mounting protocols and elaboration on their merits and drawbacks, especially for the incorporation of a mesh container during the slide-mounting protocols, enhance the potential for mounting immature armored scales.

One unexpected discovery during this project was that the morphology of second-instar males was more reliable for species recognition than any other instar, including the adult female. For example, we were unable to distinguish between second-instar females of *F.proboscidaria* and *F.theae*, but their second-instar males were easily separated using the number of communal ducts. Second-instar males of *F.fioriniae* are remarkably different from the same instar of all other species of *Fiorinia* found in the USA even though other instars are quite similar to one another. Takagi (1975) discussed having difficulty separating *F.nachiensis* Takahashi and *F.odaiensis* Takagi based on adult females. At one point he treated them as synonyms, but based on major differences between the second-instar males he concluded that they were different species. Tippins (1970) published the first key and descriptions of the second-instar males of *Fiorinia* species and was surprised by the distinctive differences among species.

Recently, Liu et al. (2020) described a new species, *F.yongxingensis* from Hainan, China. It is similar to *F.phantasma* in the number and size of the marginal macroducts, the shape of the lobes, and the shape of the pygidium. The authors based their diagnosis in part on the detailed description and illustration of *F.coronata* (Williams & Watson, 1988), a junior synonym of *F.phantasma* (see Watson et al. 2015). Characters that appeared to be diagnostic for *F.yongxingensis* compared with *F.coronata* (= *F.phantasma*) are gland tubercles on the prothorax, microducts between the posterior spiracles, a gland spine on the prepygidium, and 0–3 pores near each anterior spiracle. Unfortunately, the type series of *F.coronata* did not contain the variation that we discovered in the Florida populations of *F.phantasma*. We have seen material with or without gland tubercles on the prothorax, a gland spine on the prepygidium, and 0–3 pores near each anterior spiracle. All specimens in the Florida populations have microducts between the posterior spiracles. Based on this information it appeared that the presence of these microducts was the key diagnostic character for *F.yongxingensis*. Because we needed to know the correct identity of the species introduced to Florida, several more steps were required. The next step was to examine the type series of *F.phantasma* and *F.coronata*. DL and JF borrowed the type specimens of *F.phantasma* from NHMUK and DRM examined another specimen from the type series deposited in USNM, but in each case the specimens were in such poor condition that it was impossible to see if microducts are present between the posterior spiracles. Type material of *F.coronata* also was studied; a type specimen deposited in the USNM has microducts between the posterior spiracles. A further step was to examine other relevant slides in the USNM. We studied slides from thirteen *F.phantasma* populations taken in quarantine from the Philippines, the type locality of *F.phantasma*, between 1965 and 1996, that are deposited in the USNM. We also examined slides taken in quarantine from Grenada, Hawaii, Thailand, Taiwan, and Vietnam. In all cases, microducts were present between the posterior spiracles, and there was overlapping variation in the other characters used to diagnose *F.yongxingensis*. We have yet to examine any adult female specimens of *F.phantasma* that lack these microducts and conclude that they are most likely a fixed character of the species.

The final step was to compare the results of multigene molecular analyses of the Florida population, the Chinese population, and two Malaysian populations (D1184 and D1185). The results clearly show that these populations are the same species. The morphological differences suggested as diagnostic of *F.yongxingensis* are within the range of variation that occurs in *F.phantasma*. Therefore, we here treat *F.yongxingensis* as a junior synonym of *F.phantasma*.

We obtained 37 5’-COI barcodes representing nine *Fiorinia* species in this study. Overall, low intraspecific genetic distances and high interspecific genetic distances ranging from 9.1% to 15.2% between *Fiorinia* species emphasize the reliability of 5’-COI barcodes in molecular diagnostics of armored scale species. Our rapid slide-mounting protocol and the morphological keys to immatures and adults can provide time- and cost-effective diagnostics of *Fiorinia* species in the USA. However, for instances where specimens are damaged and cannot be mounted and where molecular diagnostics is the only option, barcodes will help to identify the species of *Fiorinia*. All of our DNA extractions are vouchered by permanently archived specimens in FSCA. This provides the opportunity for other researchers to validate the identifications of our specimens. We found an example of apparently misidentified specimens that were submitted to Genbank: the barcode of *Aulacaspisrosarum* Borchsenius (isolate wfsys017, accession number KP981086) was placed with 35 samples of *Pseudaulacaspiscockerelli* (Cooley) in our molecular analysis. A subtler discrepancy between DNA sequence and morphological identification, also seen in Normark et al. (2019), is the placement of *F.vacciniae* Kuwana (isolate D2453A, accession number KY219617) together with three samples of *F.hymenanthis* Takagi. Our study accentuates the importance of depositing morphological voucher specimen in an accessible collection.

Three populations of *Fiorinia* species (isolates D4674F, D4778A, and D4682A), collected from Lambir Hills National Park, Malaysia, September 26, 2013 from an undetermined host, identified as *F.phantasma* by BBN, were found to be genetically different from the *F.phantasma* populations from China, Florida and Malaysia. We reexamined the skins of the specimens used in our molecular analyses. The slides of isolates D4674F and D4778A are in poor condition and covered with fog, but we can see processes between the antennae and the shape of the pygidium, and they are consistent with the morphology of *F.phantasma*. The slide of isolate D4682A appears to have most characters of *F.phantasma* including the microducts between the posterior spiracles. This isolate is ca. 9% genetically distant from *F.phantasma* (based on COI) and is placed far from the subclade of *F.phantasma* (containing populations from China, Florida, and Malaysia) in the concatenated phylogenetic tree (Fig. 2). This may represent a cryptic species. More samples especially of second-instar males would help to confirm their identity.

Recently phylogenetic analyses in Normark et al. (2019) support the monophyly *Fiorinia* after the generic transfer of *Ichthyaspisficicola* into the group. Our analysis agrees with the inference of Normark et al. (2019) with a few exceptions. Three *Pseudaulacaspis* MacGillivray species including *P.cockerelli*, *P.pentagona* (Targioni Tozzetti) and *P.prunicola* (Maskell) are placed in the same clade as *Fiorinia* in the case of the 5’-COI phylogenetic tree (Fig. 3, Suppl. material 1: Fig. S4). Likewise, in the case of the concatenated phylogenetic tree based on 28S, EF1-α, 5’-COI, 3’-COI, and COII, two samples of *Fiorinia* sp. (isolates D4815B, D4815C) fall out of the *Fiorinia* clade and placed with five *Pseudaulacaspis* species including *P.biformis* Takagi, *P.cockerelli*, *P.momi* (Kuwana), *P.pentagona*, and *P.prunicola*, with strong clade support (Fig. 2, Suppl. material 1: Fig. S2). *Fiorinia* was rendered polyphyletic by these two isolates (*Fiorinia* sp., D4815B and D4815C). They were collected from Malaysia in 2013 and determined as *Fiorinia* sp. by BBN. Given that the pupillarial habit has been gained and lost frequently in the history of Diaspididae (Normark et al. 2019), a second origin within Fioriniina would not be surprising. These two *Fiorinia* sp. isolates along with five *Pseudaulacaspis* species were placed together with strong support in a subclade within *Fiorinia* clade in our phylogenetic analysis based on 28S gene (Suppl. material 1: Fig. S3). Therefore, the placement of these two samples out of *Fiorinia* clade could be the result of an artifact of missing data or the methodology used in multigene tree and would require additional analysis for further confirmation. There are two samples of *Lineaspisstriata* (Newstead) with *P.simplex* Takagi in the sister subclade that joins the subclade of *Pseudaulacaspis*/*Fiorinia* with strong clade support (Fig. 3, Suppl. material 1: Fig. S4). Overall, the main clade of the genus *Fiorinia* joins the *Fiorinia*/ *Pseudaulacaspis*/ *Lineaspis* clade with strong clade support (> 90%). Borchsenius (1966) separated *Fiorinia* from *Pseudaulacaspis* and placed them in different tribes due to their pupillarial habit. However, Takagi (1969) and Howell and Tippins (1973), based on the presence of communal ducts, suggested a relationship between *Pseudaulacaspis* and *Fiorinia*. Our phylogenetic analysis suggests that additional sampling of *Fiorinia* and *Pseudaulacaspis* from Asia will further clarify the monophyly of the genus *Fiorinia*.

Field habitus of adult females, especially the character of the overlap between the first-instar and second-instar exuviae, was used for the first time in this study. For example, in the case of *F.externa*, the first-instar exuviae are barely touching the second-instar exuviae and form a distinct indentation between the attachment of the first- and second-instar exuviae (Suppl. material 1: Fig. S1). In contrast to this, no indentation was observed in *F.phantasma*. In addition, we also compared the color and shape of the second-instar nymphs shed skins of *Fiorinia* species. Field habitus can assist growers and nursery workers in making preliminary identifications.

*Fioriniajaponica* was eradicated from California and has been rediscovered three times since its first report in 1910 (Watson 2009). The most recent reinfestation was observed in 2008 and was most likely eradicated in a subsequent year (Watson 2009). Our collaborator’s attempt to collect fresh specimens of *F.japonica* in California for inclusion in this study was unsuccessful and its population has not been barcoded. It would be useful to trace its population in other states and to sequence its barcode. We also intended to include the population of *F.phantasma* from Hawaii, but efforts of our collaborators to collect it from Hawaii were unsuccessful. There have been at least two reinfestations of *F.phantasma* in Hawaii since its first report in 2004. The most recent heavy infestation was from palms reported in 2011 (Garcia 2011). Interestingly, in this most recent Hawaiian infestation, the second-instar nymph’s shed cuticles had transverse brown stripes, whereas the Florida population lacks this character. It would be helpful to collect *F.phantasma* from Hawaii and to compare it with the Floridian *F.phantasma* population to determine if they are the same species. If the Hawaiian *F.phantasma* is the same as the Floridian species, that fact might imply that *F.phantasma* in Florida could follow the same pattern as it did in Hawaii and keep reappearing with heavier infestations in subsequent years. This study will facilitate regulatory and pest management decisions by enhancing morphological and molecular identification of seven adventive *Fiorinia* species occurring in the USA.

## Conclusions

There are six main conclusions of our study. 1) The utilization of molecular barcodes is highly beneficial in diagnosing species of *Fiorinia* that occur in the USA. 2) The new keys in this study demonstrate that the USA species of *Fiorinia* can be identified using immature specimens. 3) Second-instar male morphology provided a reliable suite of characters for species-level identification. 4) Based on our comparative analysis of morphological characters and multigene molecular sequencing of specimens of *F.phantasma* and *F.yongxinensis*, it is clear that the latter is a junior synonym. 5) Of the different protocols tested for mounting immature specimens of *Fiorinia*, Hoyer’s mounting medium was the best for discerning delicate morphological characters but it was not desirable for permanent slide preparations. Balsam was the best for permanent mounts but did not provide the morphological clarity of Hoyer’s mounts. 6) The use of a mesh container in the process of mounting immatures is an effective method for preventing the loss of specimens. Overall, the use of the morphological and molecular data provides effective methods for early detection of new infestations and assists regulators in making control decisions.

## Supplementary Material

XML Treatment for Fiorinia externa

XML Treatment for Fiorinia fioriniae

XML Treatment for Fiorinia japonica

XML Treatment for Fiorinia phantasma

XML Treatment for Fiorinia pinicola

XML Treatment for Fiorinia proboscidaria

XML Treatment for Fiorinia theae

## Supplementary materials

Supplementary material 1 Figures S1–S4 Data type Phylogenetic trees and images (docx. file) Explanation note **Figure S1.** Comparison of field habitus of first-instar exuviae overlapping second-instar exuviae of seven *Fiorinia* species occurring in the USA. **Figure S2.** Maximum likelihood bootstrap consensus tree of the subtribe Fioriniina based on 28S, EF1-α, 5’-COI, 3’-COI, and COII. The clade highlighted in solid red indicates a monophyletic *Fiorinia*. **Figure S3.** Maximum likelihood bootstrap consensus tree of the subtribe Fioriniina based on 28S. The clade highlighted in red contains *Fiorinia*. Red dashed line indicates two *Fiorinia* sp. isolates placed with *Pseudaulacaspis* species. Dashed black lines indicate non-*Fiorinia* species placed in the *Fiorinia* clade. Bootstrap support values equal or greater than 50 are indicated on the tree. **Figure S4.** Neighbor-joining tree of Diaspididae 5’-COI barcodes. Terminal taxa are labeled to their narrowest identification-level. Numbers in parentheses after terminal taxa indicate how many sequences are represented in each cluster. The cluster of *Fiorinia* species is highlighted in red. Bootstrap support values greater than 75 are indicated on the tree. Nodes with 100 percent bootstrap support are indicated by a “*”. https://binary.pensoft.net/file/603823This dataset is made available under the Open Database License (http://opendatacommons.org/licenses/odbl/1.0/). The Open Database License (ODbL) is a license agreement intended to allow users to freely share, modify, and use this Dataset while maintaining this same freedom for others, provided that the original source and author(s) are credited.Muhammad Z. Ahmed, Matthew R. Moore, Eric A. Rohrig, Cindy L. McKenzie, Di Liu, Jinian Feng, Benjamin B. Normark, Douglass R. Miller

Supplementary material 2 Table S1 Data type Sequences accession numbers (excel table) Explanation note **Table S1.** Taxa, isolates, and GenBank accessions used for phylogenetic analyses. https://binary.pensoft.net/file/603824This dataset is made available under the Open Database License (http://opendatacommons.org/licenses/odbl/1.0/). The Open Database License (ODbL) is a license agreement intended to allow users to freely share, modify, and use this Dataset while maintaining this same freedom for others, provided that the original source and author(s) are credited.Muhammad Z. Ahmed, Matthew R. Moore, Eric A. Rohrig, Cindy L. McKenzie, Di Liu, Jinian Feng, Benjamin B. Normark, Douglass R. Miller

Supplementary material 3 Table S2. PCR primers and thermocycling conditions. Data type PCR primers and thermocycling conditions (docx. file) Explanation note **Table S2.**PCR primers and thermocycling conditions. All PCR reactions were performed with 2-minute denaturation at 95⁰ C. Each subsequent cycle consists of a 30 second denaturation at 98⁰ C, a 30 second annealing step with temperature given below, and a 45 secs extension at 72⁰ C., end with a single 7 -minute extension at 72⁰ C. Primer sequences are given from 5’ to 3’. https://binary.pensoft.net/file/603825This dataset is made available under the Open Database License (http://opendatacommons.org/licenses/odbl/1.0/). The Open Database License (ODbL) is a license agreement intended to allow users to freely share, modify, and use this Dataset while maintaining this same freedom for others, provided that the original source and author(s) are credited.Muhammad Z. Ahmed, Matthew R. Moore, Eric A. Rohrig, Cindy L. McKenzie, Di Liu, Jinian Feng, Benjamin B. Normark, Douglass R. Miller

Supplementary material 4 Raw data Data type fas. file Explanation note Raw data file containing aligned sequences used in our phylogenetic analysis. https://binary.pensoft.net/file/603826This dataset is made available under the Open Database License (http://opendatacommons.org/licenses/odbl/1.0/). The Open Database License (ODbL) is a license agreement intended to allow users to freely share, modify, and use this Dataset while maintaining this same freedom for others, provided that the original source and author(s) are credited.Muhammad Z. Ahmed, Matthew R. Moore, Eric A. Rohrig, Cindy L. McKenzie, Di Liu, Jinian Feng, Benjamin B. Normark, Douglass R. Miller
