# Supplementary material for: Taxonomic and identification review of adventive Fiorinia Targioni Tozzetti (Hemiptera, Coccomorpha, Diaspididae) of the United States
Source: Zookeys. 2021 Oct 27;1065:141–203. doi: 10.3897/zookeys.1065.69171 (PMC9616077; doi:10.3897/zookeys.1065.69171)
Supplement: Supplementary material 1 — Figures S1–S4 [file zookeys-1065-141-s001.docx]

**Supplementary files**


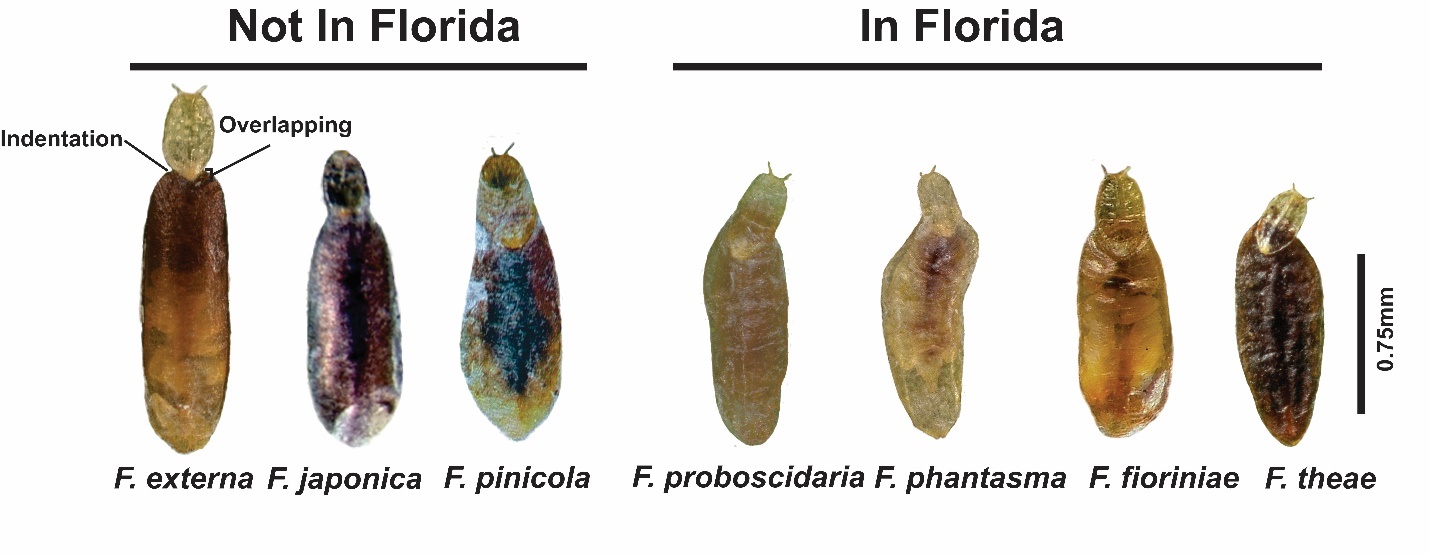
 **Supplementary Figure 1.** Comparison of field habitus of first-instar exuviae overlapping second-instar exuviae of seven *Fiorinia* species occurring in the USA.

**
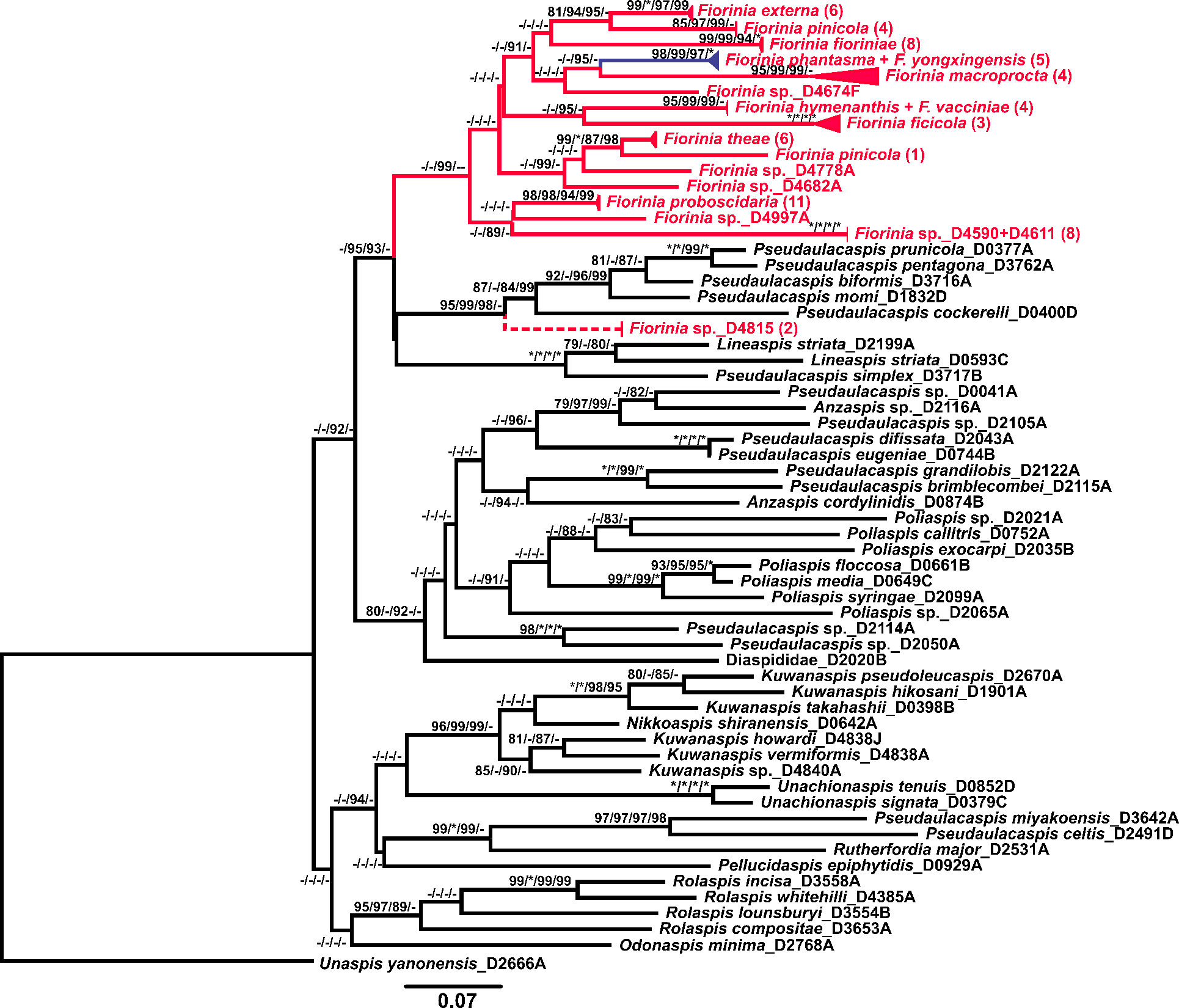
**

**Supplementary Figure 2.** Maximum likelihood bootstrap consensus tree of the subtribe Fioriniina based on 28S, EF1-α, 5’-COI, 3’-COI, and COII. The clade highlighted in solid red indicates a monophyletic *Fiorinia*.

**
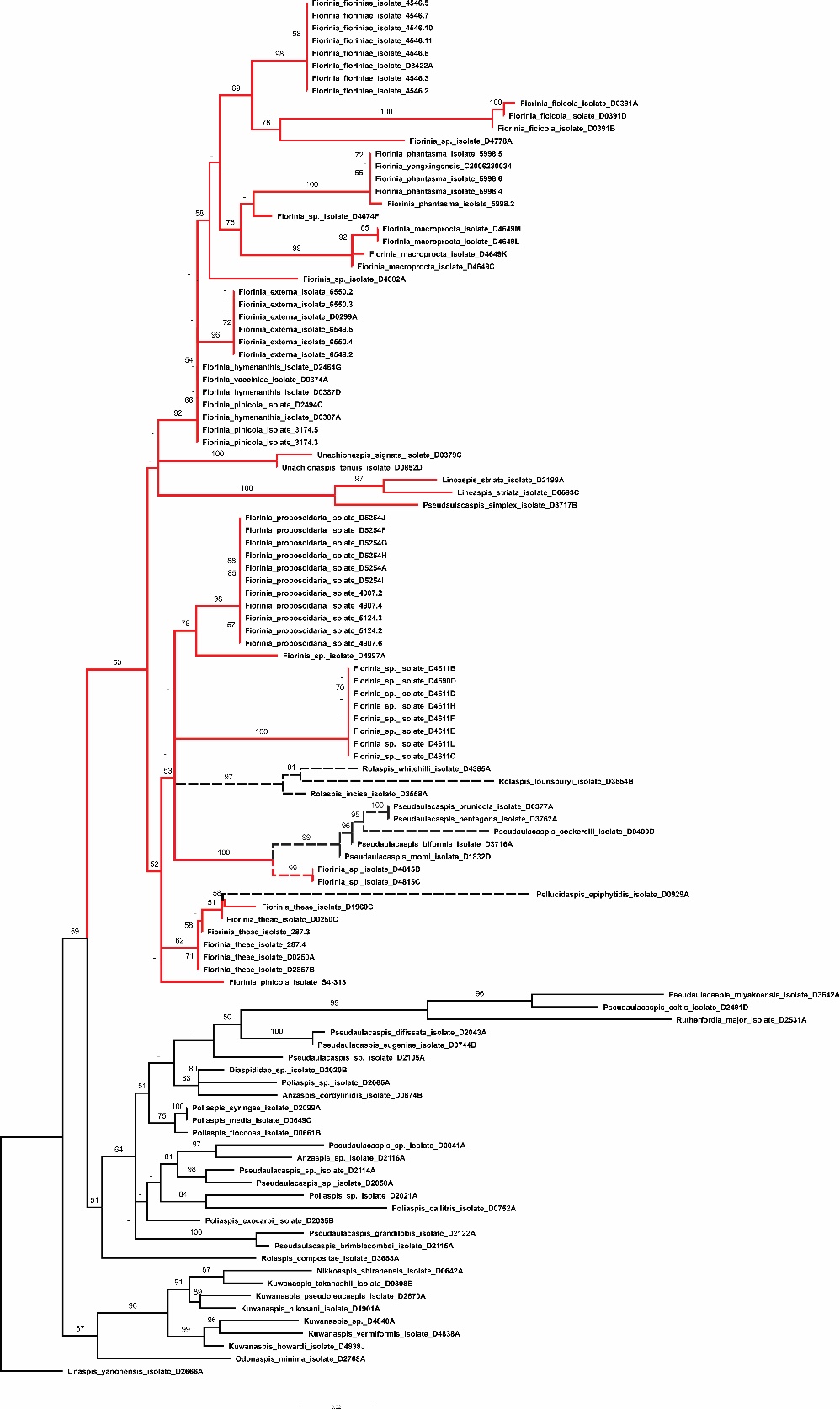
**

**Supplementary Figure 3.** Maximum likelihood bootstrap consensus tree of the subtribe Fioriniina based on 28S. The clade highlighted in red contains *Fiorinia*. Red dashed line indicates two *Fiorinia* sp. isolates placed with *Pseudaulacaspis* species. Dashed black lines indicate non-*Fiorinia* species placed in the *Fiorinia* clade. Bootstrap support values equal or greater than 50 are indicated on the tree.


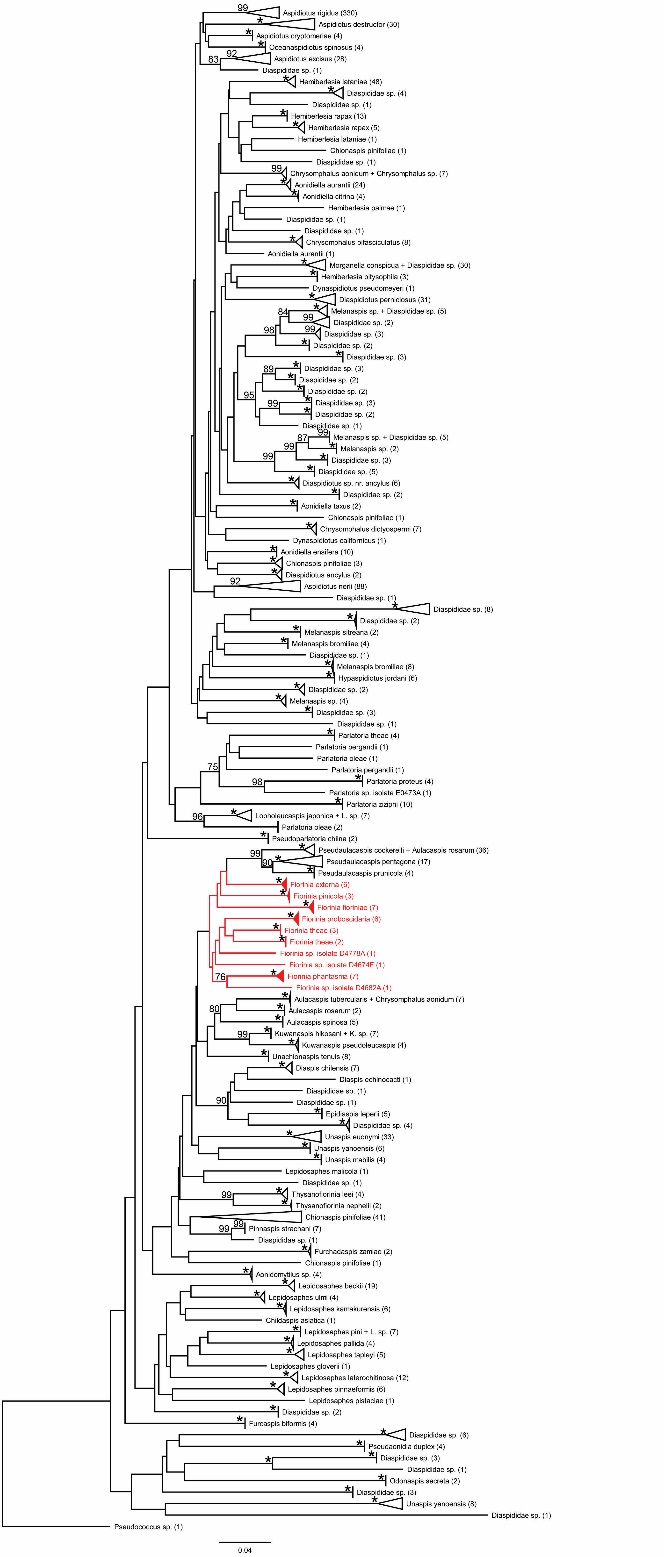


**Supplementary Figure 4.** Neighbor-joining tree of Diaspididae 5’-COI barcodes. Terminal taxa are labeled to their narrowest identification-level. Numbers in parentheses after terminal taxa indicate how many sequences are represented in each cluster. The cluster of *Fiorinia* species is highlighted in red. Bootstrap support values greater than 75 are indicated on the tree. Nodes with 100 percent bootstrap support are indicated by a “*”.
