# Supplementary material for: Taxonomic and identification review of adventive Fiorinia Targioni Tozzetti (Hemiptera, Coccomorpha, Diaspididae) of the United States
Source: Zookeys. 2021 Oct 27;1065:141–203. doi: 10.3897/zookeys.1065.69171 (PMC9616077; doi:10.3897/zookeys.1065.69171)
Supplement: Supplementary material 3 — Table S2. PCR primers and thermocycling conditions. [file zookeys-1065-141-s003.docx]

**Supplementary Table 2.** PCR primers and thermocycling conditions. All PCR reactions were performed with 2-minute denaturation at 95⁰ C. Each subsequent cycle consists of a 30 second denaturation at 98⁰ C, a 30 second annealing step with temperature given below, and a 45 secs extension at 72⁰ C., end with a single 7 -minute extension at 72⁰ C. Primer sequences are given from 5' to 3'.

| **Gene Region** | **Primer Name and Sequence** | **Primer Citation** | **Annealing Temp** | **Extension Time** |
| --- | --- | --- | --- | --- |
| 28S | 28s_s3660:  GAGAGTTMAASAGTACGTGAAAC | Gruwell et al. (2007) | 48°C | 45 secs |
|  | 28s_a335:  TCGGARGGAACCAGCTACTA |  |  |  |
| EF-1α | EF-1α(a):  GATGCTCCGGGACAYAGAG | Andersen et al. (2010) | 62°C | 30 secs |
|  | EF2rod:  ATGTGAGCGGTGTGGCAATCCAA |  |  |  |
| COI | PcoF1: CCTTCAACTAATCATAAAAATATYAG | Park et al. (2010) | 50°C | 30 secs |
|  | LEP-R1: TAAACTTCTGGATGTCCAAAAA | Hebert et al. (2004) |  |  |
|  | HCO2198:  TAAACTTCAGGGTGACCAAAAAATCA | Folmer et al. (1994) |  |  |
